# Dosing PPARγ Activation With Light Using Fast Relaxing Photoswitches

**DOI:** 10.1002/anie.7525141

**Published:** 2026-08-19

**Authors:** Loris Knümann, Alba Rodriguez‐Paniagua, Martin Reynders, Silke Duensing‐Kropp, Oliver Thorn‐Seshold, Vasily Morozov, Julian A. Marschner, Daniel Merk

**Affiliations:** ^1^ Department of Pharmacy Ludwig‐Maximilians‐Universität München Munich Germany; ^2^ Faculty of Chemistry and Food Chemistry TU Dresden Dresden Germany

**Keywords:** adipogenesis, lipid metabolism, peroxisome proliferator‐activated receptor gamma, photoswitch, transcription factor

## Abstract

The transcription factor PPARγ regulates glucose and lipid homeostasis, and its activation, e.g., with glitazones, has antidiabetic, insulin‐sensitizing activity, but also adverse effects which possibly arise from overstimulation. Here we developed a novel high‐precision tool to enable light‐dosed PPARγ activation. Structure‐guided incorporation of a photoswitchable diazene motif into a PPARγ ligand and molecular fine‐tuning provided potent, light‐activated PPARγ agonists with millisecond to multiple‐hour half‐lives, suitable for biological research applications. Phenotypic effects of light‐dosed PPARγ activation in an in vitro model of adipogenesis suggested time‐dependent importance of the receptor in adipocyte development and illustrated the unprecedented precision of controlling PPARγ activity with light‐activated chemical tools.

## Graphical abstract

To achieve spatiotemporal control over PPARγ activity, we designed the fast‐relaxing photoswitchable agonist **6** that selectively activates PPARγ in its light‐induced *Z*‐state and relaxes within seconds (t_1/2_ 47 s) to the inactive *E*‐state. This unique profile allowed light‐dosed receptor activation in in vitro settings, enabling biological studies on PPARγ with unprecedented precision.

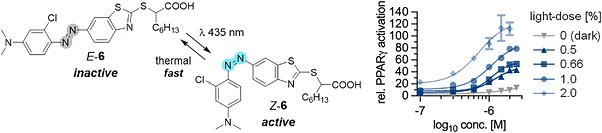

## 1 Introduction

The peroxisome proliferator‐activated receptor γ (PPARγ) [1] is a key transcriptional regulator of metabolic balance and an important drug target. It is a ligand‐activated transcription factor involved in glucose and lipid metabolism, counteracting insulin resistance, and controlling adipose tissue development and maintenance [2, 3]. PPARγ agonist drugs (thiazolidinediones (TZDs), also known as glitazones) [4] mediate insulin‐sensitizing effects and are effective oral antidiabetics [1, 5, 6]. However, their value and relevance as drugs are compromised by severe adverse effects [7, 8], which may be due to the persistent, strong PPARγ activation that these long‐acting full‐agonist TZDs induce [6]. Current approaches to pharmacological PPARγ modulation therefore focus on partial agonists and selective modulators (sPPARγM) as well as alternative (allosteric) site ligands, some of which have shown therapeutic efficacy with improved safety [6, 9, 10, 11, 12, 13, 14]. However, the endogenous ligands of nuclear hormone receptors are typically either high‐affinity short‐lived hormones or low‐affinity metabolic intermediates and products [1, 15, 16, 17]. Therefore, it is plausible that more physiological control over PPARγ activity would be achieved by short‐lived activators, i.e., by gaining temporal control over PPARγ activation, which motivated the development of appropriate tools in this study to test this hypothesis.

Reversibly photoswitchable ligands are ideally suited to establish temporal control over protein dynamics and activity [18, 19]. They can be obtained by, e.g., incorporating an azobenzene motif into a bioactive molecule [20, 21, 22]. This concept has been successfully implemented for ligands of many protein types [20, 21, 23, 24], including nuclear receptors [25, 26, 27]. We have previously explored TZDs and related scaffolds for the development of light‐controlled PPARγ modulators [28], yielding initial leads with low micromolar potency and binding affinity. However, activity differences between the *E*‐ and *Z*‐isomers remained low ( < 5‐fold) for these scaffolds, likely due to the large and dynamic ligand binding pocket of PPARγ [29] which can accommodate diverse ligand chemotypes and geometries [30, 31]. No photoswitchable tools with sufficient activity difference between the *E*‐ and *Z*‐isomers to enable effective studies were yet obtained, and despite its significant role in physiology and pharmacology, PPARγ remained inaccessible to photopharmacological evaluation.

In particular, light‐activated PPARγ agonists would be desirable to unlock high‐precision studies, e.g., to discriminate the impact of short‐ versus long‐lived full agonism, against fine‐tuned (partial) agonism of intermediate duration. Here, we developed this missing type of high‐precision tool for temporal control over PPARγ activity. We designed a potent photoswitchable PPARγ agonist scaffold that is only active in the light‐induced *Z*‐configuration and tuned its photophysical characteristics to give a series of cognate activators with millisecond to hour half‐lives. These chemical tools enable light‐dosed PPARγ activation, controlling this key transcription factor with unprecedented temporal resolution.

## 2 Results and Discussion

The PPARγ agonist **1** [32] features a unique binding mode in the ligand pocket (Figure 1a) with the aryl‐aryl sulfonamide binding in a *Z‐*stilbenoid‐like conformation, in order to form polar contacts to the activation function 2 (AF2) in helix 12, and the 2,4‐dichlorophenyl motif placed in an adjacent hydrophobic sub‐pocket. Since this *Z‐*like conformation and direct ligand contacts to the receptor's key activation residues Tyr327 and His449 are essential for binding to PPARγ, we reasoned that an isosteric photoswitchable azobenzene motif in this ligand region could be used to control PPARγ binding and agonist activity. We hypothesized that its thermodynamically favored *E*‐isomer (“dark state”) would hinder binding, while the light‐activated *Z*‐isomer would provide an optimal pre‐formed geometry to place the 2,4‐dichlorophenyl residue in its hydrophobic binding pocket. We therefore designed the “azolog” **2** as a potential *Z*‐active, light‐activated PPARγ agonist (Figure 1b).

**FIGURE 1:**
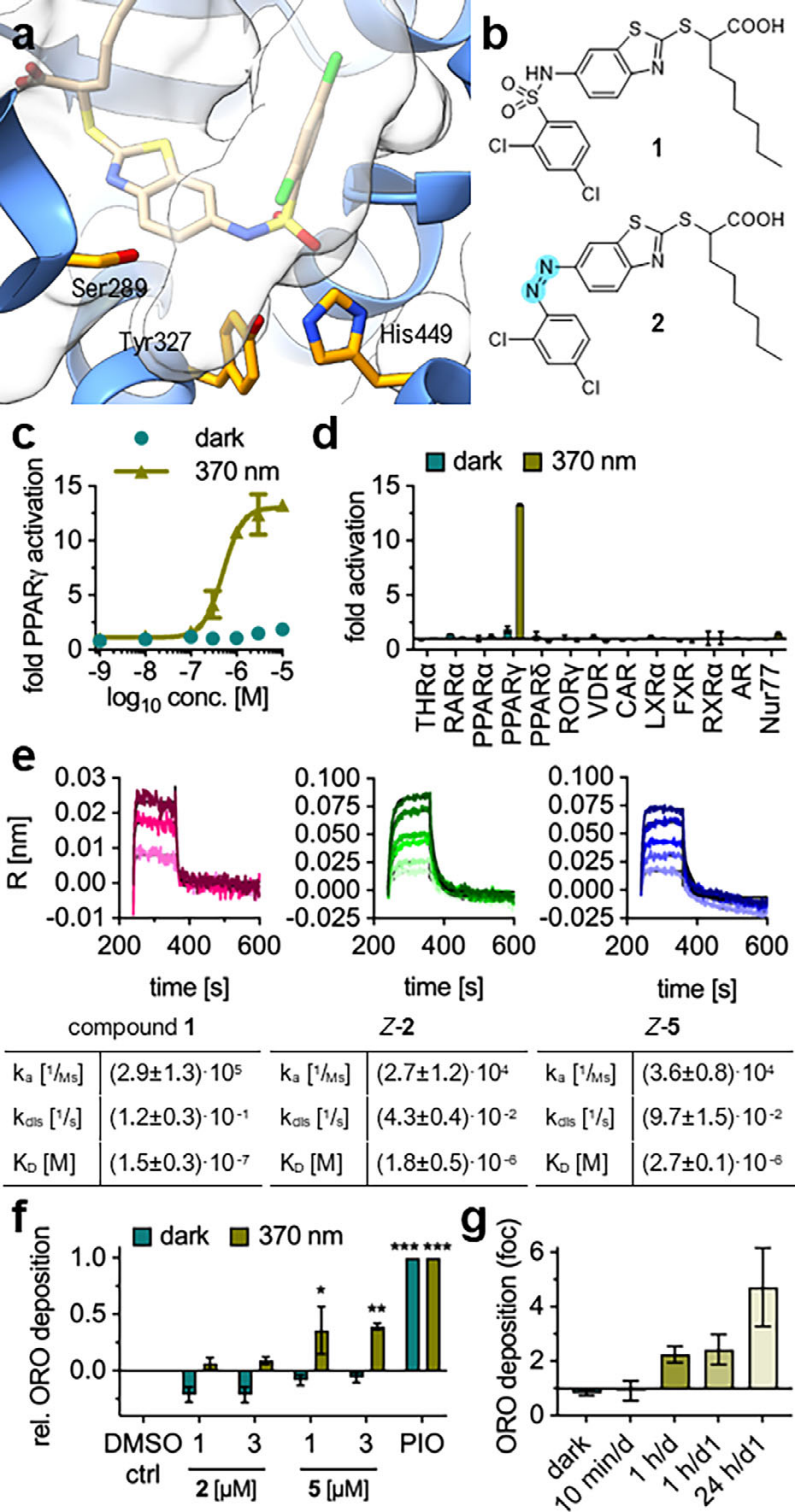
Structure‐based design of a light‐activated PPARγ agonist. (a) Binding mode of the template agonist **1** in the PPARγ ligand binding domain (pdb ID: 6t6b) [32] with the *Z*‐like sulfonamide engaging polar contacts with Tyr327 and His449 in the receptor's activation function. (b) Design of azolog **2** as a light‐activated PPARγ agonist. (c) Dose‐response curves of dark‐adapted *E*‐**2** and light‐activated mostly‐*Z*‐**2** (EC_50_ 0.50 ± 0.09 µM) in a Gal4‐PPARγ hybrid reporter gene assay (data as mean ± S.E.M., *n* = 4). (d) Selectivity profiles of *E*‐**2** and mostly‐*Z*‐**2** (10 µM) on nuclear receptors related to PPARγ (data as mean ± S.E.M., *n* = 3). (e) PPARγ binding of **1**, *Z*‐**2** and *Z*‐**5** evaluated by biolayer interferometry (BLI). Concentration ranges (light to dark color): **1**: 0.75–1.5 µM; *Z*‐**2**: 0.8–2.4 µM; *Z*‐**5**: 1.2–8.0 µM. Data are the mean ± SD of two independent replicates. Data for *E*‐**2** and *E*‐**5** are shown in Figure S8. (f) Effects of **2** and **5** (1 or 3 µM) in a 21‐day adipocyte differentiation assay (data as mean ± S.E.M. rel. Oil Red‐O deposition versus pioglitazone (PIO, 1 µM); 24 h illumination on day 1 of each differentiation cycle; *n* = 3; * *p* < 0.05, ** *p* < 0.01, *** *p* < 0.001 (vs. the corresponding DMSO ctrl, ANOVA with Bonferroni multiple comparisons test)). (g) Light‐dose dependent effects of **5** (3 µM) on adipogenesis (see Figure S10 for a schematic illustration of the model). Data are the mean ± S.E.M. Oil Red‐O deposition; *n* = 3. A CellDisco [33] was used in (c,d,f,g) at the indicated wavelength for pulsed illumination (100 ms/10 s) to maintain the respective *Z*‐isomers.

This candidate PPARγ photoswitch **2** was prepared from the 6‐aminobenzothiazole **8** (obtained from benzo[d]thiazole‐2‐thiol **7**, as described previously [32]). **8** was hydrolyzed to the free carboxylate **9** and then used to prepare **2** by Baeyer‐Mills condensation with the nitrosobenzene derived from oxone oxidation of aniline **10** (later analogs **3**–**6** were also prepared from **9**: **4** and **5** were also prepared via Baeyer‐Mills condensations with the anilines **11** and **13**, and the electron‐rich *para‐*dimethylamino analogs **3** and **6** were prepared by diazotisation of **9** and subsequent azo coupling to the appropriate *para‐*dimethylanilines (**14**, **15**); Scheme 1).

**SCHEME 1:**
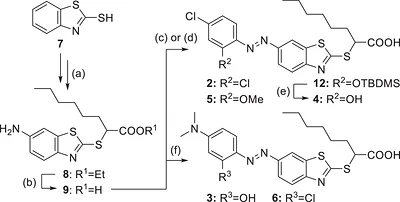
Synthesis of **2–6**. Reagents & Conditions: (a) Prepared according to ref [32].; (b) LiOH, H_2_O/THF, rt, overnight, 73%; (c) 2,4‐dichloroaniline (**10**) or 4‐chloro‐2‐methoxyaniline (**11**), oxone, CH_2_Cl_2_/H_2_O, rt, 2 h; then **9**, HOAc, rt, 60 h, 32%–47%; (d) 2‐((tert‐butyldimethylsilyl)oxy)‐4‐chloroaniline (**13**), m‐CPBA, CH_2_Cl_2_, 0°C, 60 min, then **9**, HOAc, rt, 60 h; (e) TFA/H_2_O, rt, 2 h, 44% over two steps; (f) **9**, HCl, NaNO_2_, H_2_O/THF, 0°C, 45 min, then NaOAc, 3‐(*N*,*N*‐dimethylamino)phenol (**14**) or 3‐chloro‐*N*,*N*‐dimethylaniline (**15**), H_2_O/THF, rt, overnight, 27%–32%.

We first tested **2** for potential PPARγ photoswitch activity in cellular reporter gene assays (Figure 1c), which showed strong, sub‐micromolar PPARγ agonism by the light‐activated *Z*‐**2** (EC_50_ 0.50 ± 0.09 µM; Table 1), while the dark‐adapted *E*‐**2** caused no appreciable PPARγ activation even up to 10 µM (i.e. 20‐fold EC_50_ of *Z*‐**2**). Selectivity profiling under dark (*E*) and light‐activated (*Z*) conditions revealed high PPARγ selectivity, with no other *E* or *Z* activity on related lipid‐activated nuclear receptors (Figure 1d). Except for a slight bathochromic shift (λ_max,_ *_E_* 377 nm), **2** had photophysical properties rather typical for azobenzene switches (Figure S1), with strongly wavelength‐dependent photostationary state *E*:*Z* ratios, repeatable photoswitching over multiple cycles without fatigue under alternating illuminations, and a moderate *Z→E* thermal relaxation half‐life (7 h, 37°C). Taken together, **2**’s good *E*⇄*Z* photoswitchability and the strong, potent, PPARγ‐selective cellular activity of its *Z‐*isomer supported its suitability as a light‐controllable agonist scaffold.

**TABLE 1:** Photophysical and PPARγ modulator profiles of **2**–**6**.

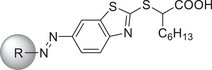				PPARγ: EC_50_ (max. act.)^b^	
ID	R	t_1/2_ ^a^	λ_max_ ^a^	Dark	Light‐activated [λ]^c^
**2**	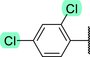	7 h	377 nm	> 10 µM	0.50 ± 0.09 µM (13 ± 1‐fold) [370 nm]
**3**	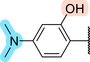	< 1 s	481 nm	3.0‐fold act. at 3 µM^d^	3.5‐fold act. at 3 µM^d^ [425 nm]
**4**	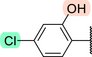	< 1 s	389 nm	3.0‐fold act. at 3 µM^d^	3.1‐fold act. at 3 µM^d^ [370 nm]
**5**	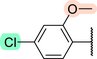	5 h	391 nm	> 10 µM	0.60 ± 0.06 µM (38 ± 1‐fold) [370 nm]
**6**	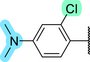	47 s	449 nm	> 10 µM	1.1 ± 0.1 µM (14 ± 2‐fold) [435 nm]

^a^ Full photophysical characterization is shown in Figures S1–S6.

^b^ Data (mean ± S.E.M.; *n* ≥ 4) from a Gal4‐PPARγ hybrid reporter gene assay. Dose‐response curves are presented in Figure S7.

^c^ Compounds were illuminated with a CellDisco [33] for 100 ms/10 s at the indicated wavelength.

^d^ EC_50_ was not determined; activity at the highest tested concentration is shown.

We next aimed to optimize this scaffold towards a suite of photoswitchable analogs with similar pharmacology but suiting different assay timescales, as a toolkit for high‐precision studies on PPARγ. Further analysis of the PPARγ‐**1** complex (Figure 1a) indicated that the sub‐pocket binding the 2,4‐dichlorophenyl motif could accommodate larger groups in the 4‐position, and that polar 2‐substituents might engage additional H‐bond contacts with the activation triad residues (Tyr327, His449, Tyr473) to achieve stronger activation. We thus designed, synthesized, and evaluated analogs of **2** bearing alternative 2‐ and 4‐substituents chosen to modify the *Z‐*isomer half‐lives (**3**–**5**, Table 1, Figure S7). Matching expectations for azo‐hydrazone tautomerism [34, 35, 36, 37], 2‐hydroxy substitutions resulted in extremely fast cell‐free *Z→E* thermal relaxation (**3**: essentially *E‐*only; **4**: *Z→E* half‐life < 0.1 s in 50% DMSO; Figures S2 and S3), and no relevant cellular activity. Instead, the non‐tautomerisable 2‐methoxy derivative **5** retained a workable *Z→E* half‐life (5 h in DMSO; Figure S4). Outstandingly, its *Z‐*isomer had even higher PPARγ agonistic efficacy (38‐fold) than **2**, while retaining its sub‐micromolar potency, and *E*‐inactivity: thus **5** emerged as an excellent precision tool for PPARγ studies. As some classes of azobenzenes are prone to addition or reduction reactions with endogenous reducing nucleophiles such as glutathione (GSH) [38], and as differences of degradation are known for their *E* and *Z* isomers, we probed the stability of **5** against 2 mM GSH at 37°C in all‐*E* or mostly‐*Z* (constant irradiation at 385 nm) samples. No degradation could be detected within 1 h (Figure S6), supporting the suitability of **5** for clean application as *E/Z‐*switch in biological systems.

Biolayer interferometry (BLI) verified binding of the light‐activated agonists *Z*‐**2** and *Z*‐**5** but not *E*‐**2** and *E*‐**5** to PPARγ (Figures 1e and S8). In line with their similar in vitro potencies, *Z*‐**2** and *Z*‐**5** displayed similar binding affinity, and approx. 10‐fold reduced affinity compared to the sulfonamide lead **1**. Notably, both compounds exhibited slower association and dissociation rates than **1**, suggesting a kinetically more constrained binding process and a more stable ligand–receptor complex once formed. Since *Z*‐**2** and *Z*‐**5** showed similar binding properties, the higher activation efficacy of *Z*‐**5** likely results from enhanced stabilization of the PPARγ ligand binding domain active conformation [29, 39, 40, 41]. Although likely too distant for hydrogen bond formation, the methoxy group of *Z*‐**5** binds in proximity to the Tyr–His–Tyr activation triad and may better preserve the local polar environment.

To characterize the performance of **5** in light‐controlled PPARγ activation in biological settings, we studied its impact on adipocyte differentiation. Functional fat tissue is essential for metabolic and energy homeostasis and crucially requires PPARγ activity both to orchestrate adipocyte differentiation and to drive storage of fat in the resulting differentiated adipocytes [42, 43]. We employed a model of adipogenesis and fat deposition [44] based on adipose‐derived stem cells (ASCs) which develop into mature adipocytes in vitro under cyclic treatment with insulin, isobutylmethylxanthine, dexamethasone, and a PPARγ agonist (e.g., pioglitazone). Adipocyte differentiation and fat storage can then be detected by staining (Oil Red‐O) [43]. Significant adipogenesis was evident after 21 days of the cyclic differentiation treatment, including either pioglitazone or the light‐activated PPARγ agonist **5** (Figure 1f). In line with photoactivation, the effect of **5** was light‐dependent and absent in the dark. Under pulsed illumination with a CellDisco [33] (100 ms on per 10 s off), **5** reached approx. 40% of the effect of pioglitazone, highlighting its ability to mediate light‐dependent PPARγ activation. The less efficient lead **2** had no detectable effect under dark and illuminated conditions.

With the light‐activated PPARγ agonist **5** in hand, we tested whether its attractive profile could be exploited for preliminary experiments on the timing and duration of PPARγ stimulation required for adipogenesis (Figure 1g). Intriguingly, we observed timing‐ and light‐dose‐dependent differences in adipocyte development under treatment with **5**. While daily short‐term pulsed illumination for 10 min was insufficient, as little as 60 min daily caused an increase in adipocyte differentiation. Interestingly, the effect of 60 min light on day 1 of each treatment cycle resembled the effect of daily illumination for the same duration, suggesting that PPARγ activity was most important early in adipogenic stimuli. In line with this, 24 h illumination of **5** on day 1 of each cycle substantially enhanced adipocyte differentiation.

These results demonstrated that the light‐activated agonist scaffold was a suitable design for tools enabling unprecedented precision in PPARγ activation. Nevertheless, we envisioned reaching even finer time resolution of PPARγ activity photocontrol by photophysical tuning to reach a faster‐relaxing analog that is only active during illumination, and can be activated at less cell‐damaging, longer wavelengths [36]. We thus introduced a 4‐dimethylamino motif (**6**, Figure 2a), giving a half‐life of 47 s (in PBS:DMSO mixture) and an absorbance maximum at 449 nm (Figure 2b) that match well with this intended profile. In vitro profiling of **6** corroborated PPARγ agonism by the light‐induced *Z*‐isomer, with no effect in the dark (*E*). Notably, the fast relaxation properties of **6** (Figure 2c) allowed intensiometric power‐dosing of its activation efficacy (Figure 2d): e.g., pulsed illumination for 100 ms per 20 s (0.5% light‐dose (“duty cycle”)) reached 50% of maximum PPARγ activation compared to pioglitazone, with increased light‐dose consistently increasing efficacy, e.g., reaching 126% activation at 2% duty cycle. PPARγ activity induced by **6** thus correlated well with the time‐average light intensity, while the EC_50_ values remained stable (0.7–1.2 µM), indicating that a single concentration of **6** can be used for intensity‐dosed PPARγ activity control. As expected, the slow‐relaxing photoswitches **2** and **5** were not suitable for intensity‐dosed PPARγ control (Figure S9). *E/Z‐* **6** was found to be stable against GSH in vitro (Figure S6), making the fast‐relaxing photoswitchable agonist **6** an unprecedented tool for precise intensity‐dependent control over PPARγ.

**FIGURE 2:**
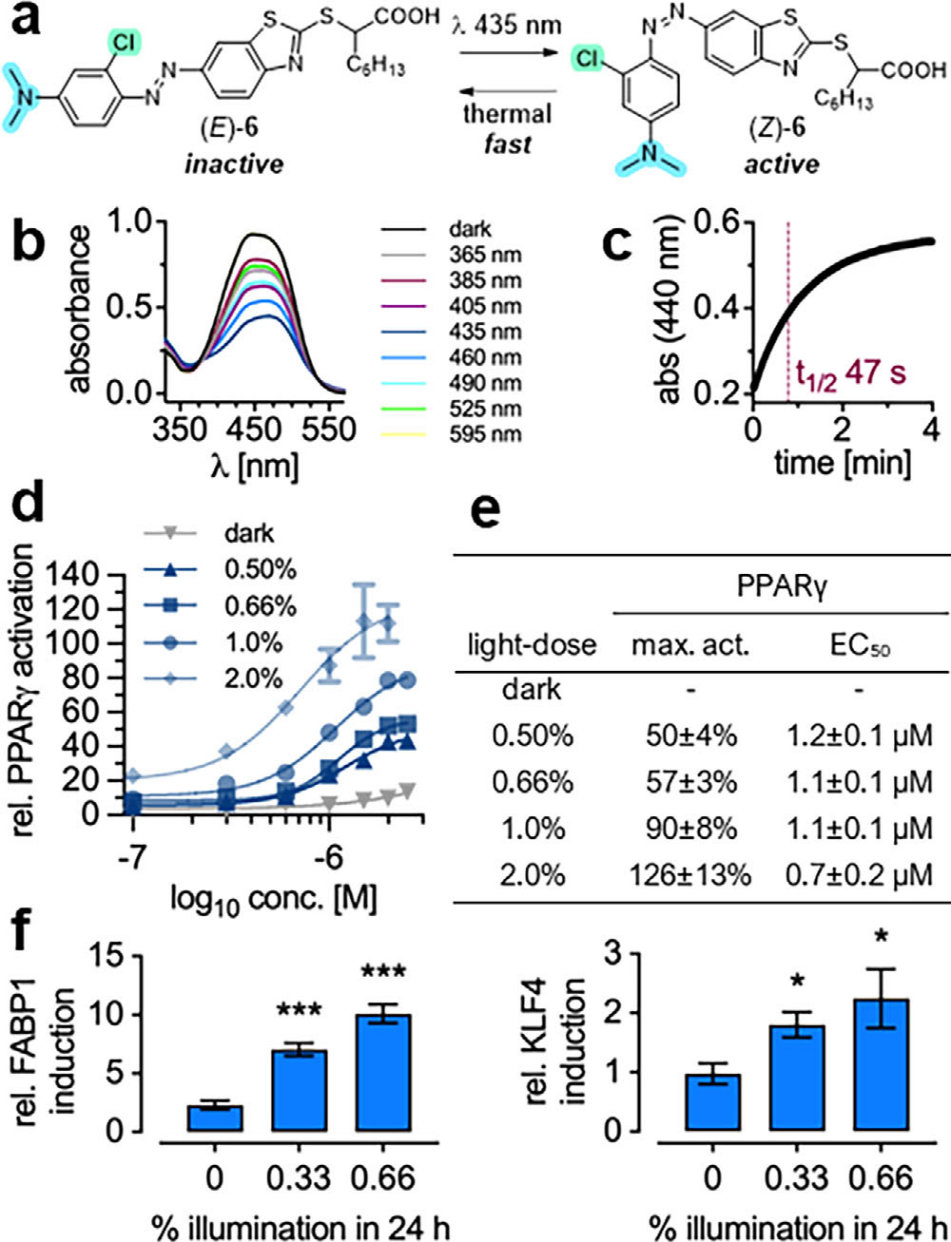
The photoswitchable agonist **6** enables high‐precision optical control of PPARγ. (a) Chemical structure of **6**. (b) UV‐vis spectra of **6** in DMSO in the dark‐adapted state and after illumination at the indicated wavelengths. (c) Thermal relaxation of **6** in PBS:DMSO (1:1) with a half‐life of 47 s. (d) Dose‐response curves for PPARγ activation by **6** at different light‐doses (435 nm) with a CellDisco [33] demonstrating that the fast thermal relaxation of **6** allows for light‐dose‐dependent activation. % light‐dose refers to the DISCO on‐time; rel. PPARγ act. is relative to 1 µM pioglitazone; data are the mean ± S.E.M.; *n* ≥ 3. (e) Light‐dose‐dependent EC_50_ and % max. rel. PPARγ act. of **6**; data are the mean ± S.E.M.; *n* ≥ 3. (f) Light‐dose‐dependent effects of **6** on the expression of the PPARγ‐regulated genes fatty acid binding protein 1 (FABP1) and krüppel‐like factor 4 (KLF4). Cells were treated with **6** (1 µM) and kept in the dark, or illuminated over 24 h with the indicated light‐dose, and gene expression was determined by qRT‐PCR after 24 h. Data are the mean ± S.E.M. relative mRNA levels normalized to the DMSO control in the respective dark or illuminated condition; *n* = 3; * *p* < 0.05, *** *p* < 0.001 (vs. the corresponding DMSO‐treated control; ANOVA with Bonferroni's multiple comparisons test).

To determine the applicability of **6** as a very short‐lived PPARγ agonist in vitro and verify target engagement in a native cellular setting, we determined the effect of **6** on the expression levels of the PPARγ‐regulated fatty acid binding protein 1 (FABP1) and krüppel‐like factor 4 (KLF4) in HT29 cells. At intermediate light‐doses (0.33% and 0.66%) over 24 h, **6** caused induction of both genes with an evident correlation between the duration of illumination and the efficacy of gene induction. The short but workable half‐life of **6**, plus its strong efficacy and good potency, thus combine to allow easily intensity‐dosed PPARγ photoactivation, corroborating its value and potential as a precision photochemical tool.

## 3 Conclusion

Reversibly photoswitchable bioactive agents have unique advantages as chemical tools to control biological processes and explore the roles of molecular drug targets [20, 21, 45]. The spatio‐temporal resolution in target modulation achievable with photoswitches is particularly valuable and enables in vitro experiments with superior precision [20, 21, 45]. Despite the prominence of PPARγ as a drug target and central metabolic regulator, photopharmacology was not well developed for this receptor. Here, we closed this gap by designing a photoswitchable PPARγ agonist scaffold activating PPARγ only in its light‐induced state (*Z*), which is preferable for optical control [20]. With strong light‐activated agonist efficacy, an intermediate half‐life, and excellent stability against GSH, **5** emerged as a precision tool to study PPARγ activity with spatiotemporal control. Compared to previously described photoswitchable ligands of PPARγ [28], this agonist provides submicromolar potency and a pronounced, > 15‐fold activity difference between the *E*‐ and *Z*‐states, enabling robust ligand activation with light, as evident from its light‐dependent adipogenic effects.

The PPARγ modulator scaffold was amenable to further molecular tuning of its spectral and thermal relaxation properties. Capitalizing on this tunability, we developed the fast‐relaxing agonist **6**, which activates PPARγ in the light‐induced *Z*‐state and rapidly isomerizes back to the inactive *E*‐form within seconds, allowing an unprecedented intensiometric light‐dosing of PPARγ activation. Comparison with **5** demonstrated that slow‐relaxing active *Z*‐states could not enable this intensity‐based control, highlighting the unique potential of **6** for studies with dosed PPARγ activation. Similar short‐lived, light‐activated tools have been used to control biological events, such as cytoskeleton organization [46], neurotransmitter receptor signaling [47, 48], and neuronal activity [49], with light.

Considering future directions, we note the potential of high‐precision optical control of PPARγ, as evident in the adipogenesis assays where time‐dependent effects of **5** pointed to a temporally defined role of PPARγ in adipocyte development. While the importance of PPARγ in adipose tissue formation and function is well documented [44, 50], temporal resolution of its activity in the process of adipogenesis remains elusive. Here and in other sophisticated settings, light‐controlled PPARγ modulators hold unique potential as tools for precise temporal control. Of note, achieving similar short‐term activation by traditional means (i.e., frequent media exchange to add and remove an agonist) would not be feasible without substantial cell stress and likely be incomplete due to the accumulation of lipophilic molecules like PPARγ agonists on/in hydrophobic surfaces and compartments. Light‐dosed PPARγ activation with fine‐tuned photoswitches may thus help to close gaps in understanding of PPARγ biology.

## Author Contributions

**Loris Knümann**: investigation, validation, writing – original draft, writing – review and editing, visualization. **Alba Rodriguez‐Paniagua**: investigation, writing – review and editing. **Martin Reynders**: investigation, methodology, writing – review and editing, validation. **Silke Duensing‐Kropp**: investigation. **Oliver Thorn‐Seshold**: supervision, methodology, writing – review and editing. **Vasily Morozov**: investigation, methodology, writing – review and editing, visualization. **Julian A. Marschner**: investigation, methodology, validation, visualization, writing – review and editing, supervision. **Daniel Merk**: conceptualization, methodology, writing – original draft, writing – review and editing, supervision, visualization.

## Conflicts of Interest

The authors declare no conflicts of interest.

## Supporting information



Figures S1–S10, compound synthesis, analytical data, and in vitro assay methods are described in the Supporting Information.**Supporting File**: anie74165‐sup‐0001‐SuppMat.pdf.

## Data Availability

The data that support the findings of this study are available in the supplementary material of this article.
