# Supplementary material for: Dosing PPARγ Activation With Light Using Fast Relaxing Photoswitches
Source: Angew Chem Int Ed Engl. 2026 Aug 19;65(40):e7525141. doi: 10.1002/anie.7525141 (PMC13618307; doi:10.1002/anie.7525141)
Supplement: Supplementary file 1 [file ANIE-65-e7525141-s001.pdf]

- Supporting Information -

Dosing PPARγ Activation with Light Using Fast Relaxing Photoswitches

Loris Knümann[1#], Alba Rodriguez-Paniagua[1#], Martin Reynders[2], Silke Duensing-Kropp[1], Oliver Thorn-Seshold[2], Vasily Morozov[1], Julian A. Marschner[1], Daniel Merk[1]*

[1] Ludwig-Maximilians-Universität München, Department of Pharmacy, 81377 Munich, Germany

[2] TU Dresden, Faculty of Chemistry and Food Chemistry, 01069 Dresden, Germany

[#] L.K. and A.R. contributed equally

* daniel.merk@cup.lmu.de

Table of Contents

Supplementary Figures S2

Methods S10

Synthesis and analytical characterization of **1-6** and precursors S14

NMR spectra, IR spectra and purity analysis of **2-6** S18

Supplementary References S35

Supplementary Figures

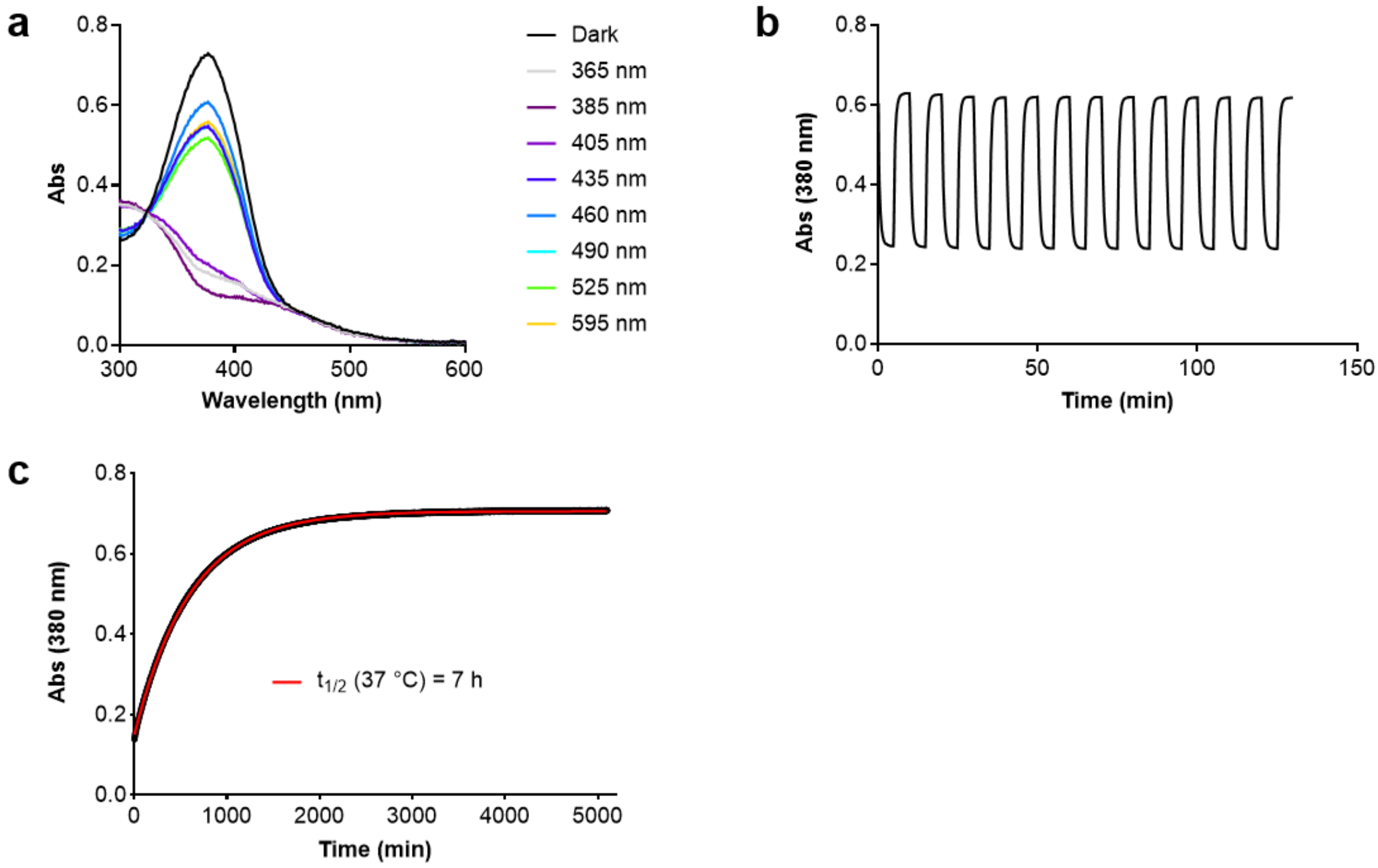

Figure S1. Photophysical characteristics of slow-relaxing **2**. (a) UV-Vis absorbance spectra of **2** in DMSO measured under irradiation at the indicated wavelengths after having reached the corresponding photostationary states (PSSs). (b) Reversible photoswitching of **2** in DMSO between mostly-*E* and mostly-*Z* PSSs, over multiple cycles, by alternating illuminations at 365 and 460 nm. (c) Thermal relaxation of ***Z*-2** in DMSO at 37 °C in the dark.

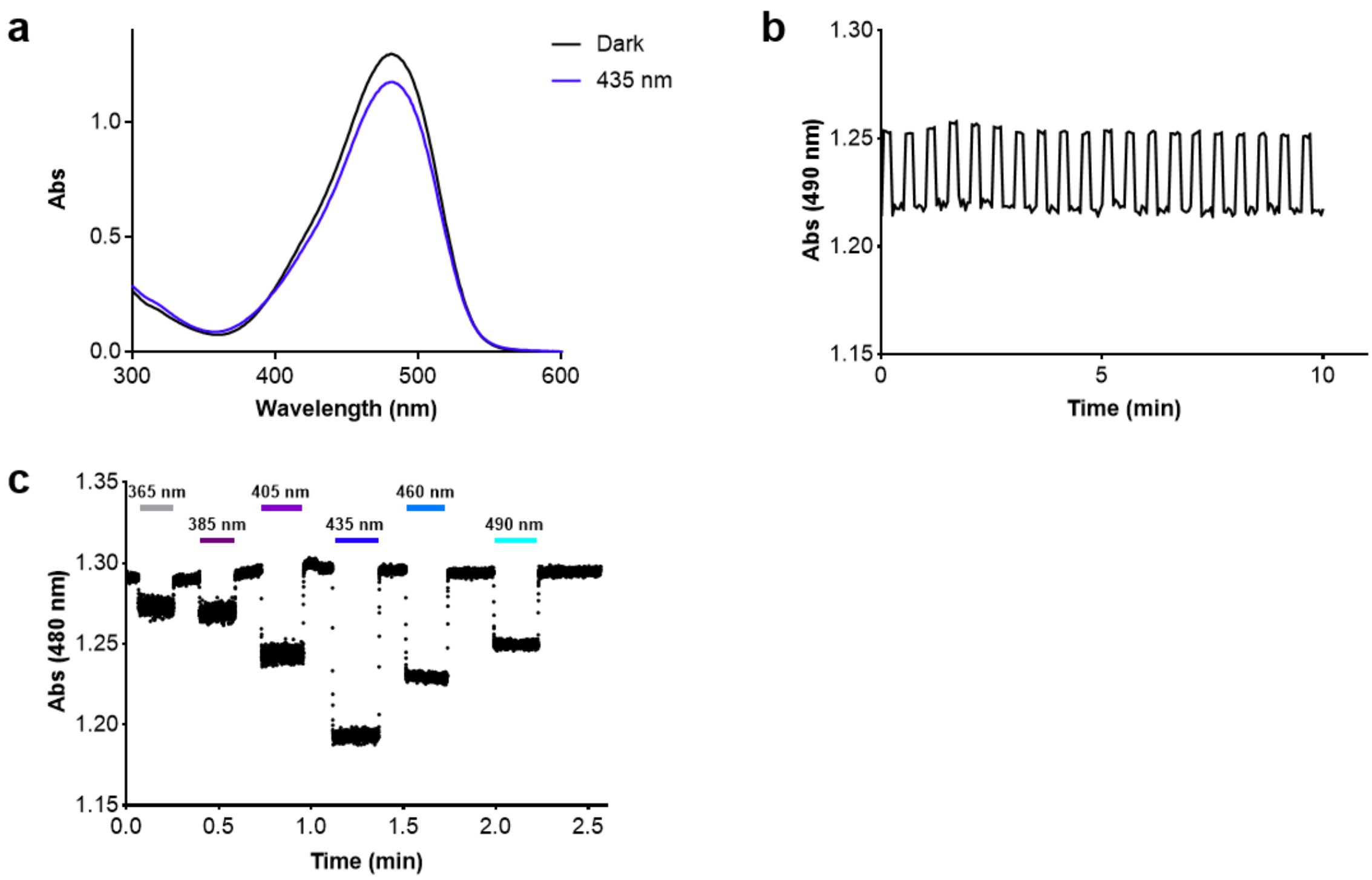

Figure S2. Photophysical characteristics of fast-relaxing **3**. (a) UV-Vis absorbance spectra of fast-relaxing **3** in DMSO measured without (dark) or with irradiation (at 435 nm, after having reached its steady state at the applied power density). (b) Reversible photoswitching of **3** in DMSO, over multiple cycles, by alternating illuminations at 385 and 525 nm. (c) Absorbance at 480 nm of **3**, in DMSO, measured while being irradiated at the indicated wavelengths (or left non-irradiated, when no irradiation is indicated).

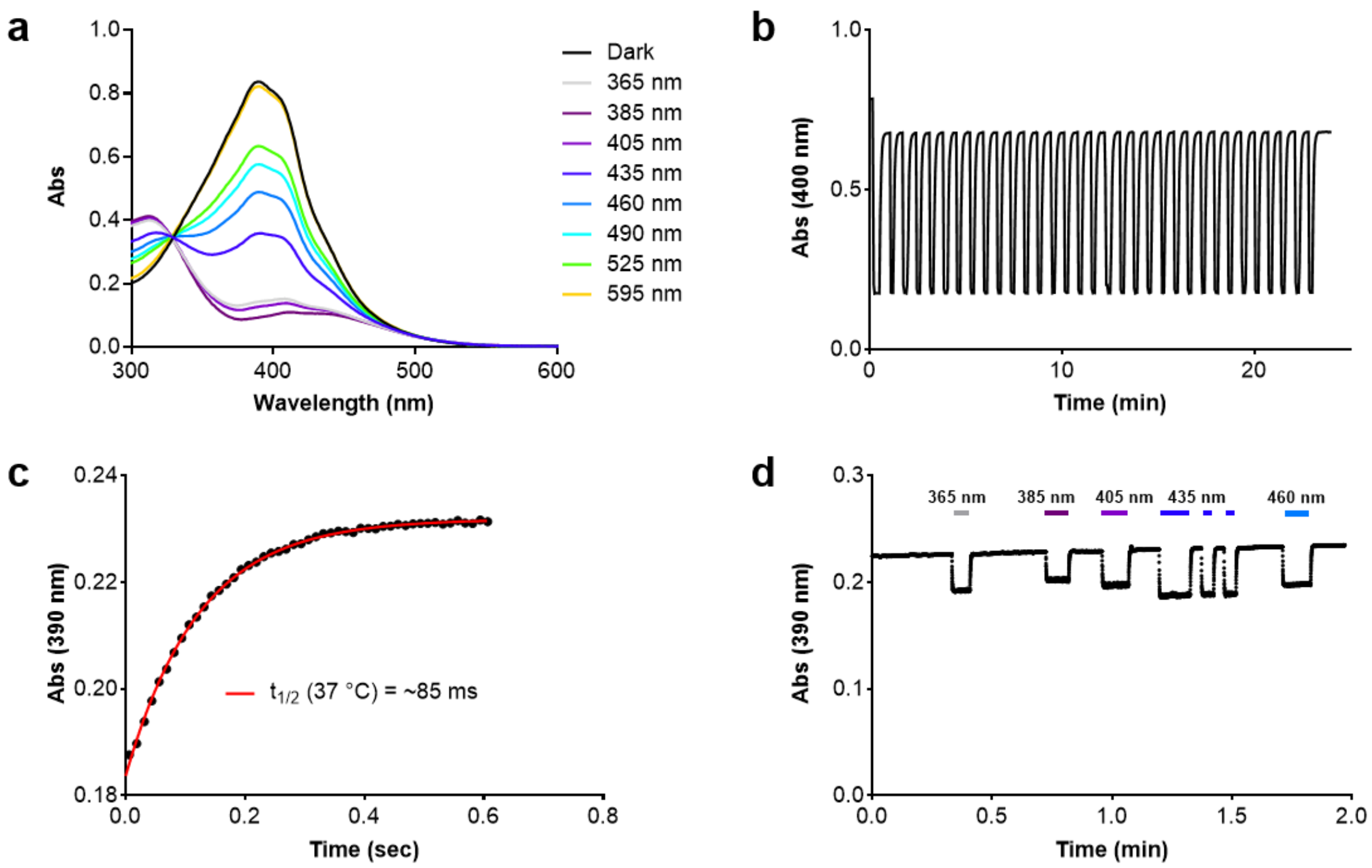

Figure S3. Photophysical characteristics of fast-relaxing **4**. (a) UV-Vis absorbance spectra of **4** in DMSO measured during irradiation at the indicated wavelengths after having reached the corresponding photostationary states (comparison of this DMSO data to the DMSO:PBS data in panel **d** illustrates that, as expected, *Z*-isomer relaxation is much slower in DMSO than in DMSO:PBS). (b) Reversible photoswitching of **4** in DMSO between mostly-*E*- and mostly-*Z* PSSs, over multiple cycles, by alternating illuminations at 385 and 525 nm. (c) Thermal relaxation of ***Z*-4** in DMSO:PBS 1:1 at 37 °C in the dark. (d) Absorbance at 390 nm of **4**, in DMSO:PBS 1:1, measured while being irradiated at the indicated wavelengths.

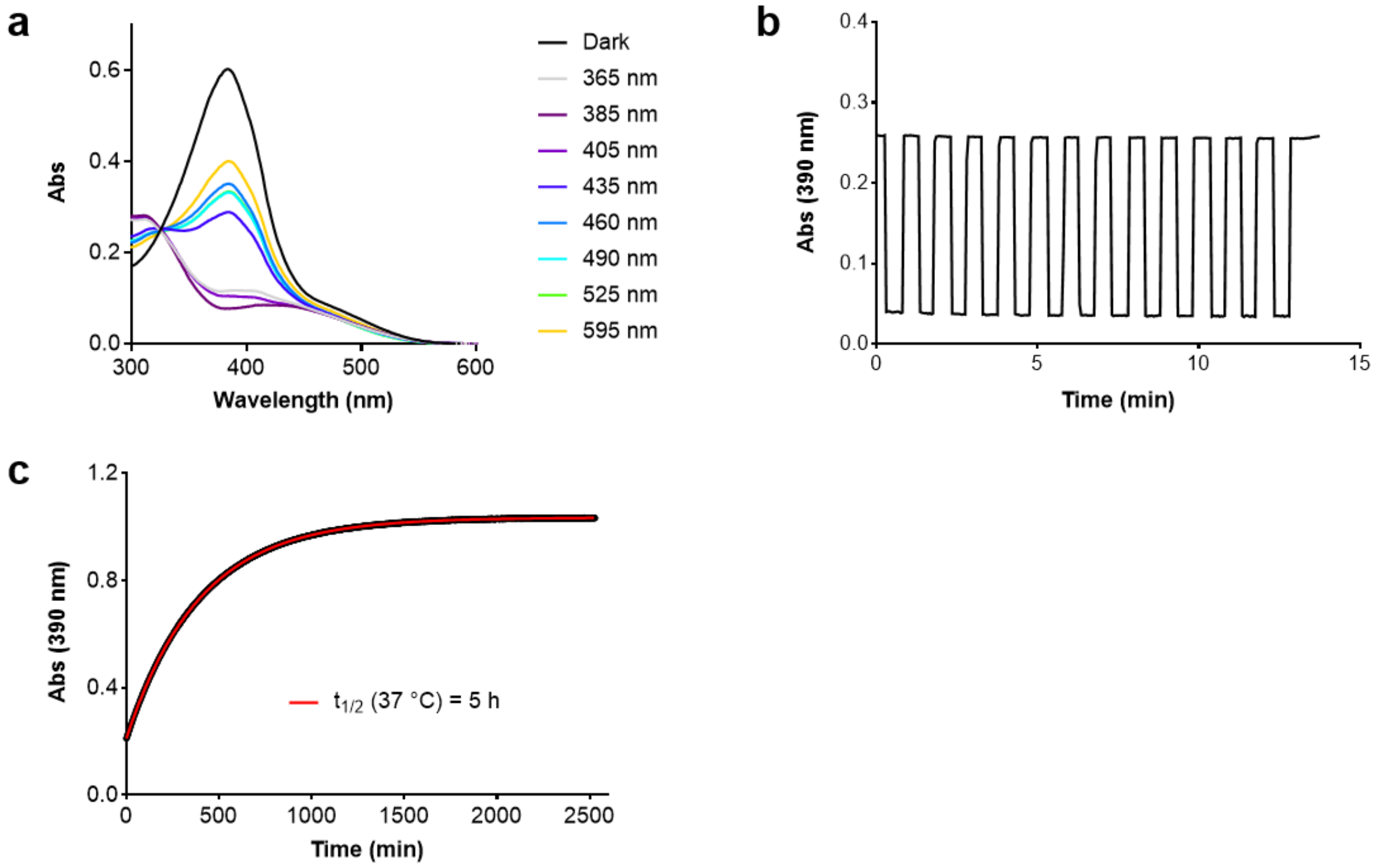

Figure S4. Photophysical characteristics of slow-relaxing **5**. (a) UV-Vis absorbance spectra of **5** in DMSO measured during irradiation at the indicated wavelengths after having reached the corresponding photostationary states. (b) Reversible photoswitching of **5** in DMSO between mostly-*E*- and mostly-*Z* PSSs, over multiple cycles, by alternating illuminations at 385 and 460 nm. (c) Thermal relaxation of ***Z*-5** in DMSO at 37 °C in the dark.

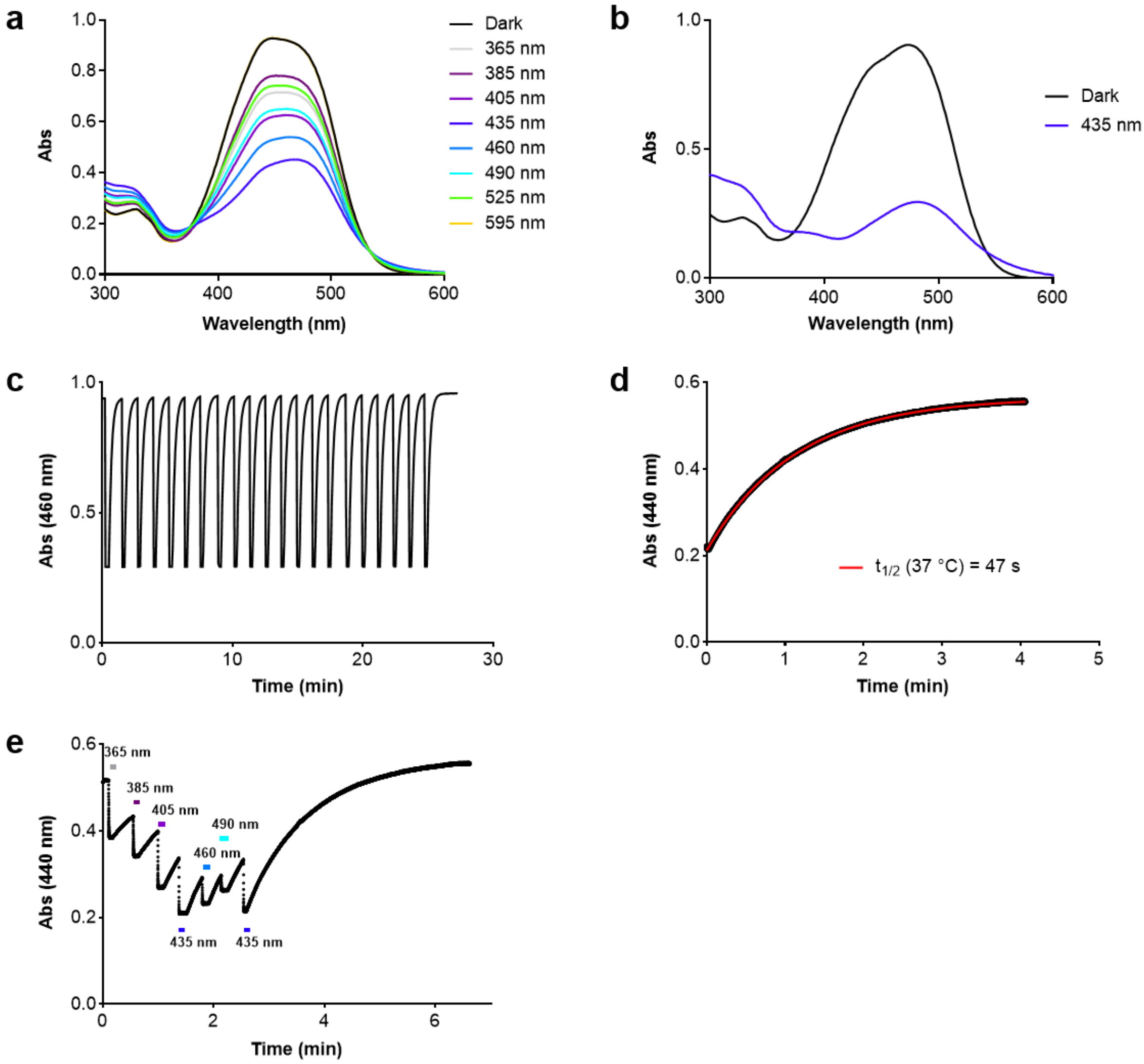

Figure S5. Photophysical characteristics of moderately-fast-relaxing **6**. (a) UV-Vis absorbance spectra of **6** in DMSO measured during irradiations at the indicated wavelengths after having reached the corresponding photostationary states. (b) UV-Vis absorbance spectra of **6** in DMSO:PBS 1:1 measured in the dark or during irradiation at 435 nm after having reached the corresponding photostationary state. (c) Reversible photoswitching of **6** in DMSO between mostly-*E*- and mostly-*Z* PSSs, over multiple cycles, by alternating illuminations at 435 and 595 nm. (d) Thermal relaxation of ***Z*-6** in DMSO:PBS 1:1 at 37 °C in the dark. (e) Absorbance at 440 nm of **6**, in DMSO:PBS 1:1, measured while being irradiated at the indicated wavelengths (due to the 10 mW/mm^2 light guide exit power densities, true photostationary states were probably only established for 405 nm and 435 nm due to the highest photoresponsivity at these wavelengths [examine the *y*-intervals between datapoints], whereas other wavelengths probably only approached their true photostationary states [though in the context of a photoswitchable tool, it is even more valuable, for biological applications to know the effective photostationary states under limited intensities, and since further work was conducted with 435 nm it was not of interest to examine the other wavelengths at different intensities]).

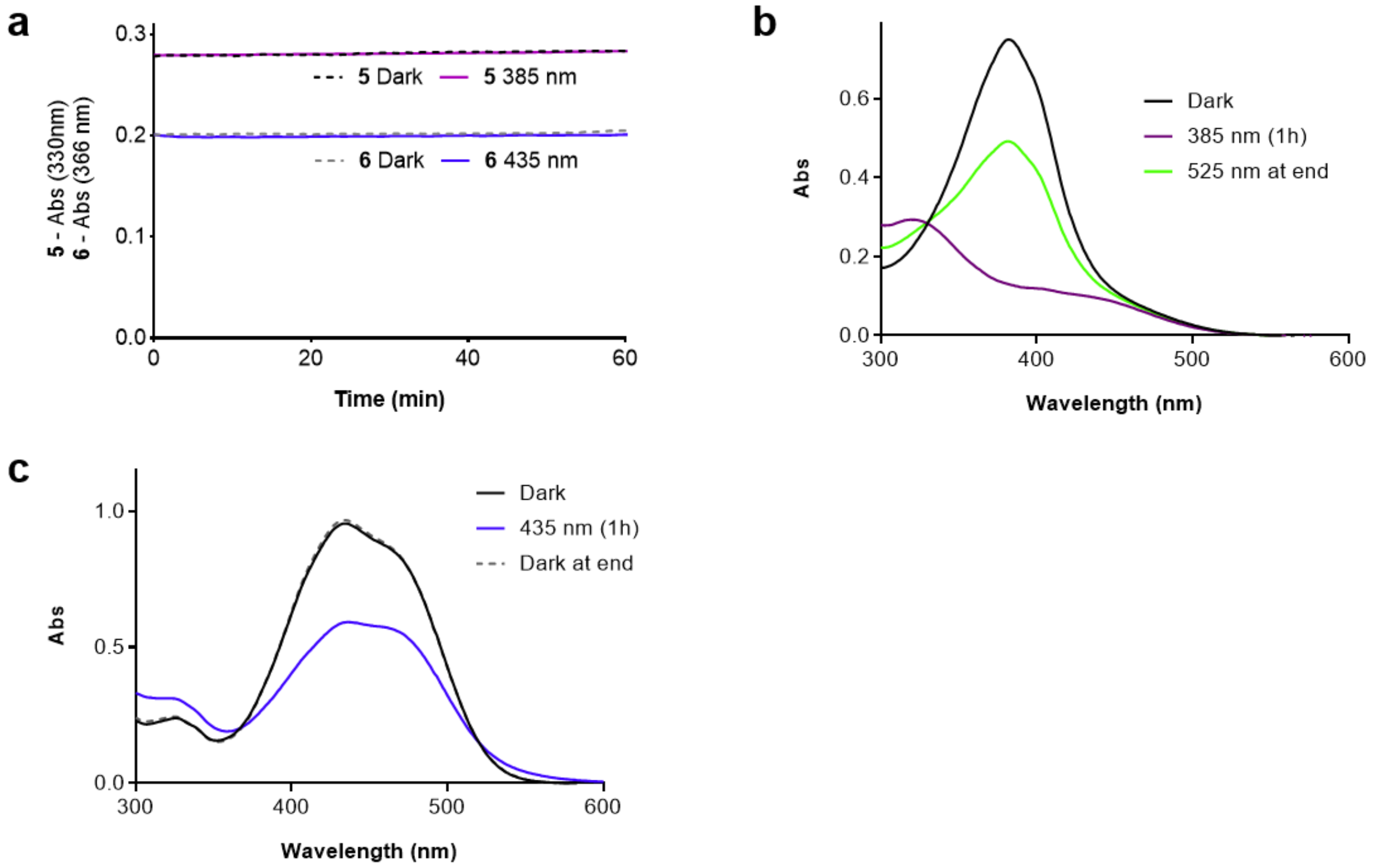

Figure S6. Stability of slow-relaxing **5** and moderately-fast-relaxing **6** towards GSH. (a) UV absorbance of all-*E,* and of 385/435 nm PSS states of **5**/**6**, measured at their respective *E/Z*-isosbestic points in PBS (pH 7.5):isopropanol 1:1, containing 2 mM reduced glutathione (GSH), measured at 37 °C. (b) UV-Vis absorbance spectra of **5** measured in PBS (pH 7.5):isopropanol 1:1, containing 2 mM glutathione at 37 °C in the dark, after 1 h of irradiation at 385 nm and after subsequent irradiation to the 525 nm photostationary state. (c) UV-Vis absorbance spectra of **6** measured in PBS (pH 7.5):isopropanol 1:1, containing 2 mM glutathione at 37 °C in the dark, after 1 h of irradiation at 435 nm and after subsequent thermal relaxation. Taken together, the kinetic stability of all four traces in Fig S6a, the match of the PSS spectra in the presence of GSH in Fig S6b with those in the absence of GSH (Fig S5a), and the full spectral reversibility in Fig S6c, indicate that there is no significant dark or photochemical GSH-induced degradation of either compound in *E* or *Z* isomer states.

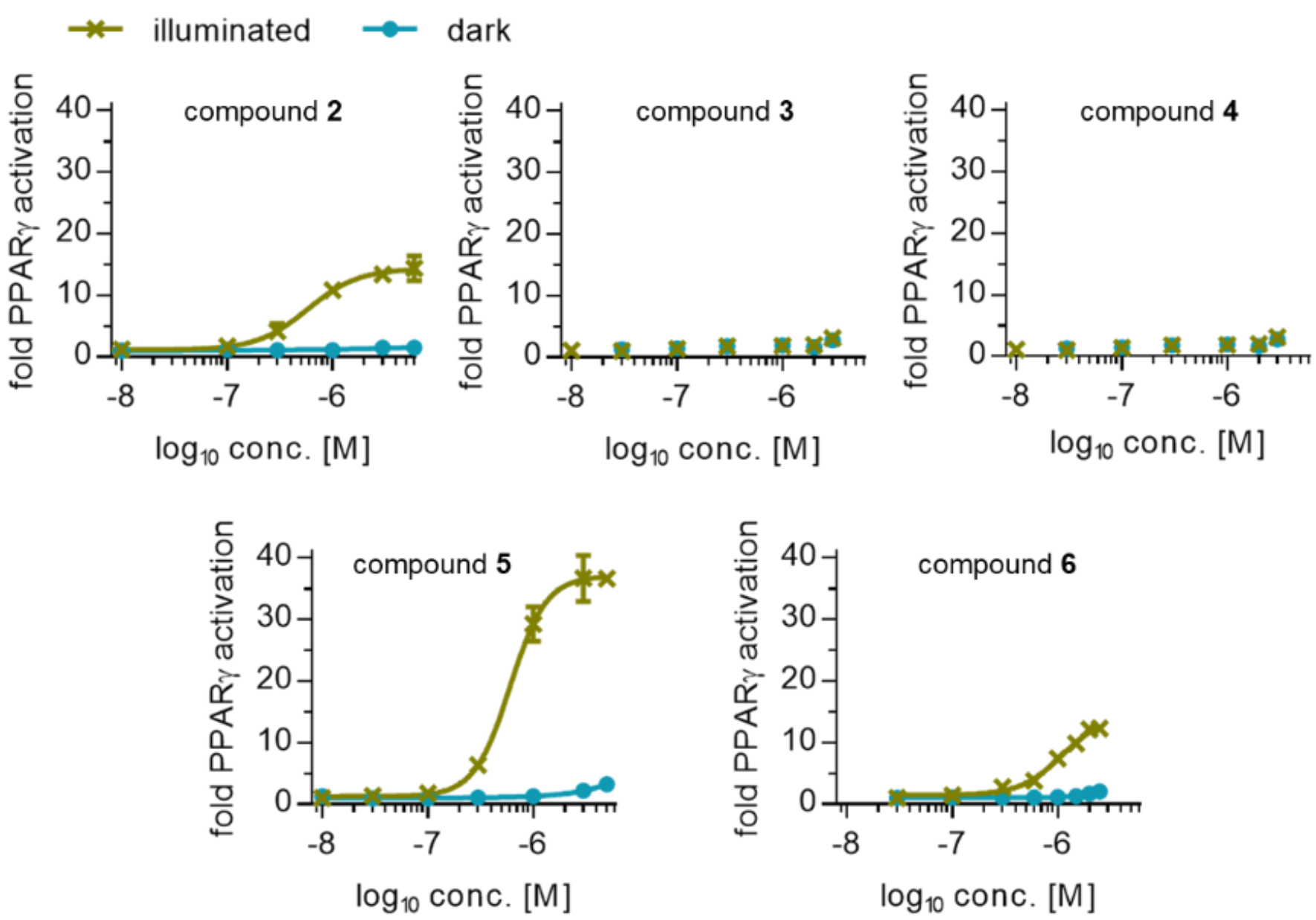

Figure S7. Dose-response curves of **2**-**6** in the Gal4-PPARγ reporter gene assay under dark and illuminated conditions [illuminations: 100 ms LEDs ON per 10 s LEDs OFF, using LEDs at 370 nm (**2**, **4**, **5**), 425 nm (**3**), and/or 435 nm (**6**)]. Data are the mean±S.E.M., n≥4.

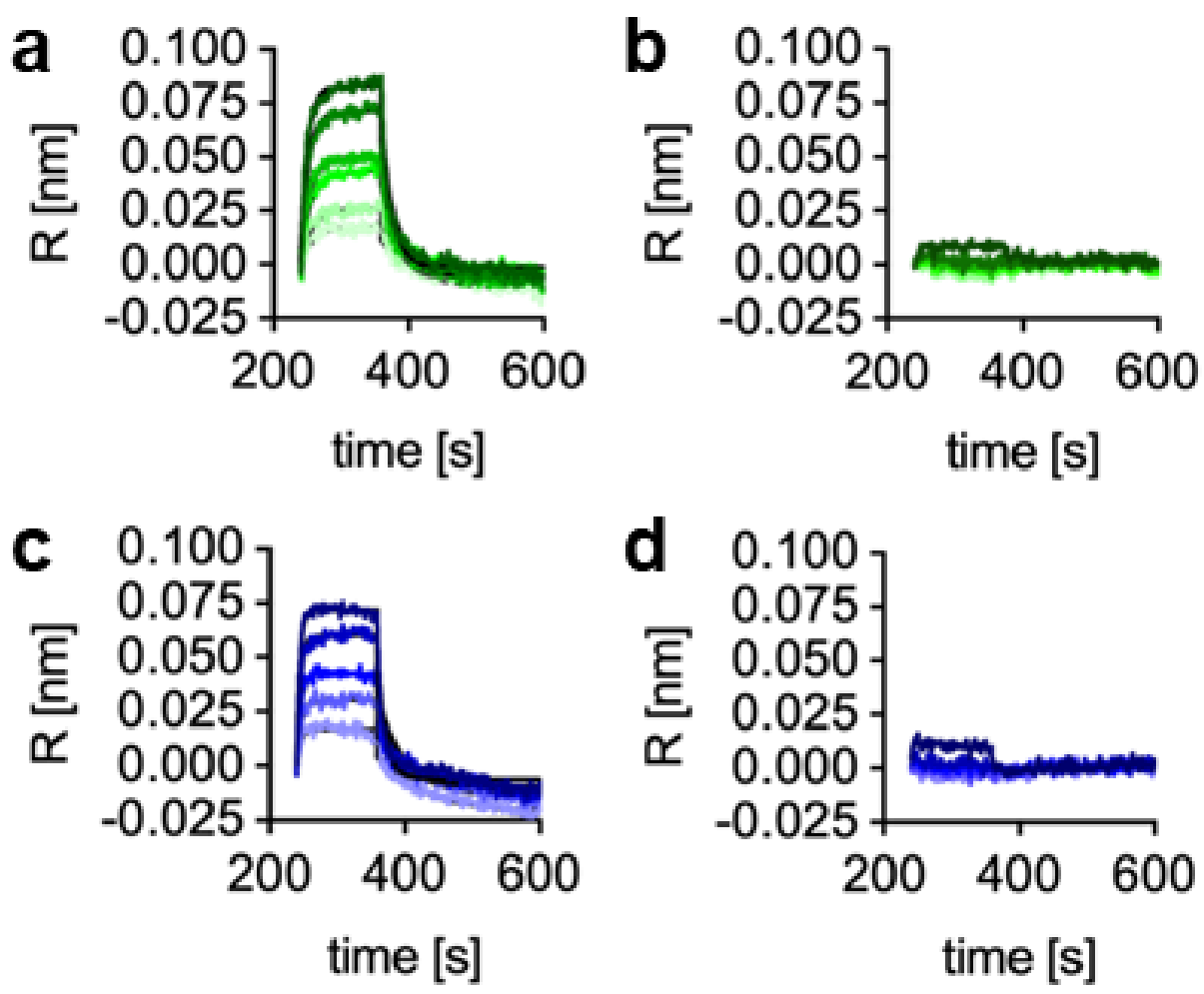

Figure S8. Evaluation of ligand binding to PPARγ by biolayer interferometry (BLI). (a) Light-activated *Z*-**2** (concentration range (light to dark color): 0.8-2.4 µM). (b) Dark-adapted *E*-**2** (concentration range: 0.4-6.5 µM). (c) Light-activated *Z*-**5** (concentration range: 1.2-8.0 µM). (d) Dark-adapted *E*-**5** (concentration range: 0.4-6.5 µM). All experiments were repeated in at least two independent replicates; one representative replicate is shown.

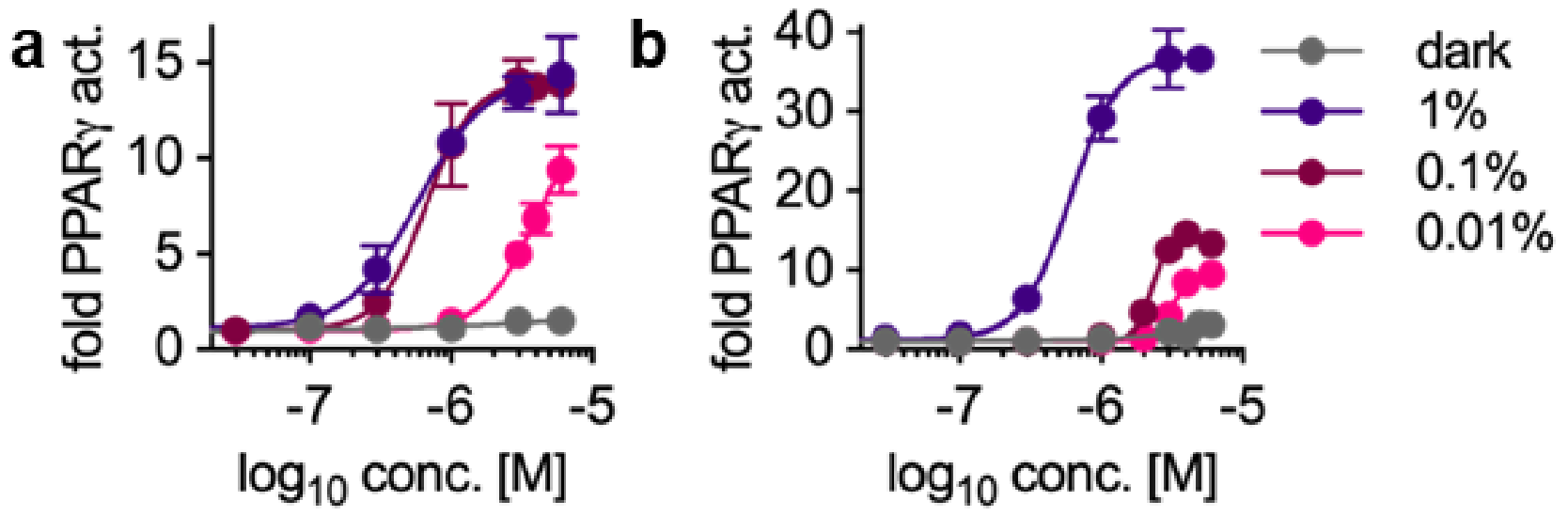

c

	PPARγ (EC_{50}, [max. act.])	
duty cycle	**2**	**5**
1%	0.50±0.09 μM [13±1-fold]	0.60±0.06 μM [38±1-fold]
0.1%	0.7±0.1 μM [13±1-fold]	2.3±0.1 μM [14±1-fold]
0.01%	4±1 μM [12±5-fold]	3.2±0.1 μM [10±1-fold]
0% (dark)	> 10 μM	> 10 μM

Figure S9. Impact of photon "intensity dosing" on PPARγ agonism of the slow-relaxing *Z*-agonists **2** and **5**, in the Gal4-PPARγ reporter gene assay, when very low duty cycles are applied (i.e., true wavelength-dependent PSS is not maintained during the assay, and lower-than-expected *Z* isomer fraction is applied). (a,b) Dose-response curves of (a) **2** and (b) **5**, in the dark and under illuminations at increasing intensity (duty cycle = time percentage where LEDs are ON, rather than OFF). (c) EC_{50} and maximum PPARγ activation values. Data are mean±S.E.M.; n≥4. The somewhat slower-relaxing ***Z*-2** delivered reproducible PPAR agonism from ≥0.1% duty cycle, whereas the apparent EC_{50} values of **5** continue to evolve up to 1% duty cycle (roughly the maximum tolerated duty cycle for long term illumination in live cells at these wavelength and applied intensity), which prevents reliable "light intensity-dosing" of PPARγ activation.

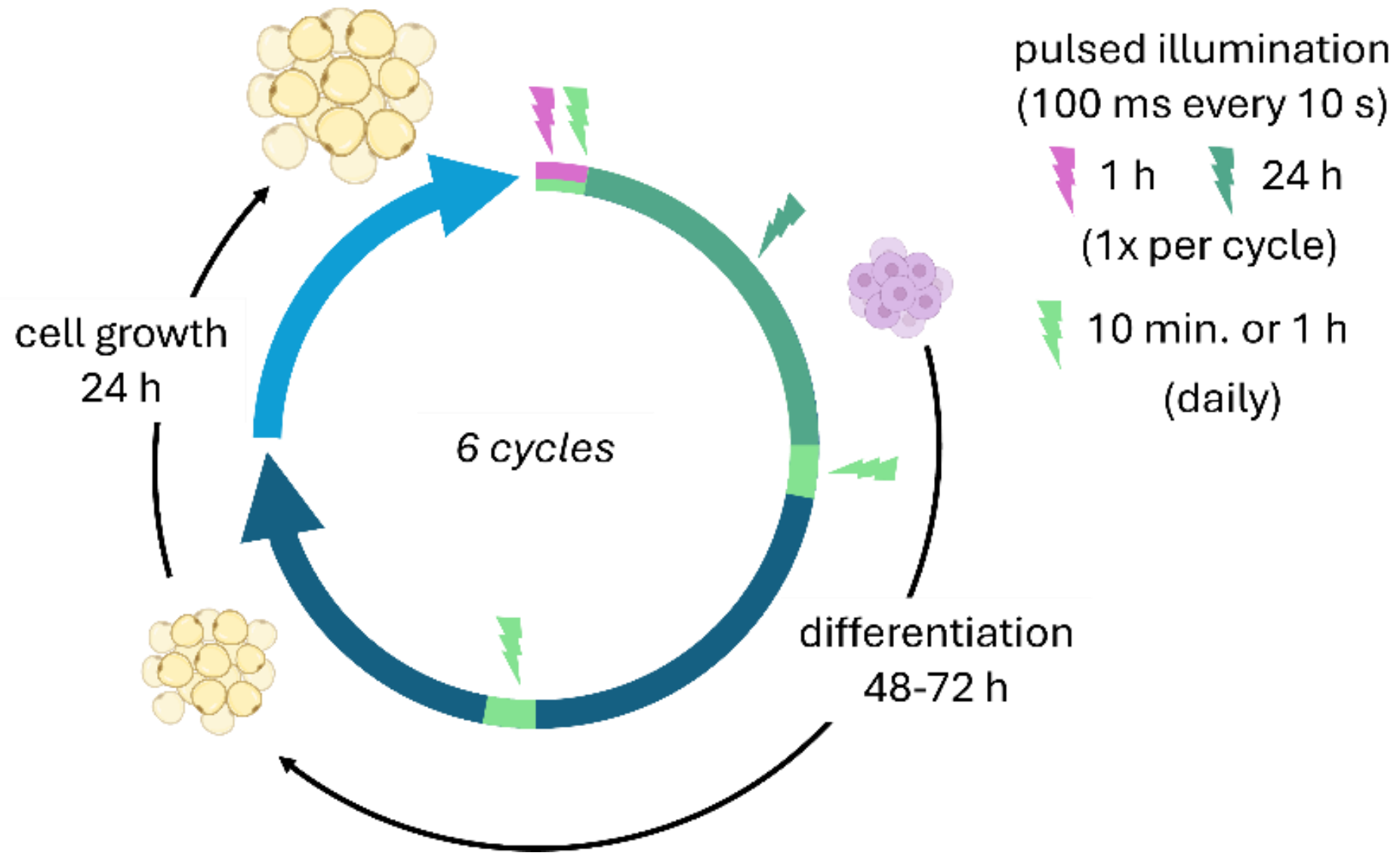

Figure S10. Schematic illustration of the adipocyte differentiation model. Adipocyte-derived stem cells (ASCs) were treated with insulin, isobutylmethylxanthine, dexamethasone and the light-activated PPARγ agonist **6** in six cycles with different illumination periods and timing using pulsed illumination with a CellDisco. The cells/samples were illuminated (i) 1 h on day 1 of each cycle, (ii) 24 h on day 1 of each cycle, (iii) 10 min. daily, or (iv) 1 h daily.

Methods

UV-Vis Spectroscopy. UV-Vis spectroscopy studies of photoswitching (before, during, and/or after orthogonal illuminations) and of *Z*-isomer relaxation rates were performed as previously described[1]. In brief, absorption spectra were acquired on a Varian CaryScan 50 in quartz glass cuvettes (*d*=1 cm) at ca. 10-50 µM. Illuminations for photoisomerization were performed with a pE-4000 LED engine (CoolLED; no additional optical filters were used, so wavelengths cited correspond to unfiltered LED emission spectra with the indicated emission centre wavelengths, detailed in reference[2]) by coupling it to a 5 mm diameter liquid light guide whose exit was fixed 8±3 mm above the solution surface, with all LED intensities adjusted to deliver 10 mW/mm^2 at light guide exit. 1 cm total height of solution [A_{max} ≤ 1 in the vertical optical path] and ca. 5 mm height to optical window were used, to minimise inner filter effects during illumination of solutions from above. Mixtures of DMSO with PBS (pH 7.5) were used as the solvent where solubility permitted (no aggregation detectable by scattering, or concentration-dependent absorption spectra profiles, or decrease of absorbance over time or during switching); otherwise, 100% DMSO was used, to prevent these complications as well as preventing effects of aggregation on the absorption and isomerization of the compounds (for example, in Fig. S1, at the mid-micromolar concentrations required for UV-Vis, **2** tends to aggregate from DMSO:PBS solutions, which would falsify photostationary state (PSS) and relaxation rate measurements: therefore pure-DMSO studies are shown). Thermal *Z*-isomer relaxation rates were determined at 37±1 °C; other studies were performed at room temperature (22±2 °C).

Glutathione Reactivity Assay. Glutathione (GSH) stability of **5** and **6** was determined by UV-Vis Spectroscopy. Compounds were dissolved in a freshly prepared 1:1 mixture of PBS (pH 7.5) and isopropanol containing 2 mM glutathione and incubated at 37 °C either in the dark or under constant irradiation using a pE-4000 illumination system (CoolLED) set to 2 mW/mm^2 at light guide exit for all wavelengths. After irradiation, **5** was switched back to the 525 nm PSS and **6** was allowed to relax in the dark. Absorbance was measured at 330 nm (**5**) or 366 nm (**6**) to observe degradation of the test compounds respectively.

Gal4-Hybrid Reporter Gene Assay. PPARγ modulation was determined in Gal4-hybrid reporter gene assays in HEK293T cells (German Collection of Microorganisms and Cell Culture GmbH, DSMZ) using pFR-Luc (Stratagene, La Jolla, CA, USA; reporter), pRL-SV40 (Promega, Madison, WI, USA; internal control) and pFA-CMV-PPARγ-LBD[3], coding for the hinge region and ligand binding domain of the canonical isoform of PPARγ. HEK293T cells were cultured in Dulbecco's modified Eagle's medium (DMEM), high glucose supplemented with 10% fetal calf serum (FCS), sodium pyruvate (1 mM), penicillin (100 U/mL), and streptomycin (100 µg/mL) at 37 °C and 5% CO2 and seeded in 96-well plates (3×10^4 cells/well). After 24 h, medium was changed to Opti-MEM without supplements and cells were transiently transfected using Lipofectamine LTX reagent (Invitrogen, Carlsbad, CA, USA) according to the manufacturer's protocol. Five hours after transfection, cells were incubated with the test compounds in Opti-MEM supplemented with penicillin (100 U/mL), streptomycin (100 µg/mL) and 0.1% DMSO in presence or absence of light. For illumination during incubation, cells were irradiated with light of the indicated wavelength using a CellDisco[4] containing 24 or 96 5mm LEDs (LEDS were supplied by Roithner Lasertechnik), pulsed at 100 ms "on" every 5, 10, 15 or 20 seconds. After 15 h, luciferase activity was measured using the Dual-Glo Luciferase Assay System (Promega) according to the manufacturer's protocol on a Tecan Spark luminometer (Tecan Deutschland GmbH, Crailsheim, Germany). Firefly luminescence was divided by Renilla luminescence and multiplied by 1000 resulting in relative light units (RLU) to normalize for transfection efficiency and cell growth. Fold activation was obtained by dividing the mean RLU of test compound by the mean RLU of the untreated control and relative activation was calculated by dividing the fold activation of a test sample by the fold activation of the reference agonist pioglitazone (1 µM). All samples were tested in at least three

biologically independent experiments in duplicates. For dose−response curve fitting and calculation of EC_{50} values and PPARγ modulation, the equation "[agonist] vs. response−variable slope (four parameters)" was used in GraphPad Prism (version 11.0.0, GraphPad Software, La Jolla, CA, USA). Selectivity profiling was performed according to the same protocol using the following Gal4-NR plasmids and reference agonists: pFA-CMV-THRα-LBD[3] (1 µM T3), pFA-CMV-RARα-LBD[3] (1 µM tretinoin), pFA-CMV-PPARα-LBD[3] (1 µM GW7647), pFA-CMV-PPARδ-LBD[3] (1 µM L165,041), pFA-CMV-RORγ-LBD[3] (1 µM T0901317), pFA-CMV-VDR-LBD[3] (1 µM calcitriol), pFA-CMV-CAR-LBD[3] (3 µM CITCO), pFA-CMV-LXRα-LBD[3] (1 µM T0901317), pFA-CMV-FXR-LBD[3] (1 µM GW4064), pFA-CMV-RXRα-LBD[3] (1 µM bexarotene), pFA-CMV-AR-LBD (1 µM BMS564929), pFA-CMV-Nur77-LBD[3] (1 µM compound 29 from Vietor et al. [5]).

Adipogenesis. *Cell culture and treatment:* Differentiation experiments of ASC52telo, hTERT cells (ATCC® SCRC-4000™) were conducted according to a previously described procedure[6]. In brief, cells were grown in DMEM high glucose, supplemented with 10% fetal calf serum, sodium pyruvate (1 mM), penicillin (100 U/mL), and streptomycin (100 µg/mL) at 37 °C and 5% CO_2. Cells were seeded in standard culture medium at a density of 5,000 cells per well in 96-well plates. After adherence overnight, cells were incubated with differentiation medium, composed of standard culture medium supplemented with human insulin (10 µg/mL, #I3536, Merck KgaA, Darmstadt, Germany), dexamethasone (1 µM, #D4902, Merck KgaA, Darmstadt, Germany,), isobutylmethylxanthine (0.5 mM, #I5879, Merck KgaA, Darmstadt, Germany), DMSO (final concentration 0.1%) and the respective test compounds. The cells were kept alternately for 48 h or 72 h in differentiation medium followed by a 24 h incubation with standard culture medium supplemented with 10 µg/mL insulin only. For illumination during incubation, cells were irradiated with light at a wavelength of 370 nm using a CellDisco[4] containing 24 5mm LEDs (LEDS were supplied by Roithner Lasertechnik), pulsed at 100 ms "on" every 10 seconds. A total of six cycle treatments were performed, accounting for a total differentiation time of 21 days. The indicated test compounds at 0.1% DMSO or 0.1% DMSO alone as vehicle control were supplemented freshly with every medium exchange. *Oil Red O staining:* After the 21-day differentiation procedure and test compound treatment, cells were washed with phosphate buffered saline (PBS) once and fixed with formalin (10%, stabilized with methanol, 100 µL per well, #15071, Morphisto GmbH, Offenbach am Main, Germany) for 15 min at room temperature. The fixing solution was aspirated, and the fixed cells were washed twice with 50% ethanol solution with the second wash step incubating for 15 min at room temperature to equilibrate the specimens for staining. Oil Red O (#O0625, Merck KgaA, Darmstadt, Germany) was prepared at 0.5% (w/v) in 50% ethanol (v/v) and 5% salicylic acid (w/v), filtered with grade 595 Whatman® filter paper (#311611, Schleicher & Schuell GmbH, London, UK) and a 0.2 µm syringe filter (FP 30/0,2 CA-S, #10462200, Schleicher & Schuell GmbH, London, UK). Upon equilibration, specimens were incubated with 100 µL of 0.5% Oil Red O solution for 45 min at room temperature before the staining solution was aspirated and the wells were washed once with 50% (v/v) ethanol solution and three times with ddH_2O. Specimens were kept in ddH_2O for subsequent analysis. For each well, two pictures were taken at a 4X magnification using a Motic®AE31E inverted microscope and a Moticam 1080 (Motic Hong Kong Ldt.). Images were corrected for skewed illumination, Oil Red O crystal precipitates were identified and removed, and the resulting processed pictures were dichromized to extract greyvalues from the red channel using CellProfiler (Version 4.2.6). An appropriate greyvalue threshold was selected for the dichromized binary pictures and quantified using ImageJ 1.54f. Each sample was tested in three biologically independent experiments (n=3).

Evaluation of PPARγ-Regulated Gene Expression in HT29 Cells. *Cell treatment:* HT29 human cells from colon adenocarcinoma (American Type Culture Collection, ATCC) were cultured in McCoy's 5A medium (Gibco, Thermo Fisher Scientific, Waltham, MA, USA) supplemented with 10% FCS,

penicillin (100 U/mL), and streptomycin (100 µg/mL) at 37 °C and 5% CO2 and seeded in 12-well plates (2.5 × 10^5 cells/well). After 24 h the medium was changed to McCoy's 5A medium supplemented with 0.1% FCS, penicillin (100 U/mL), and streptomycin (100 µg/mL) containing compound **6** (1 µM) solubilized with 0.1% DMSO or 0.1% DMSO alone as vehicle control. Cells were incubated for 24 h in the dark or under illumination at 435 nm using a CellDisco[4] containing 96 5 mm LEDs (Roithner Lasertechnik), pulsed at 100 ms "on" every 30 (0.33%), or 15 (0.66%) seconds. After 24 h incubation, the medium was removed, cells were washed with PBS once and harvested using trypsin/EDTA. The resulting cell suspensions were diluted using complete growth medium and centrifuged for 5 min at 300 g. The supernatant was fully aspirated, and the cell pellet was immediately frozen at −80 °C until further processing. *RNA extraction and linearization:* Total RNA was isolated using the peqGOLD Total RNA Kit (VWR International, Darmstadt, Germany) following the manufacturer's instructions. RNA concentration and purity were assessed using a NanoDrop One UV−vis spectrophotometer (Thermo Fisher Scientific) at 260/280 nm. Right before reverse transcription (RT), RNA was linearized at a concentration of 110 ng/µL at 65 °C for 10 min and then immediately incubated on ice for at least 1 min. *Reverse transcription and quantitative polymerase chain reaction (qPCR):* Reverse transcription was performed using 2 µg of total RNA, 20 U Recombinant RNasin Ribonuclease Inhibitor (Promega, Mannheim, Germany), 100 U SuperScript IV Reverse Transcriptase including 5× First Strand Buffer and 0.1 M dithiothreitol (Thermo Fisher Scientific), 3.75 ng of linear acrylamide, 625 ng of random hexamer primers (Merck, Darmstadt, Germany), and 11.25 nmol of deoxynucleoside triphosphate mix (2.8 nmol each ATP, TTP, CTP, GTP; Thermo Fisher Scientific) at a volume of 22.45 µL at 50 °C for 10 min and 80 °C for 10 min using a Thermal cycler XT96 (VWR International). A qPCR was conducted on an Applied Biosystems™ QuantStudio 1 (Waltham, USA) and a qTOWER^3 (Analytik Jena, Jena, Germany) using a SYBR green-based detection method. 0.2 µL of prepared cDNA was added to 6 pmol of forward and reverse primer, respectively, 0.8 U Taq DNA Polymerase (New England Biolabs, Ipswich, MA, USA), 40 ppm of SYBR Green I (Sigma-Aldrich), 15 nmol of deoxynucleoside triphosphate mix (as indicated above), 60 nmol of $MgCl_2$, 4 µg of bovine serum albumin (Thermo Fisher Scientific), 20% BioStab PCR Optimizer II (Merck, Darmstadt, Germany), and 10% Taq buffer without detergents (Thermo Fisher Scientific), topped up to a final volume of 20 µL with ddH_2O. Samples underwent 40 cycles of 15 s denaturation at 95 °C, 15 s of primer annealing at primer-specific temperatures and 20 s of elongation at 68 °C. PCR product specificity was evaluated using a melting curve analysis ranging from 65 to 95 °C. The following primers and annealing temperatures were used: hGAPDH (60.0 °C): 5′- AGG TCG GAG TCA ACG GAT TT -3′ (fwd), 5′- TTC CCG TTC TCA GCC TTG AC -3′ (rev); hFABP1 (60.0 °C): 5′- GGG AGC TCT ATT GCC ACC AT -3′ (fwd), 5′- GGC AGA CCG ATT GCC TTC AT -3′ (rev); hKLF4 (60 °C): 5′- AAG CCA AAG AGG GGA AGA CG -3′ (fwd), 5′- CAT GTG TAA GGC GAG GTG GT -3′ (rev).

Bio-layer interferometry (BLI). *Protein expression, biotinylation, and purification:* The PPARγ ligand-binding domain (LBD; residues 234–505, UniProt accession P37231), fused to an N-terminal His-tag, TEV protease cleavage site, and AviTag, was cloned into the pET29BH4 vector and expressed in E. coli Rosetta2 (BL21). An overnight starter culture grown at 30 °C was used to inoculate a total of 2.4 L of Terrific Broth supplemented with kanamycin and chloramphenicol. Protein expression was induced at an OD600 of ~1.2 with 0.5 mM IPTG and continued for 12 h at 18 °C. Cells were harvested by centrifugation at 8,000 × g. The protein was purified by immobilized metal affinity chromatography (IMAC), followed by overnight TEV protease cleavage during dialysis. Cleaved PPARγ was separated from uncleaved protein, the His-tag, and His-tagged TEV protease by passage over a HisTrap column (Cytiva) on an ÄKTA chromatography system. Endogenous lipids were removed by two consecutive 2 h incubations with Lipidex-5000 resin. The protein was then biotinylated overnight at 4 °C using BirA ligase in the presence of 10 mM $MgSO_4$, 3 mM ATP, and 0.8 mM biotin. Final purification was performed by size-exclusion chromatography on an ÄKTA system using a HiLoad Superdex 200 16/60 column equilibrated with 20 mM HEPES (pH 7.4) and 200 mM NaCl. *BLI Measurements:* BLI

experiments were performed on an Octet R8 instrument (Sartorius) using superstreptavidin (SSA) biosensors. Biotinylated PPARγ was immobilized at 10 μg/mL for 10 min, reaching a loading response of ~6 nm. Measurements were carried out in buffer containing 20 mM HEPES (pH 7.4), 150 mM NaCl, 0.5 mg/mL BSA, 0.05% Tween-20, and 1% DMSO. Dose–response series comprised three-point (0.75–1.5 μM, compound **1**) or five-point dilution ranges (0.8–2.4 μM for ***Z*-2**; 1.2–8.0 μM for ***Z*-5**; 0.4–6.5 μM for *E*-**2** and *E*-**5**). Each experiment was performed at least in duplicate. Association of compounds was recorded for 120 s, followed by a 240 s dissociation phase. Non-specific binding and baseline drift were corrected using two controls: protein-loaded sensors without compound and unloaded sensors exposed to compound at matching concentrations. Effective ligand concentrations used for fitting were adjusted based on non-specific binding. Kinetic parameters (K_d, k_{on}, k_{off}) were determined by local fitting of association and dissociation phases using ForteBio Data Analysis software. For each replicate, parameters were first summarized as median values across the concentration series, and final values were obtained by averaging replicate medians. Data are presented as mean ± SD.

Synthesis and analytical characterization of **1-6** and precursors

General. All chemicals were of reagent grade, purchased from commercial sources (e.g., Sigma-Aldrich, TCI, BLDpharm) and used without further purification. All reactions were conducted in absolute solvents (Thermo Fisher Scientific). Other solvents, especially for workup procedures, were of reagent grade or purified by distillation (cyclohexane, EtOAc, EtOH). Reactions were monitored and R_f-Values were determined by thin layer chromatography (TLC) using fluorescent dye-coated silica gel 60 on aluminum sheets by Merck and visualized under ultraviolet light (254 nm). Purification by flash chromatography was carried out on an Interchim puriFlash® XS520Plus system (Advion, Ithaca, NY, USA) using high performance spherical silica columns (SIHP, 50 µm) by Interchim and a gradient of cyclohexane to EtOAc. Dry column vacuum chromatography was performed following literature procedure[7] using silica gel (0.060-0.200 nm, 60Å; Thermo Fisher Scientific) and a gradient of cyclohexane to EtOAc with 2% HOAc added. Reverse phase chromatography was performed on a puriFlash® 5.250 system (Advion) using C18HP columns (SIHP, 15 µM) by Interchim and a gradient of water with 20% acetonitrile to 100% acetonitrile (HPLC gradient grade). Preparative HPLC was performed in the same manner using a US5C18HQ-150/300 PREP-LC column by Interchim. Melting points (m.p.) were determined with a Krüss M3000 melting point meter (A. Krüss Optronic GmbH, Hamburg, Germany) and are uncorrected. HRMS were obtained with a Thermo Scientific™ Orbitrap Exploris™ 120 mass spectrometer using electrospray ionization (ESI). NMR spectra were recorded on Bruker Avance III HD 400 MHz or 500 MHz spectrometers equipped with a CryoProbe™ Prodigy broadband probe (Bruker). Chemical shifts (δ) are reported in ppm relative to residual protium signals in the NMR solvent (^{1}H-NMR: $(CD_3)_2CO$: δ = 2.05 ppm; $(CD_3)_2SO$: δ = 2.50 ppm; $CDCl_3$: δ = 7.26 ppm, ^{13}C-NMR: $(CD_3)_2CO$: δ = 206.26, 29.84 ppm; $(CD_3)_2SO$: δ = 39.52 ppm; $CDCl_3$: δ = 77.16 ppm), and coupling constants (*J*) in hertz (Hz). IR spectra were recorded on a Shimadzu (Kyōto, Japan) fourier transform infrared spectrophotometer (IRSpirit™-TX) in transmission mode at a resolution of 2 cm^{-1}. All compounds for biological testing had a purity >95% according to HPLC (detection at 254, 280 and 320 nm). HPLC analyses were performed using a Shimadzu (Kyōto, Japan) HPLC system (CBM-40 controller, LC-40D quaternary pump, SIL-20A-HT autosampler, SPD-M40 photodiode array detector, LCMS-2020 mass spectrometer with ESI ion source). As stationary phase a Zorbax SBAq column (3 × 100 mm, 3.5 µm, Agilent, protected with a 0.5 µm and a 0.2 µm frit) was used in combination with water (+0.1% FA) (A) and acetonitrile (+0.1% FA) (B) as mobile phase at a flow rate of 0.5 mL/min. The following method was applied: A:B = 35:65, 3 min isocratic, followed by 7 min linear gradient to 100% B and B 100%, 2 min isocratic; A and B as above. MS detection was carried out in positive and negative scan mode (*m/z* range 300–600) and in positive and negative single ion mode using the respective $[(M + H)]^+$ and $[(M - H)]^-$ as target *m/z*.

2-((6-((2,4-Dichlorophenyl)sulfonamido)benzo[*d*]thiazol-2-yl)thio)octanoic acid (1) The compound was synthesized following literature procedure[8]. R_f = 0.31 (cyclohexane/EtOAc 1:1 + 0.25% FA). ^{1}H-NMR (500 MHz, $(CD_3)_2SO$): δ (ppm) = 13.13 (s, 1H), 10.91 (s, 1H), 8.02 (d, *J* = 8.5 Hz, 1H), 7.85 (d, *J* = 2.1 Hz, 1H), 7.75 – 7.70 (m, 2H), 7.58 (dd, *J* = 8.5, 2.1 Hz, 1H), 7.19 (dd, *J* = 8.8, 2.2 Hz, 1H), 4.45 (t, *J* = 7.0 Hz, 1H), 1.91 (ddt, *J* = 31.9, 14.3, 7.1 Hz, 2H), 1.39 (p, *J* = 7.3 Hz, 2H), 1.33 – 1.16 (m, 6H), 0.82 (t, *J* = 6.8 Hz, 3H).^{13}C-NMR (126 MHz, $(CD_3)_2SO$): δ (ppm) = 171.7, 164.1, 149.4, 138.7, 135.9, 135.4, 133.6, 133.0, 132.0, 131.4, 128.0, 121.8, 119.5, 112.7, 50.2, 31.5, 30.9, 28.0, 26.3, 21.9, 13.9. HRMS (ESI): *m/z* calculated 531.0046 for $C_{21}H_{21}Cl_2N_2O_4S_3$, found 531.0044 ($[M-H]^-$).

(*E*)-2-((6-((2,4-Dichlorophenyl)diazenyl)benzo[*d*]thiazol-2-yl)thio)octanoic acid (*E*-2). 2,4-dichloroaniline (**10**, 65 mg, 0.40 mmol, 2.0 equiv.) was dissolved in 2 mL of DCM, Oxone® (0.25 g, 0.80 mmol, 4.0 equiv.) in 2 mL distilled water was added to the solution. The resulting mixture was stirred for 2 h at room temperature. The reaction mixture was then diluted with DCM and washed with 1 M HCl, saturated $NaHCO_3$ and distilled water. The organic layer was dried over $MgSO_4$ and the

solvent was removed under reduced pressure to obtain the nitroso derivative as a brown oil that was used in the next step without further purification. The nitroso derivative and **9** (65 mg, 0.20 mmol, 1.0 equiv.) were suspended in 2 mL acetic acid and the reaction mixture was stirred for 60 h at room temperature. Then, the acetic acid was removed under reduced pressure and the crude product was purified by flash chromatography (SiO_2; gradient: 0-25% EtOAc in cyclohexane) obtaining *E*-**2** as an orange solid (45 mg, 47%). R_f = 0.27 (cyclohexane/EtOAc 7:3 + 0.25% FA). M.p.: 103.4-105.6°C. ^{1}H-NMR (400 MHz, $(CD_3)_2SO$): δ (ppm) = 8.67 (dd, *J* = 1.8, 0.7 Hz, 1H), 8.04 (d, *J* = 1.8 Hz, 1H), 8.03 (d, *J* = 0.7 Hz, 1H), 7.94 (d, *J* = 2.2 Hz, 1H), 7.74 (d, *J* = 8.7 Hz, 1H), 7.60 (dd, *J* = 8.7, 2.2 Hz, 1H), 4.62 (t, *J* = 6.9 Hz, 1H), 2.05 – 1.92 (m, 2H), 1.48 – 1.41 (m, 2H), 1.36 – 1.23 (m, 6H), 0.85 (t, *J* = 6.9 Hz, 3H).^{13}C-NMR (101 MHz, $(CD_3)_2SO$): δ (ppm) = 171.7, 169.6, 154.9, 148.6, 146.7, 136.6, 136.1, 135.0, 130.3, 128.5, 121.9, 121.1, 118.8, 118.5, 50.3, 31.5, 30.9, 28.1, 26.3, 22.0, 13.9. IR (KBr, cm^{-1}): $\tilde{\nu}$ = 3087 (w), 3069 (w), 2923 (s), 2855 (m), 1716 (vs), 1577 (s), 1401 (m), 1100 (m), 823 (s). HPLC purity > 95%. HRMS (ESI): *m/z* calculated 482.0530 for $C_{21}H_{22}Cl_2N_3O_2S_2$, found 482.0487 ($[M+H]^+$).

(*E*)-2-((6-((4-(Dimethylamino)-2-hydroxyphenyl)diazenyl)benzo[*d*]thiazol-2-yl)thio) octanoic acid (*E*-3). **9** (0.13 g, 0.40 mmol, 1.0 equiv.) was dissolved in a mixture of THF and distilled water (1:1, 4 mL). 0.4 mL of aqueous 2 M HCl (0.80 mmol, 2.0 equiv.) was added. The mixture was cooled to 0 °C and stirred for 10 min. Sodium nitrite (33 mg, 0.48 mmol, 1.2 equiv.) solved in 0.5 mL of distilled water was added dropwise. The mixture was stirred for 35 min at 0 °C. Next, sodium acetate (33 mg, 0.40 mmol, 1.0 equiv.) was added and the mixture was stirred for another 5 min. 3-(*N*,*N*-dimethylamino)phenol (55 mg, 0.40 mmol, 1.0 equiv.) solved in 0.5 mL THF was added dropwise. The reaction mixture was allowed to warm to ambient temperature and stirred overnight. THF was removed under reduced pressure and the aqueous residue was extracted with DCM (3 x 5 mL). The combined organic layers were dried over $MgSO_4$ and the solvent was removed under reduced pressure. The crude product was purified by dry column vacuum chromatography[7] (SiO_2; step gradient: 0%, 10%, 20%, 25%, 30%, 35%, 40%, 50%, 60% EtOAc in cyclohexane, both containing 2% HOAc). Afterward, the product was suspended in water/acetonitrile and subjected to lyophilization obtaining *E*-**3** as a red solid (60 mg, 32%). R_f = 0.34 (cyclohexane/EtOAc 1:1 + 0.25% FA). M.p.: 172.4-174.0°C. ^{1}H NMR (500 MHz, $(CD_3)_2SO$): δ (ppm) = 13.29 (s, 1H), 8.35 (d, *J* = 1.8 Hz, 1H), 7.89 – 7.81 (m, 2H), 7.58 (d, *J* = 9.1 Hz, 1H), 6.52 (dd, *J* = 9.3, 2.6 Hz, 1H), 6.13 (d, *J* = 2.7 Hz, 1H), 4.32 (s, 1H), 3.07 (s, 6H), 1.98 (q, *J* = 7.6 Hz, 2H), 1.52 – 1.45 (m, 1H), 1.43 – 1.35 (m, 1H), 1.28 – 1.19 (m, 6H), 0.82 (t, *J* = 6.8 Hz, 3H). ^{13}C NMR (126 MHz, $(CD_3)_2SO$): δ (ppm) = 171.9, 170.1, 156.8, 154.1, 153.2, 146.9, 135.7, 131.5, 130.2, 121.2, 119.7, 113.8, 106.0, 97.6, 56.1, 39.9 (2 x *C*), 33.5, 31.3, 28.6, 26.2, 22.1, 14.0. IR (KBr, cm^{-1}): $\tilde{\nu}$ = 3093 (w), 2925 (s), 2857 (m), 1741 (s), 1617 (vs), 1437 (m), 1134 (s). HPLC purity > 95%. HRMS (ESI): *m/z* calculated 473.1675 for $C_{23}H_{29}N_4O_3S_2$, found 473.1667 ($[M+H]^+$).

(*E*)-2-((6-((4-Chloro-2-hydroxyphenyl)diazenyl)benzo[*d*]thiazol-2-yl)thio)octanoic acid. (*E*-4). 2-((*tert*-butyldimethylsilyl)oxy)-4-chloroaniline (**13**, 0.16 g, 0.60 mmol, 2.0 equiv.) was cooled to 0 °C and a solution of *m*-CPBA (0.21 g, 1.2 mmol, 4.0 equiv.) in 4 mL of DCM was added. The resulting mixture was stirred for 1 h at 0 °C. Afterward, the reaction was quenched by adding sodium carbonate (0.13 g, 1.2 mmol, 4.0 equiv.) in 10 mL of distilled water and the mixture was allowed to warm to ambient temperature. The product was extracted with DCM (3 x 10 mL) and the combined organic layers were dried over $MgSO_4$. The solvent was removed under reduced pressure to obtain the nitroso derivative as a brown oil that was used in the next step without further purification. The nitroso derivative and **9** (97 mg, 0.30 mmol, 1.0 equiv.) were suspended in 3 mL acetic acid and the reaction mixture was stirred for 60 h at room temperature to obtain intermediate **12**. Then, 1.5 mL of water and 1.5 mL of trifluoroacetic acid were added and the mixture was stirred at room temperature for another 2 h. Afterward, the reaction was quenched with water (10 mL) and the product was extracted with EtOAc (3 x 10 mL). The combined organic layers were dried over $MgSO_4$ and the solvent was removed

under reduced pressure. The crude product was purified by dry column vacuum chromatography[7] (SiO_2; step gradient: 0%, 2%, 4%, 6%, 8%, 10%, 12% EtOAc in cyclohexane, both containing 2% HOAc) followed by recrystallization from acetonitrile/water obtaining *E*-**4** as orange crystals (62 mg, 44%). R_f = 0.47 (cyclohexane/EtOAc 1:1 + 0.25% FA). M.p.: 170.0-170.4°C. ^{1}H-NMR (400 MHz, $(CD_3)_2CO$): δ (ppm) = 8.63 (dd, *J* = 2.0, 0.5 Hz, 1H), 8.14 (dd, *J* = 8.8, 2.1 Hz, 1H), 8.01 (dd, *J* = 8.7, 0.5 Hz, 1H), 7.95 (d, *J* = 8.6 Hz, 1H), 7.15 (dd, *J* = 8.6, 2.2 Hz, 1H), 7.10 (d, *J* = 2.2 Hz, 1H), 4.78 (t, *J* = 7.0 Hz, 1H), 2.13 (td, *J* = 14.5, 7.0 Hz, 2H), 1.57 (p, *J* = 7.4 Hz, 2H), 1.42 (p, *J* = 6.7 Hz, 2H), 1.35 – 1.29 (m, 4H), 0.91 – 0.86 (m, 3H). ^{13}C-NMR (101 MHz, $(CD_3)_2CO$): δ (ppm) = 171.9, 169.9, 155.9, 155.0, 148.6, 139.3, 137.5, 137.4, 132.3, 122.9, 122.0, 121.5, 118.8, 117.3, 51.0, 32.9, 32.3, 29.5 (covered by solvent signal), 27.6, 23.2, 14.3. IR (KBr, cm^{-1}): $\tilde{\nu}$ = 3431 (m, br), 3095 (w), 3078 (w), 2925 (s), 2856 (m), 1718 (vs), 1608 (s), 1254 (s), 828 (s), 808 (s). HPLC purity > 95%. HRMS (ESI): *m/z* calculated 462.0718 for $C_{21}H_{21}ClN_3O_3S_2$, found 462.0715 ($[M-H]^-$).

(*E*)-2-((6-((4-Chloro-2-methoxyphenyl)diazenyl)benzo[*d*]thiazol-2-yl)thio)octanoic acid (*E*-5). 4-chloro-2-methoxyaniline (**11**, 65 mg, 0.40 mmol, 2.0 equiv.) was dissolved in 2 mL of DCM, Oxone® (0.25 g, 0.80 mmol, 4.0 equiv.) in 2 mL distilled water was added to the solution. The resulting mixture was stirred for 2 h at room temperature. The reaction mixture was then diluted with DCM and washed with 1 M HCl, saturated $NaHCO_3$ and distilled water. The organic layer was dried over $MgSO_4$ and the solvent was removed under reduced pressure to obtain the nitroso derivative as a brown oil that was used in the next step without further purification. The nitroso derivative and **9** (65 mg, 0.20 mmol, 1.0 equiv.) were suspended in 2 mL acetic acid and the reaction mixture was stirred for 60 h at room temperature. Then, the acetic acid was removed under reduced pressure and the crude product was purified by dry column vacuum chromatography[7] (SiO_2; step gradient: 0%, 5%, 10%, 15%, 20%, 25%, 30% EtOAc in cyclohexane, both containing 2% HOAc) followed by reverse phase HPLC (C18, 80-30% water in acetonitrile, both containing 0.1% FA) obtaining *E*-**5** as an orange solid (30 mg, 32%). R_f = 0.35 (cyclohexane/EtOAc 1:1 + 0.25% FA). M.p.: 146.2-149.8°C. ^{1}H-NMR (500 MHz, $(CD_3)_2SO$): δ (ppm) = 8.48 (d, *J* = 1.8 Hz, 1H), 7.97 – 7.89 (m, 2H), 7.59 (d, *J* = 8.6 Hz, 1H), 7.40 (d, *J* = 2.1 Hz, 1H), 7.12 (dd, *J* = 8.7, 2.1 Hz, 1H), 4.32 (t, *J* = 5.9 Hz, 1H), 4.00 (s, 3H), 1.98 (q, *J* = 7.3 Hz, 2H), 1.52 – 1.44 (m, 1H), 1.44 – 1.35 (m, 1H), 1.29 – 1.21 (m, 6H), 0.83 (t, *J* = 6.8 Hz, 3H). ^{13}C-NMR (126 MHz, $(CD_3)_2SO$): δ (ppm) = 172.5, 172.0, 157.3, 155.0, 148.7, 140.2, 137.3, 135.7, 121.3, 121.2, 120.8, 117.7, 117.0, 113.9, 56.6, 56.1, 33.5, 31.3, 28.7, 26.3, 22.1, 14.0. IR (KBr, cm^{-1}): $\tilde{\nu}$ = 3421 (s, br), 2927 (s), 2856 (m), 1586 (vs), 1404 (vs), 1247 (s). HPLC purity > 95%. HRMS (ESI): *m/z* calculated 476.0874 for $C_{22}H_{23}ClN_3O_3S_2$, found 476.0870 ($[M-H]^-$).

(*E*)-2-((6-((2-Dhloro-4-(dimethylamino)phenyl)diazenyl)benzo[*d*]thiazol-2-yl)thio) octanoic acid (*E*-6). **9** (97 mg, 0.30 mmol, 1.0 equiv.) was dissolved in a mixture of THF and distilled water (1:1, 3 mL). 0.3 mL of aqueous 2 M HCl (0.60 mmol, 2.0 equiv.) was added. The mixture was cooled to 0 °C and stirred for 10 min. Sodium nitrite (25 mg, 0.36 mmol, 1.2 equiv.) solved in 0.5 mL of distilled water was added dropwise. The mixture was stirred for 35 min at 0 °C. Next, sodium acetate (25 mg, 0.30 mmol, 1.0 equiv.) was added and the mixture was stirred for another 5 min. 3-Chloro-*N,N*-dimethylaniline (**15**, 44 µL, 0.30 mmol, 1.0 equiv.) solved in 0.5 mL THF was added dropwise. The reaction mixture was allowed to warm to ambient temperature and stirred overnight. THF was removed under reduced pressure and the aqueous residue was extracted with DCM (3 x 5 mL). The combined organic layers were dried over $MgSO_4$ and the solvent was removed under reduced pressure. The crude product was purified by dry column vacuum chromatography[7] (SiO_2; step gradient: 0%, 5%, 10%, 15%, 20%, 25%, 30% EtOAc in cyclohexane, both containing 2% HOAc). Afterward, a trituration with water/acetonitrile containing 0.1% FA followed, obtaining *E*-**6** as an orange solid (40 mg, 27%). R_f = 0.21 (cyclohexane/EtOAc 7:3 + 0.25% FA). M.p.: 123.0-125.6°C. ^{1}H NMR (500 MHz, $(CD_3)_2CO$): δ (ppm) = 8.44 (d, *J* = 1.9 Hz, 1H), 8.03 (dd, *J* = 8.7, 1.9 Hz, 1H), 7.95 (d, *J* = 8.7 Hz, 1H), 7.84 (d, *J* =

9.2 Hz, 1H), 6.88 (d, J = 2.7 Hz, 1H), 6.81 (dd, J = 9.3, 2.8 Hz, 1H), 4.76 (t, J = 7.0 Hz, 1H), 3.15 (s, 6H), 2.19 – 2.10 (m, 2H), 1.62 – 1.52 (m, 2H), 1.42 (dq, J = 14.3, 6.8 Hz, 2H), 1.36 – 1.27 (m, 4H), 0.88 (h, J = 4.1 Hz, 3H). ^{13}C NMR (126 MHz, $(CD_3)_2CO$): δ (ppm) = 172.1, 168.2, 154.9, 154.2, 151.0, 139.3, 139.2, 137.3, 122.6, 122.0, 119.1, 116.8, 112.5, 111.8, 51.1, 40.3 (2 x *C*), 33.0, 32.3, 29.5, 27.6, 23.2, 14.3. IR (KBr, cm^{-1}): $\tilde{\nu}$ = 3083 (w), 3070 (w), 2926 (s), 2856 (m), 1728 (s), 1599 (vs), 1366 (s), 826 (s). HPLC purity > 95%. HRMS (ESI): *m/z* calculated 491.1336 for $C_{23}H_{28}ClN_4O_2S_2$, found 491.1331 ($[M+H]^+$).

Ethyl 2-((6-aminobenzo[*d*]thiazol-2-yl)thio)octanoate (8). The compound was synthesized following literature procedure[8] from benzo[*d*]thiazole-2-thiol **(7)** R_f = 0.27 (cyclohexane/EtOAc 8:2). ^{1}H-NMR (400 MHz, $(CD_3)_2SO$): δ (ppm) = 7.53 (d, J = 8.7 Hz, 1H), 7.00 (d, J = 2.2 Hz, 1H), 6.73 (dd, J = 8.7, 2.3 Hz, 1H), 5.42 (s, 2H), 4.34 (t, J = 7.2 Hz, 1H), 4.16 – 4.08 (m, 2H), 1.95 – 1.79 (m, 2H), 1.44 – 1.22 (m, 8H), 1.14 (t, J = 7.1 Hz, 3H), 0.84 (t, J = 6.7 Hz, 3H).^{13}C-NMR (101 MHz, $(CD_3)_2SO$): δ (ppm) = 170.6, 154.8, 147.1, 144.2, 137.3, 122.0, 114.7, 103.6, 61.1, 49.9, 31.3, 30.9, 28.0, 26.3, 21.9, 13.9, 13.9. HRMS (ESI): *m/z* calculated 353.1357 for $C_{17}H_{25}N_2O_2S_2$, found 353.1327 ($[M+H]^+$).

2-((6-Aminobenzo[*d*]thiazol-2-yl)thio)octanoic acid (9). 8 (0.90 g, 2.6 mmol, 1.0 equiv.) was dissolved in 35 mL of THF. LiOH (0.31 g, 13 mmol, 5.0 equiv.) dissolved in 5 mL of distilled water was added dropwise. The reaction was stirred at rt for 18 h. THF was evaporated under reduced pressure and the aqueous residue was diluted with water and acidified with 2 M HCl. Then, the product was extracted with EtOAc (3 x 30 mL) and the combined organic layers were dried with $MgSO_4$. The solvent was evaporated under reduced pressure and the crude product was purified by reverse phase chromatography (C18, 80-30% water in acetonitrile) obtaining **9** as a white solid (0.60 g, 73%). R_f = 0.49 (cyclohexane/EtOAc 1:1 + 0.25% FA). ^{1}H-NMR (400 MHz, $(CD_3)_2SO$): δ (ppm) = 7.52 (d, J = 8.7 Hz, 1H), 7.00 (d, J = 2.2 Hz, 1H), 6.73 (dd, J = 8.7, 2.2 Hz, 1H), 4.30 (t, J = 7.1 Hz, 1H), 1.96 – 1.79 (m, 2H), 1.44 – 1.20 (m, 8H), 0.84 (t, J = 6.8 Hz, 3H).^{13}C-NMR (101 MHz, $(CD_3)_2SO$): δ (ppm) = 172.0, 155.7, 147.0, 144.2, 137.1, 122.0, 114.6, 103.7, 50.3, 31.7, 31.0, 28.1, 26.3, 22.0, 13.9. HRMS (ESI): *m/z* calculated 325.1044 for $C_{15}H_{21}N_2O_2S_2$, found 325.1016 ($[M+H]^+$).

2-((*tert*-Butyldimethylsilyl)oxy)-4-chloroaniline (13). 2-Amino-5-chlorophenol (0.72 g, 5.0 mmol, 1.0 equiv.) and imidazole (1.0 g, 15 mmol, 3.0 equiv.) were dissolved in 5 mL DMF and the mixture was cooled to 0 °C. TBDMSCl (1.1 g, 7.5 mmol, 1.5 equiv.) was added in portions. The reaction mixture was allowed to warm to ambient temperature and stirred overnight. The solvent was removed under reduced pressure and the crude product was purified by flash chromatography (SiO_2; gradient: 0-10% EtOAc in cyclohexane) obtaining **13** as a brown oil (1.2 g, 93%). R_f = 0.54 (cyclohexane/EtOAc 8:2). ^{1}H-NMR (400 MHz, $CDCl_3$): δ (ppm) = 6.77 (dd, J = 8.3, 2.3 Hz, 1H), 6.73 (d, J = 2.2 Hz, 1H), 6.69 (d, J = 8.4 Hz, 1H), 1.01 (s, 9H), 0.26 (s, 6H). ^{13}C NMR (101 MHz, $CDCl_3$): δ (ppm) = 143.9, 136.2, 123.2, 121.8, 118.9, 116.6, 25.9 (3 x *C*), 18.4, -4.2 (2 x *C*).

NMR spectra, IR spectra and purity analysis of **2-6**

^{1}H NMR spectrum of *E*-**2** (400 MHz, $(CD_3)_2SO$)

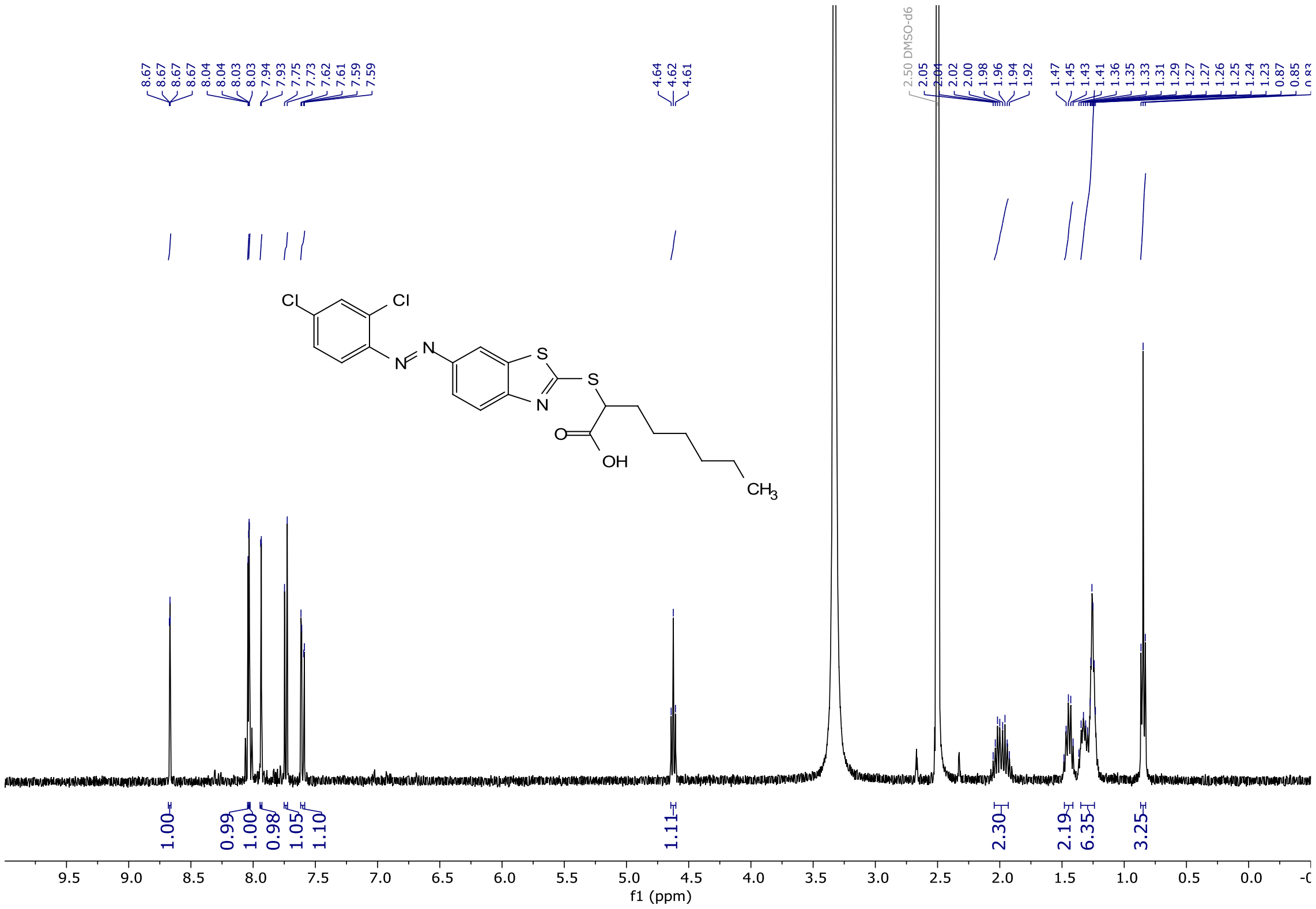

^{13}C NMR spectrum of *E*-**2** (101 MHz, $(CD_3)_2SO$)

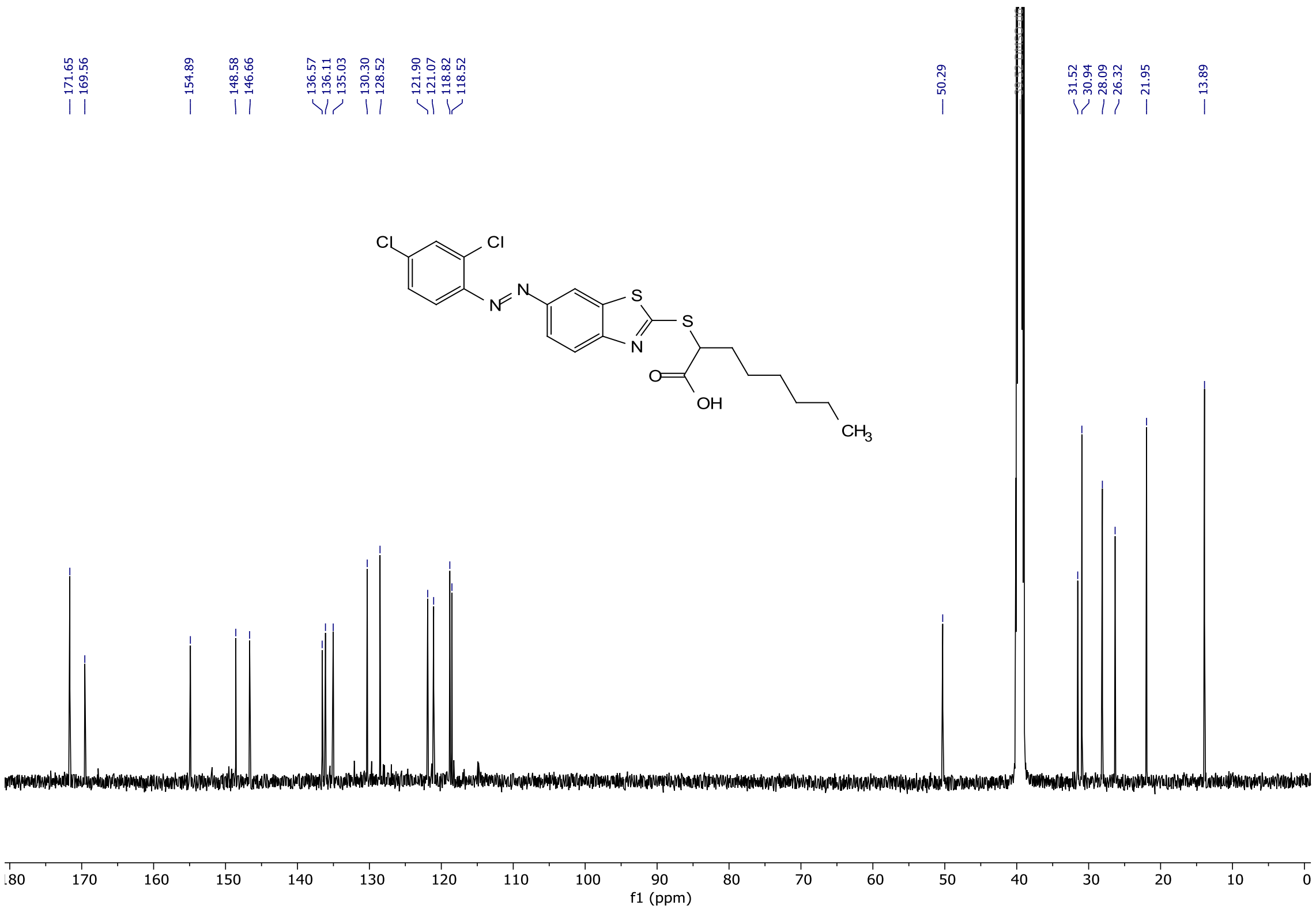

IR spectrum of *E*-**2**

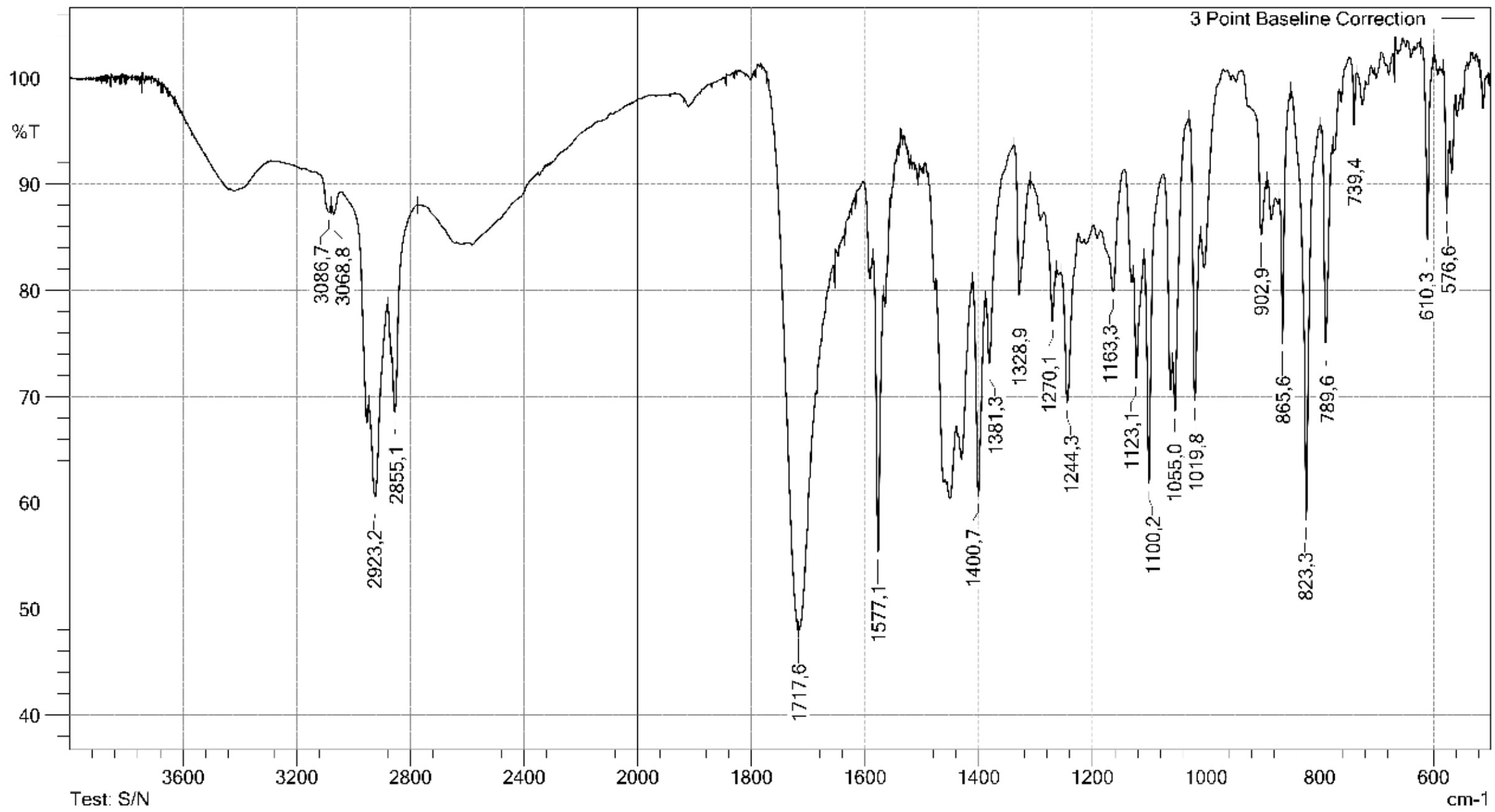

HPLC purity of *E*-**2**

==== Shimadzu LabSolutions Browser Report ====

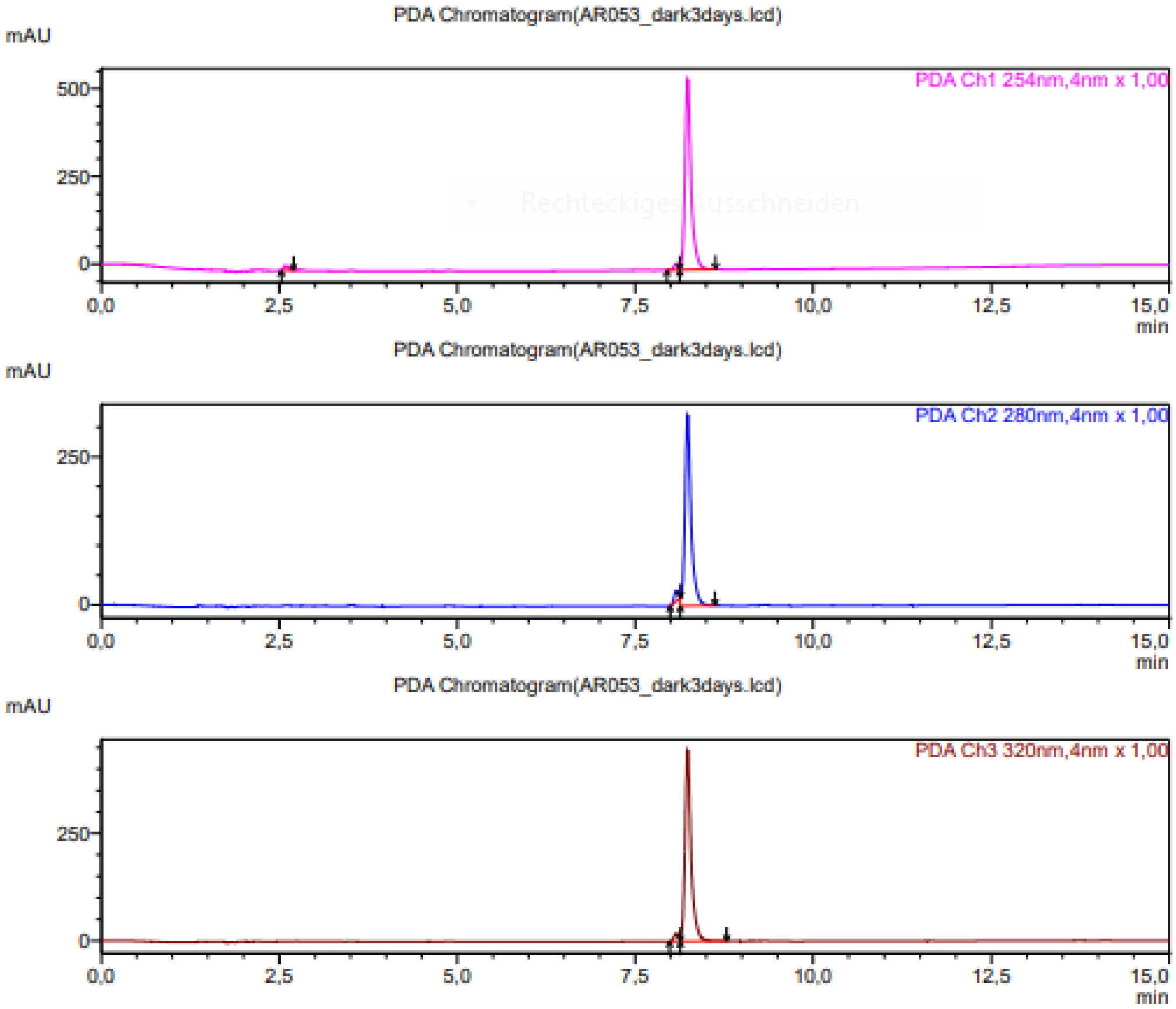

PDA Peak Table(AR053_dark3days.lcd)
PDA Ch1 254nm

Peak#	Ret. Time	Area	Area%
1	2,589	59236	1,755
2	8,082	85441	2,531
3	8,238	3231115	95,714
Total		3375792	100,000

PDA Peak Table(AR053_dark3days.lcd)
PDA Ch3 320nm

Peak#	Ret. Time	Area	Area%
1	8,081	92221	3,346
2	8,238	2664070	96,654
Total		2756291	100,000

PDA Peak Table(AR053_dark3days.lcd)
PDA Ch3 320nm

Peak#	Ret. Time	Area	Area%
1	8,081	92221	3,346
2	8,238	2664070	96,654
Total		2756291	100,000

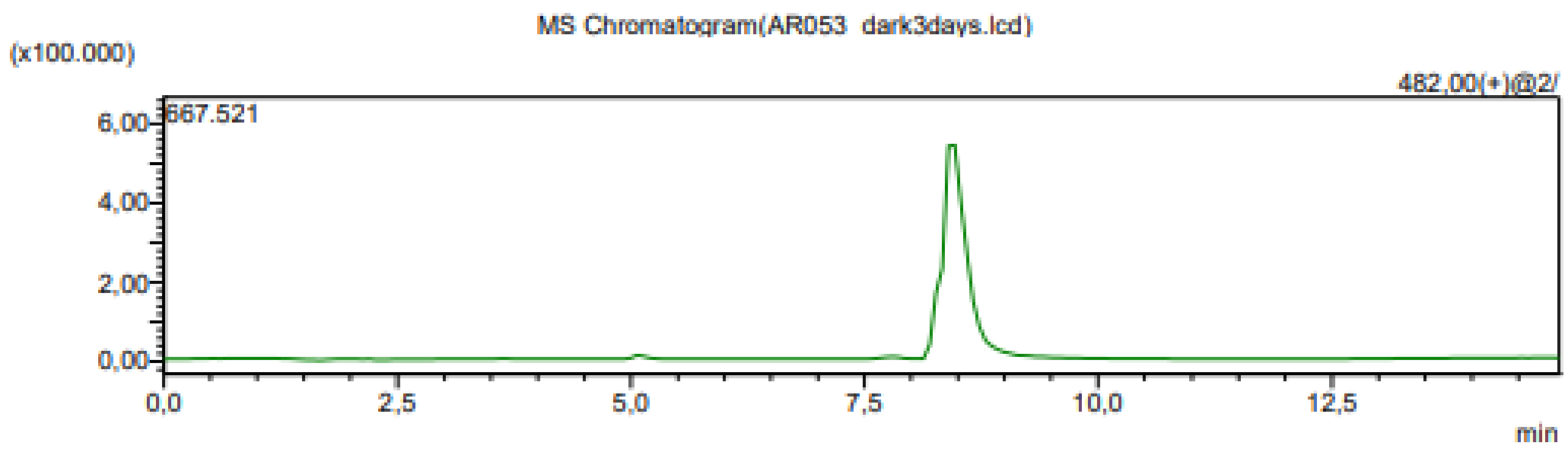

^{1}H NMR spectrum of *E*-**3** (500 MHz, $(CD_3)_2SO$)

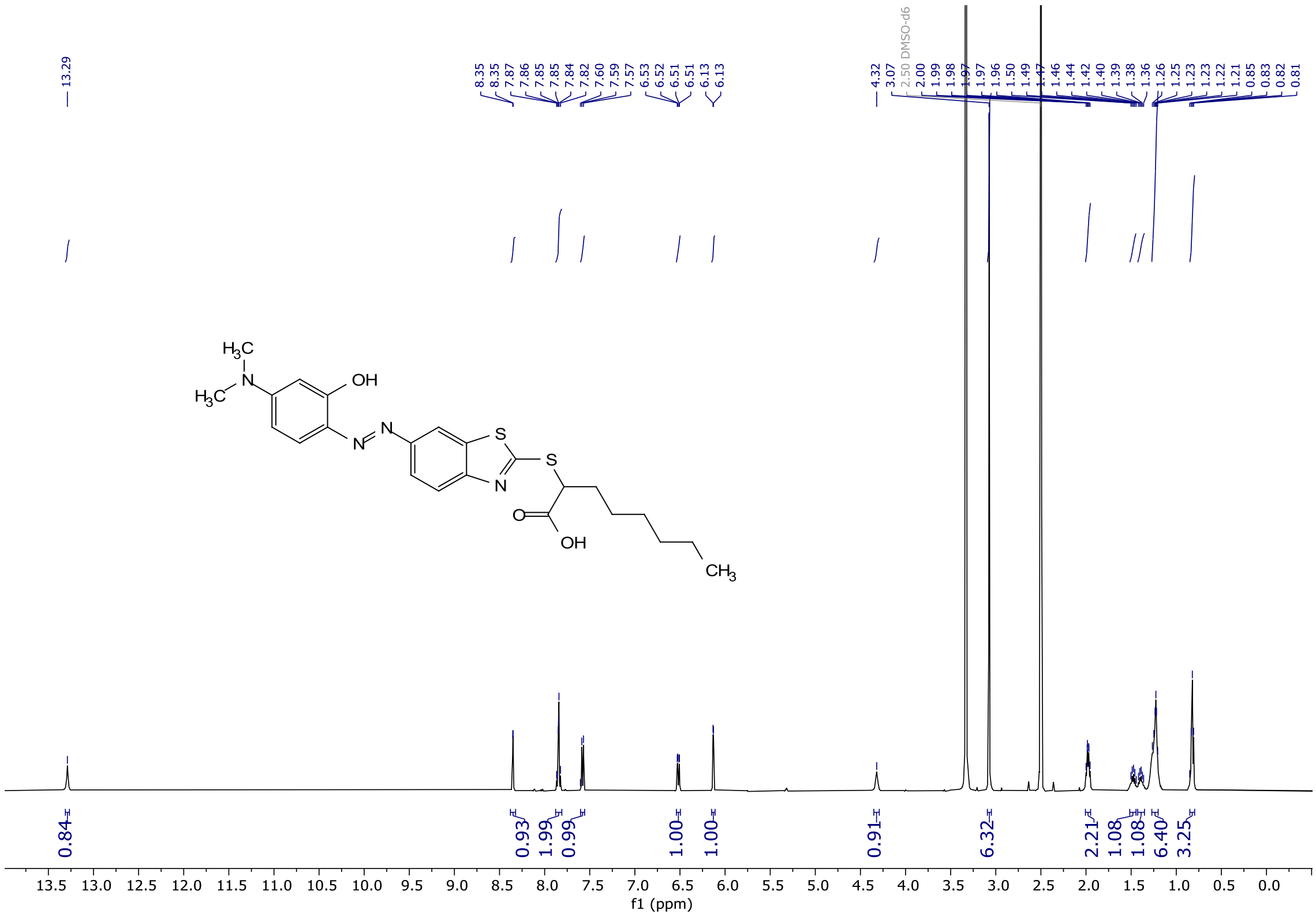

^{13}C NMR spectrum of *E*-**3** (126 MHz, $(CD_3)_2SO$)

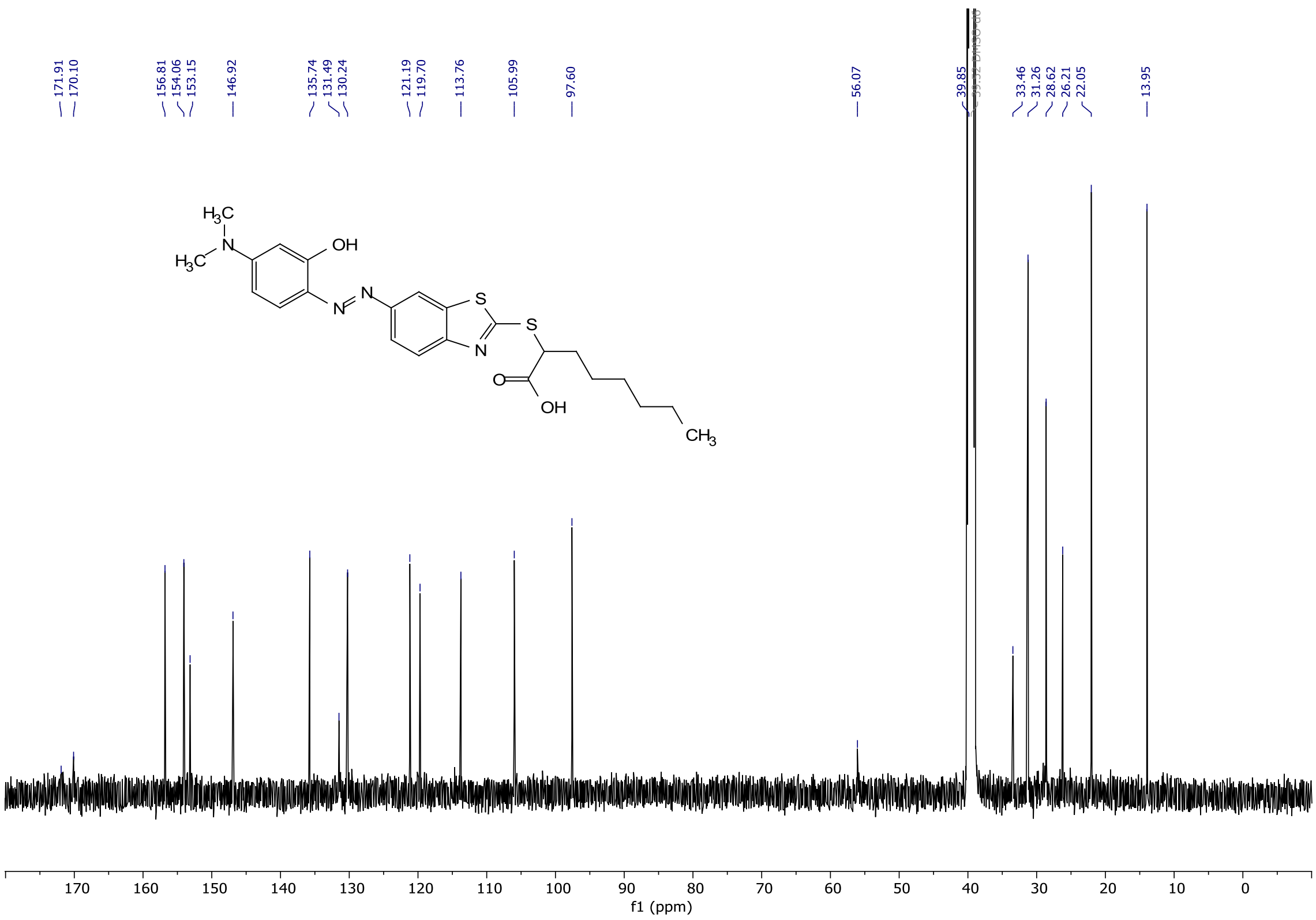

IR spectrum of *E*-**3**

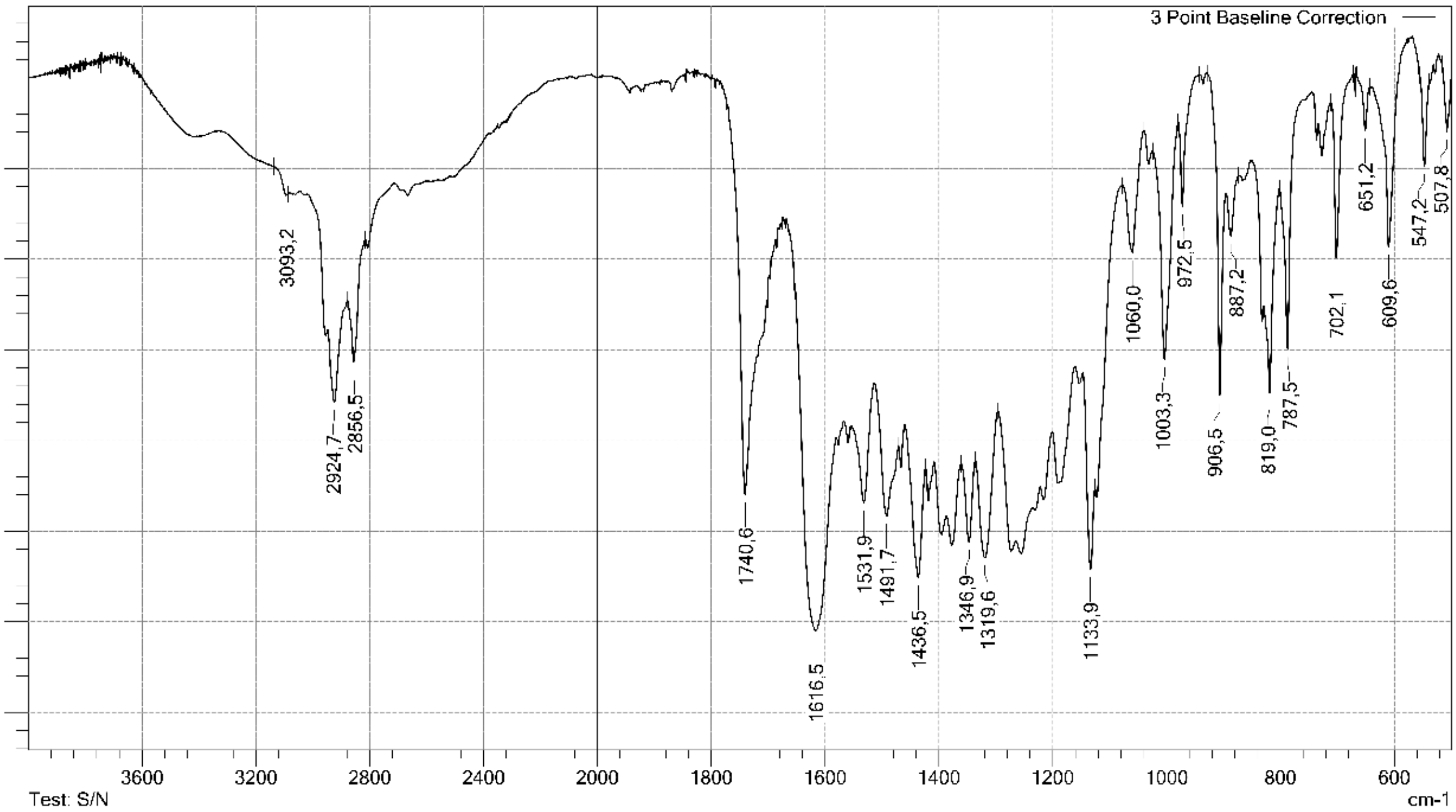

HPLC purity of *E*-**3**

==== Shimadzu LabSolutions Browser Report ====

PDA Chromatogram(LK173_Deg_10mM.lcd)

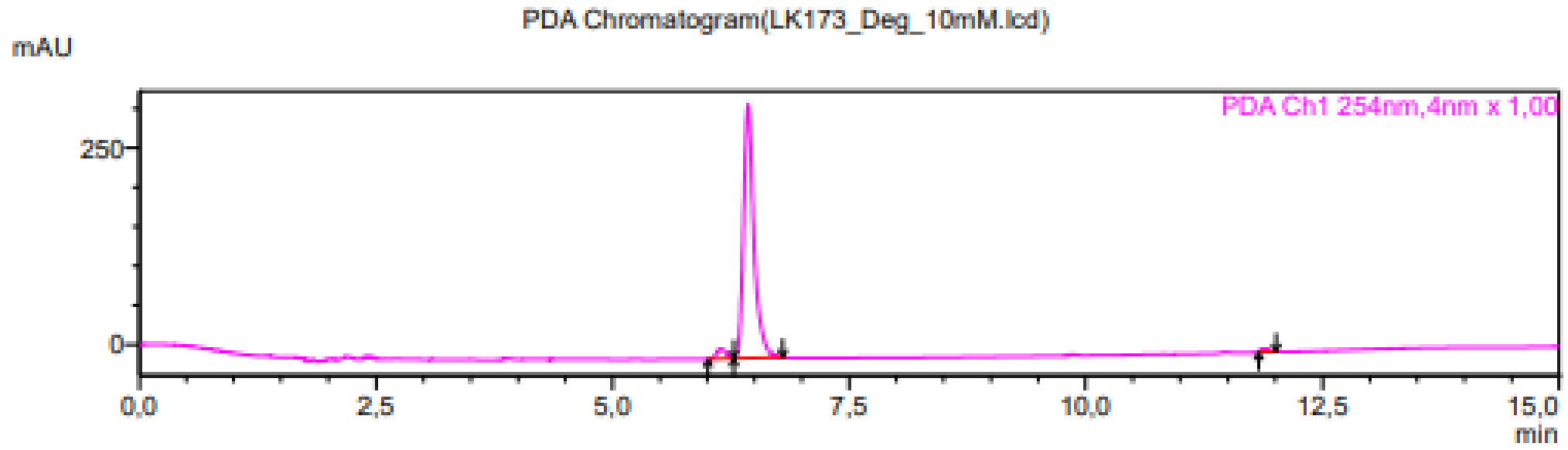

PDA Chromatogram(LK173_Deg_10mM.lcd)

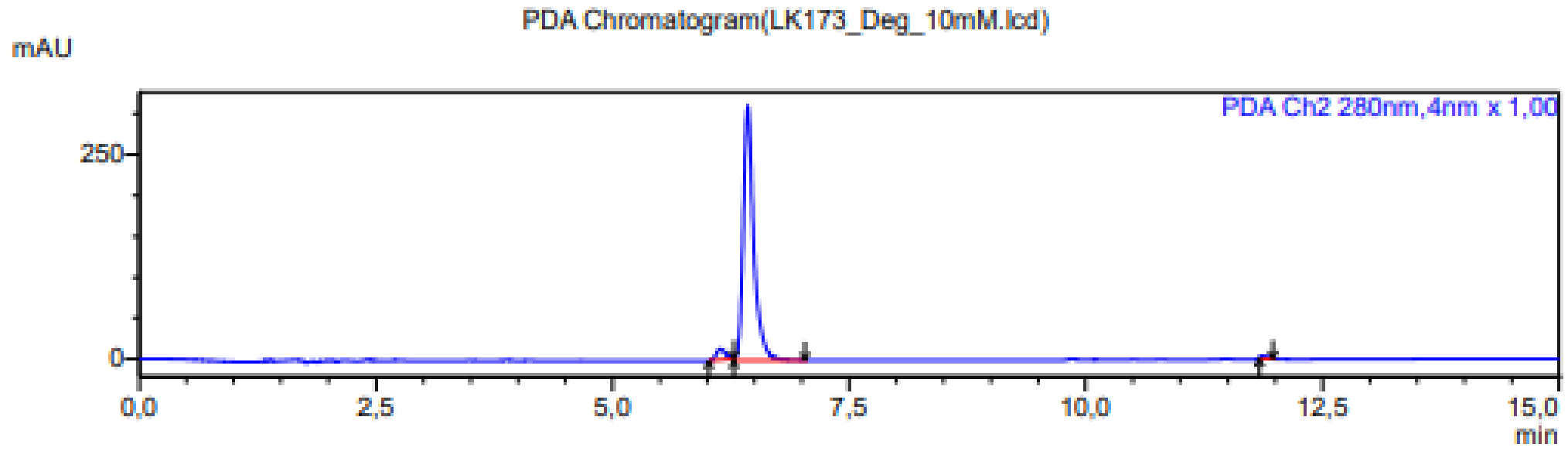

PDA Chromatogram(LK173_Deg_10mM.lcd)

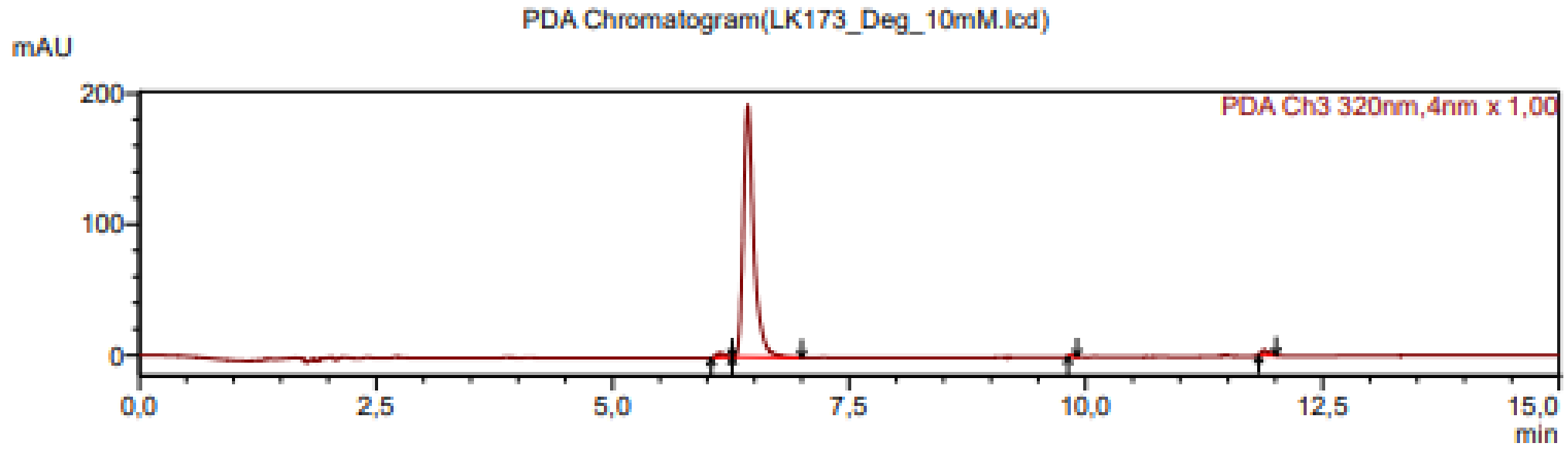

PDA Peak Table(LK173_Deg_10mM.lcd)
PDA Ch1 254nm

Peak#	Ret. Time	Area	Area%
1	6,143	94746	3,684
2	6,430	2455948	95,501
3	11,880	20951	0,815
Total		2571646	100,000

PDA Peak Table(LK173_Deg_10mM.lcd)
PDA Ch2 280nm

Peak#	Ret. Time	Area	Area%
1	6,143	87850	3,521
2	6,430	2393467	95,932
3	11,879	13641	0,547
Total		2494957	100,000

PDA Peak Table(LK173_Deg_10mM.lcd)
PDA Ch3 320nm

Peak#	Ret. Time	Area	Area%
1	6,143	20113	1,324
2	6,430	1475400	97,129
3	9,859	2922	0,192
4	11,879	20583	1,355
Total		1519018	100,000

MS Chromatogram(LK173_Deg_10mM.lcd)

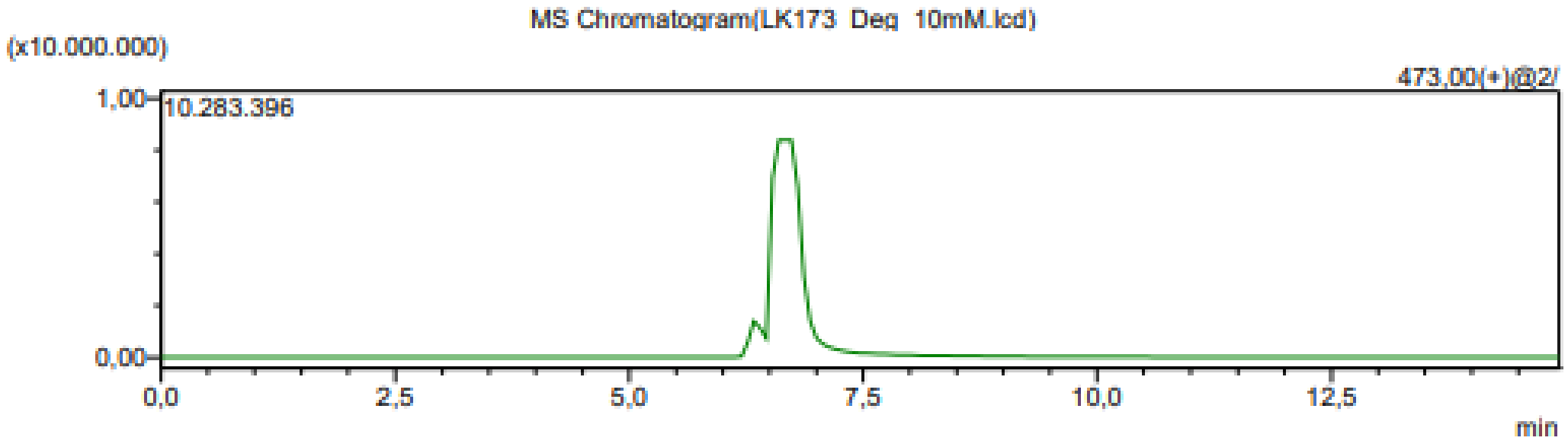

^{1}H NMR spectrum of *E*-**4** (400 MHz, $(CD_3)_2CO$)

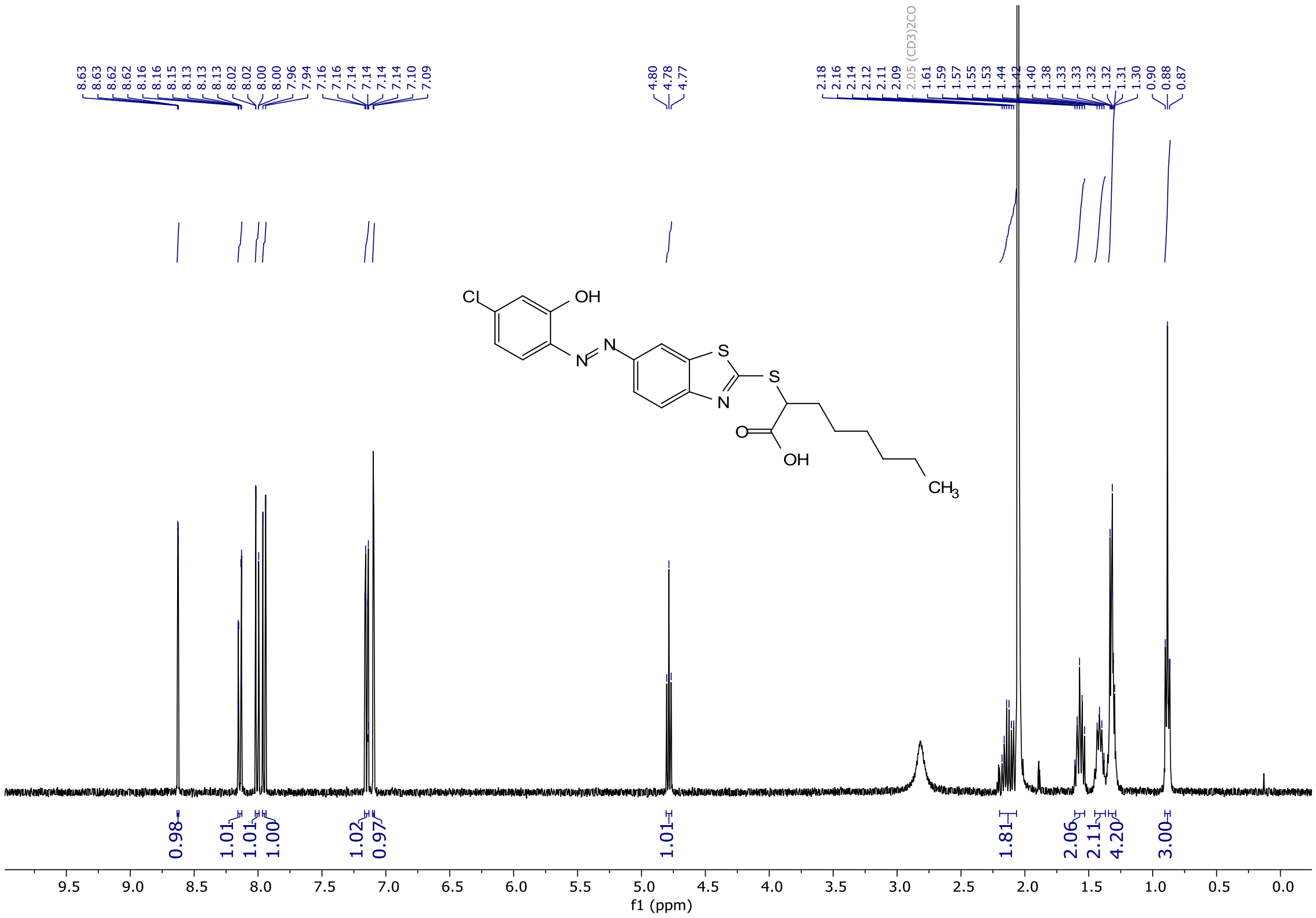

^{13}C NMR spectrum of *E*-**4** (101 MHz, $(CD_3)_2CO$)

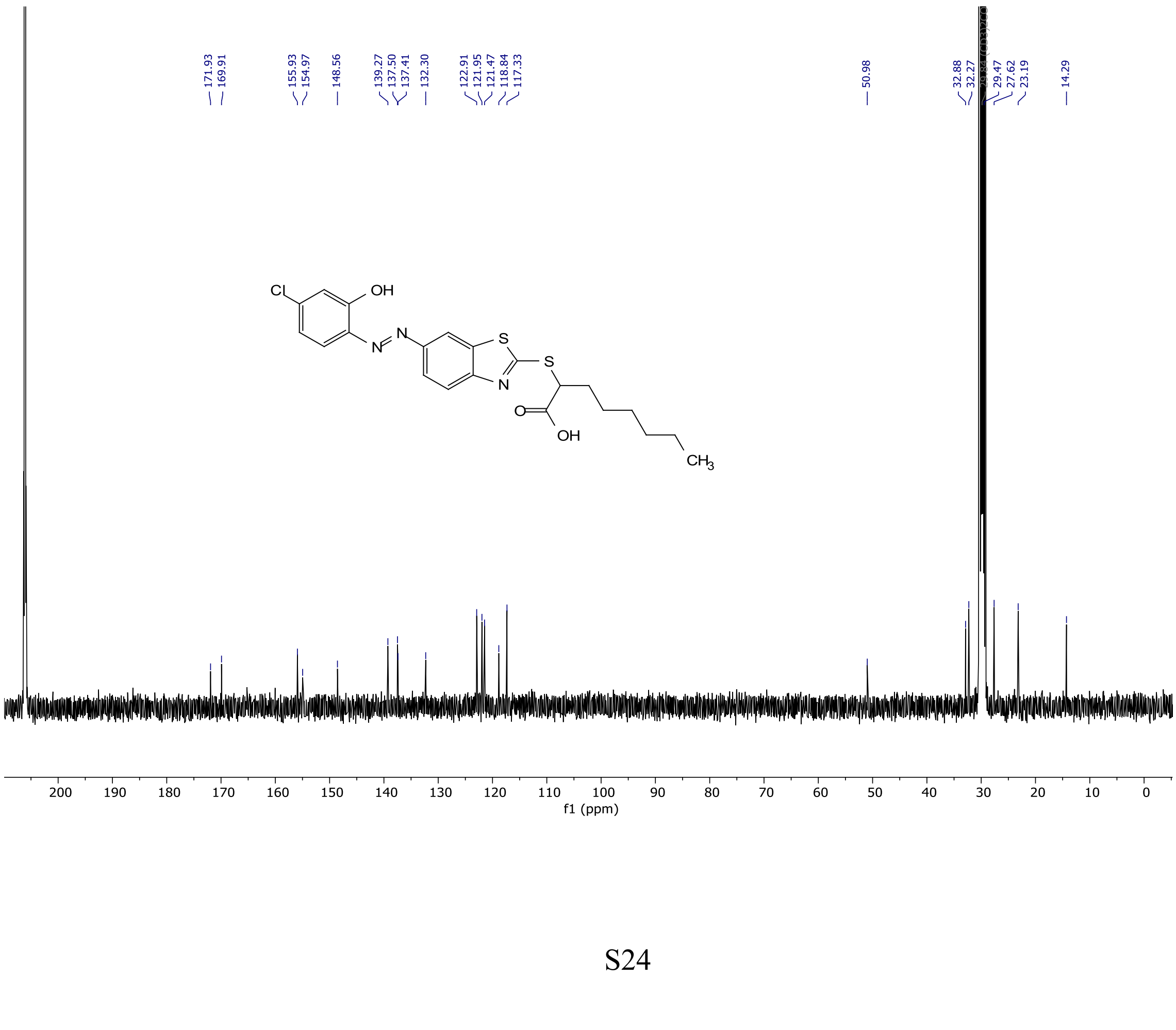

IR spectrum of *E*-**4**

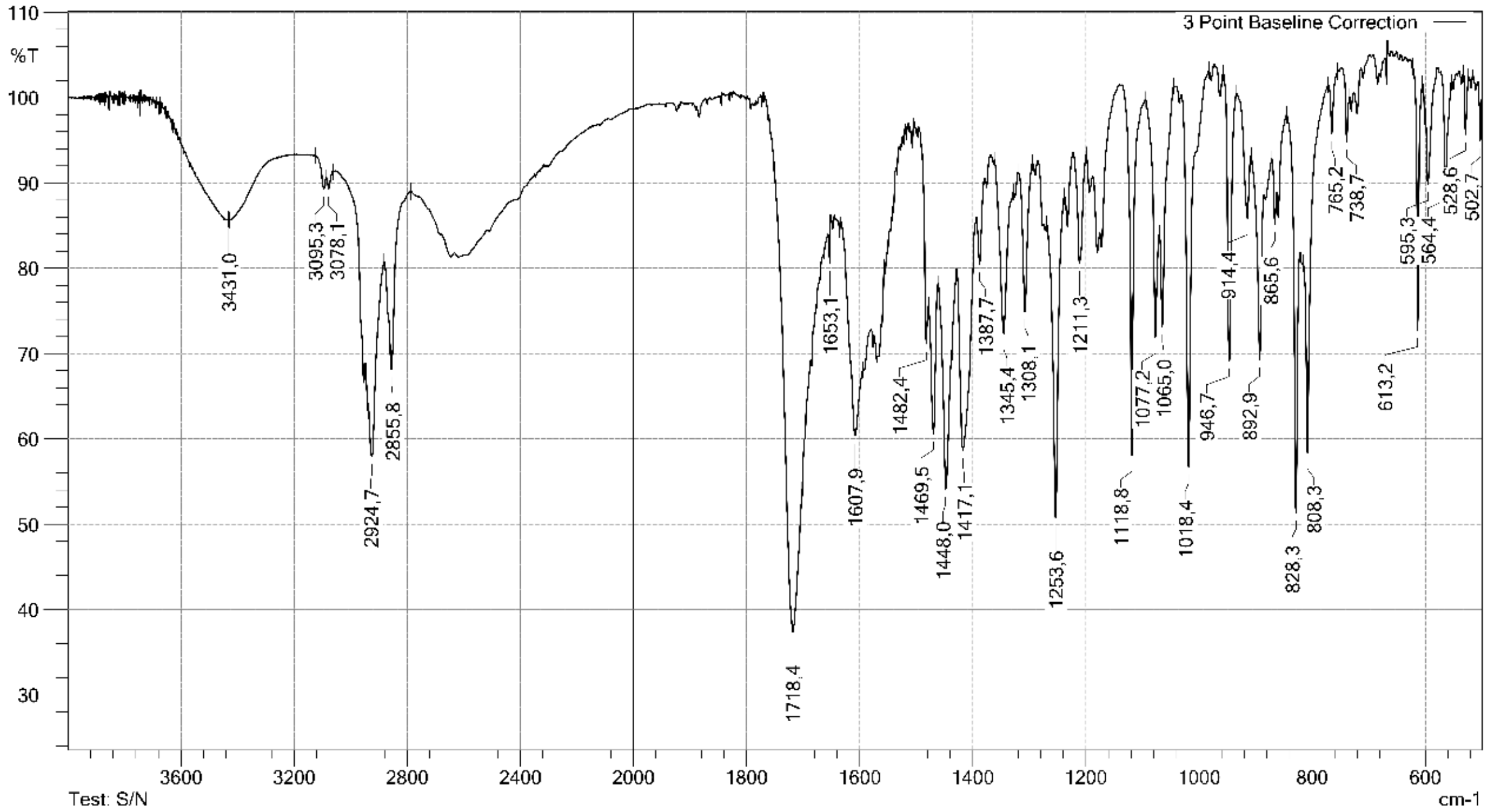

HPLC purity of *E*-**4**

==== Shimadzu LabSolutions Browser Report ====

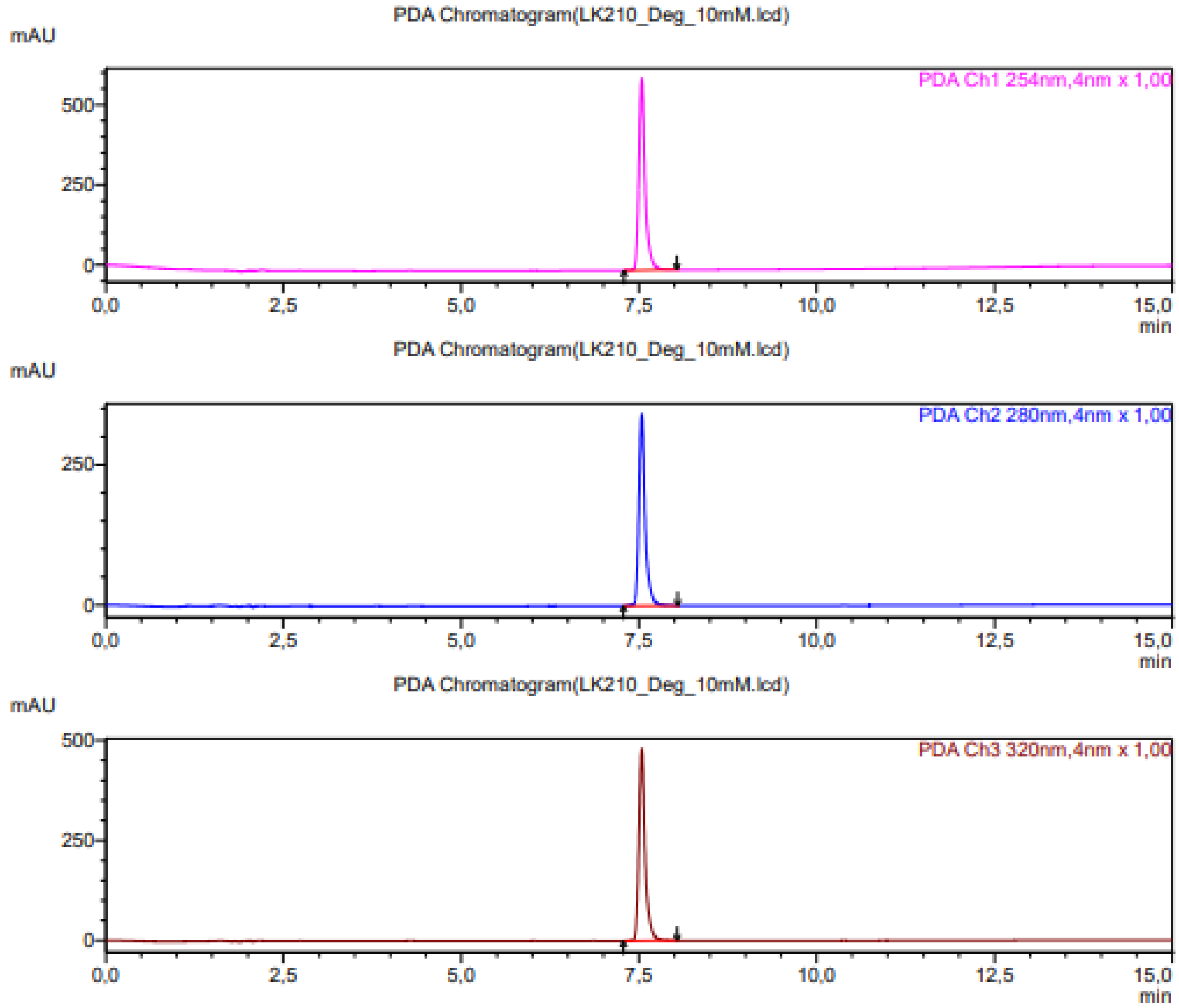

PDA Peak Table(LK210_Deg_10mM.lcd)
PDA Ch1 254nm

Peak#	Ret. Time	Area	Area%
1	7,540	3597688	100,000
Total		3597688	100,000

PDA Peak Table(LK210_Deg_10mM.lcd)
PDA Ch2 280nm

Peak#	Ret. Time	Area	Area%
1	7,540	2063870	100,000
Total		2063870	100,000

PDA Peak Table(LK210_Deg_10mM.lcd)
PDA Ch3 320nm

Peak#	Ret. Time	Area	Area%
1	7,540	2891989	100,000
Total		2891989	100,000

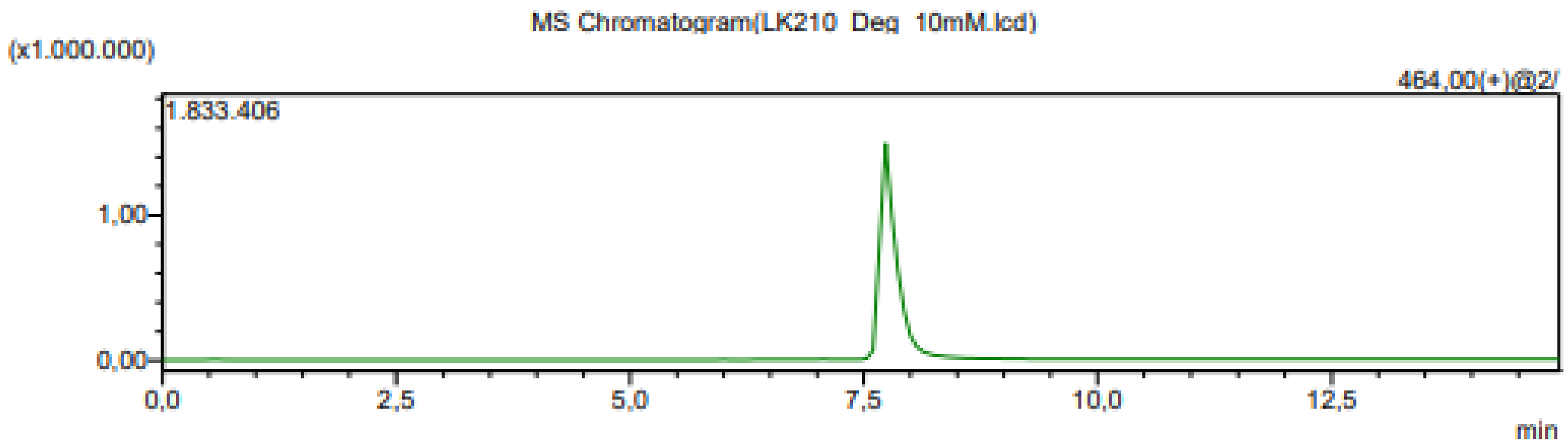

^{1}H NMR spectrum of *E*-**5** (500 MHz, $(CD_3)_2SO$)

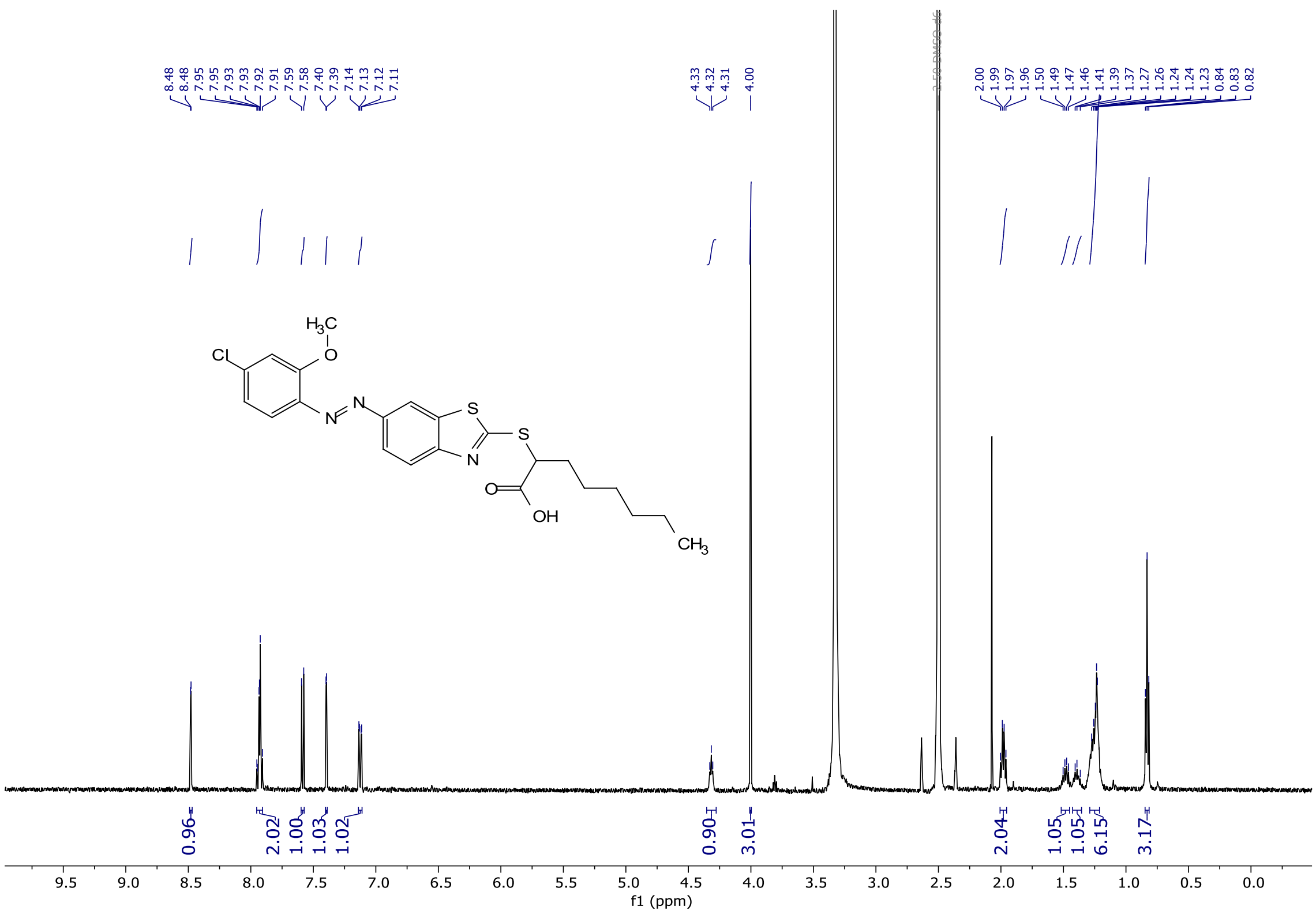

^{13}C NMR spectrum of *E*-**5** (126 MHz, $(CD_3)_2SO$)

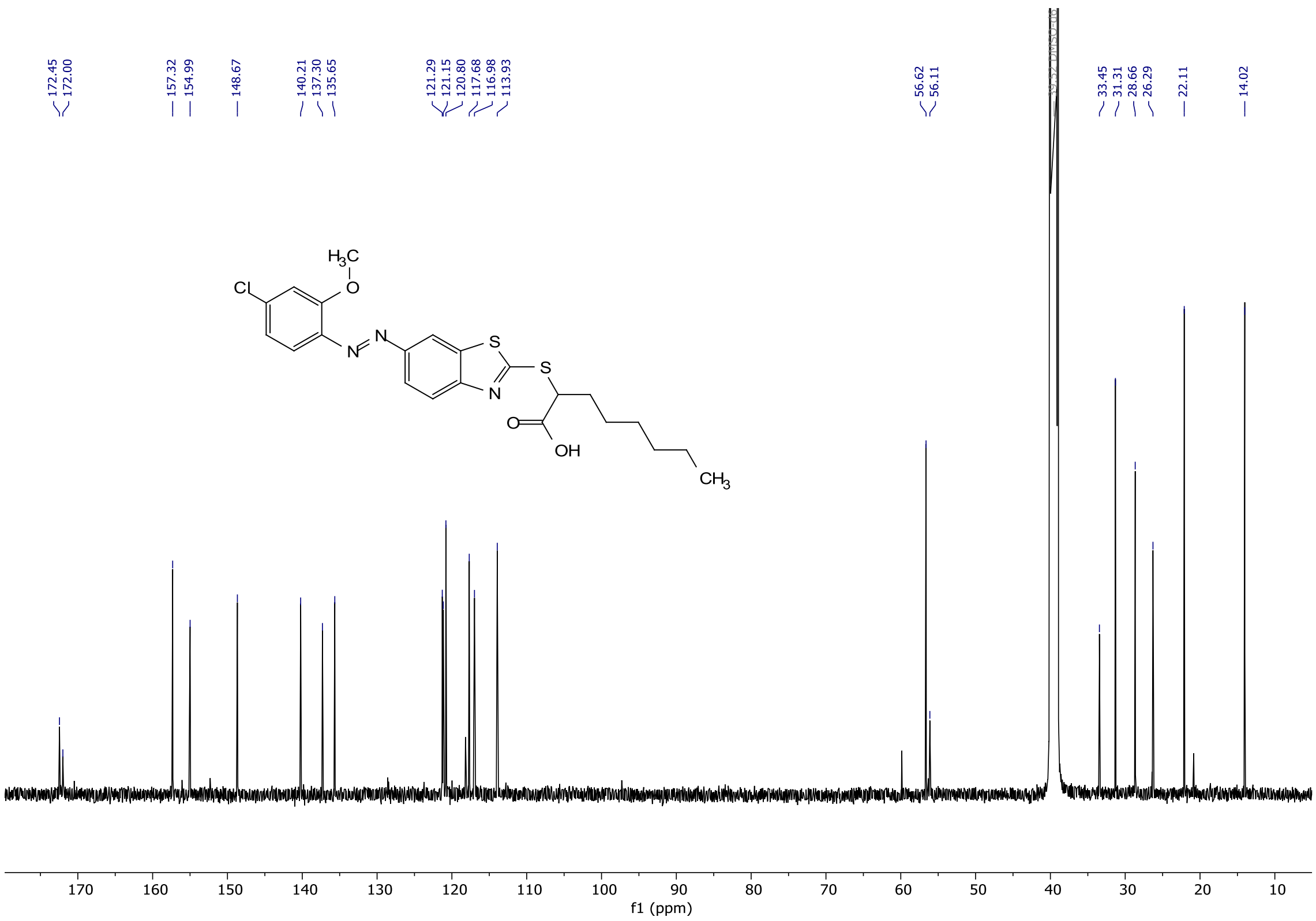

IR spectrum of *E*-**5**

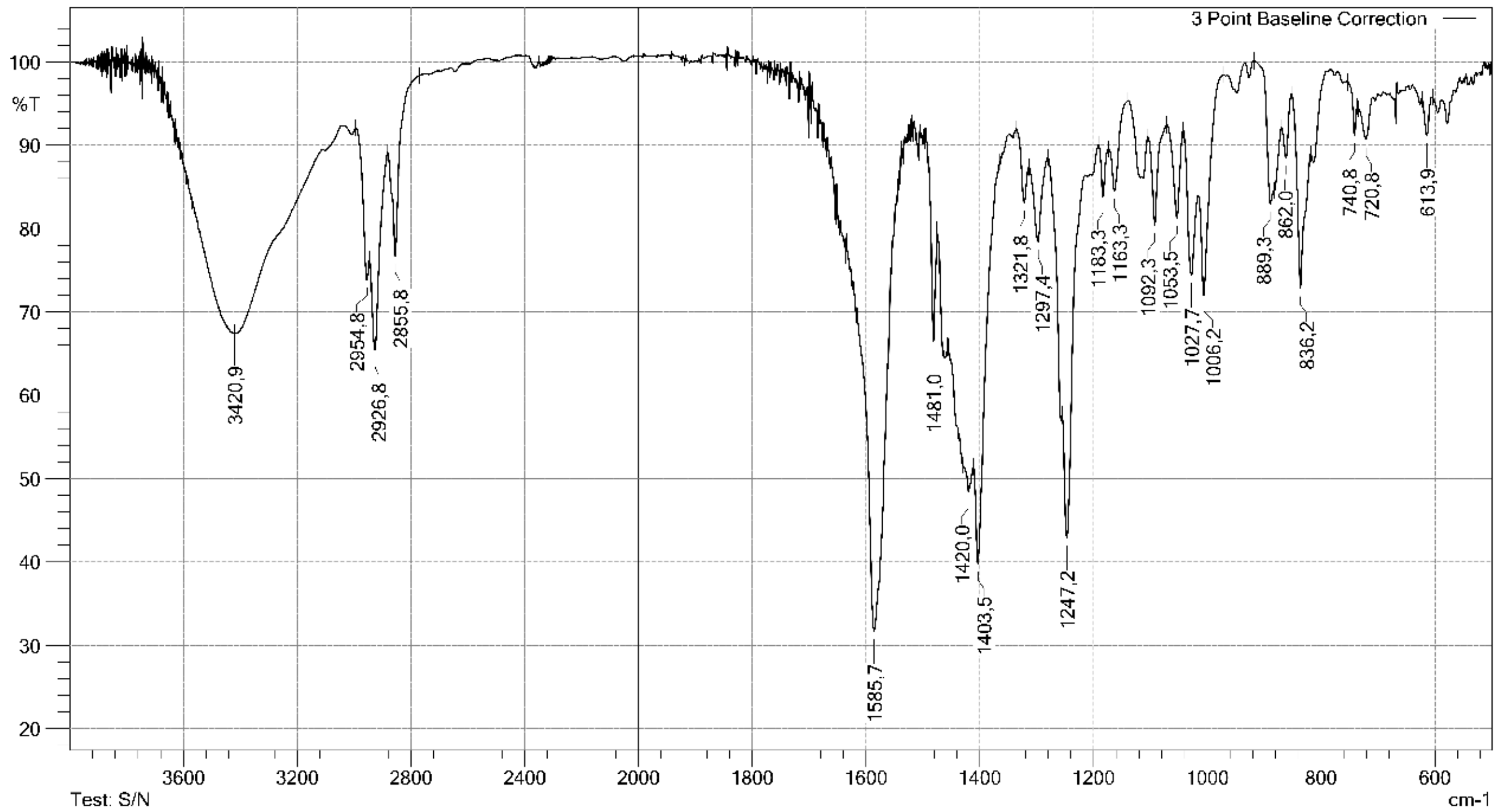

HPLC purity of *E*-**5**

==== Shimadzu LabSolutions Browser Report ====

PDA Chromatogram(LK158_dark3days.lcd)

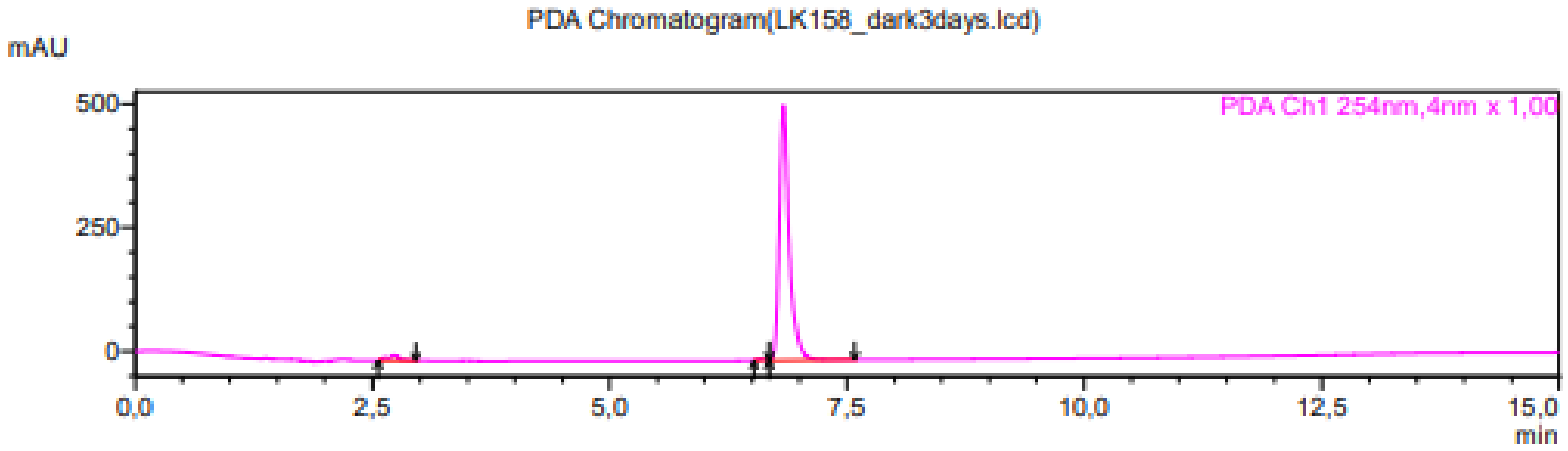

PDA Chromatogram(LK158_dark3days.lcd)

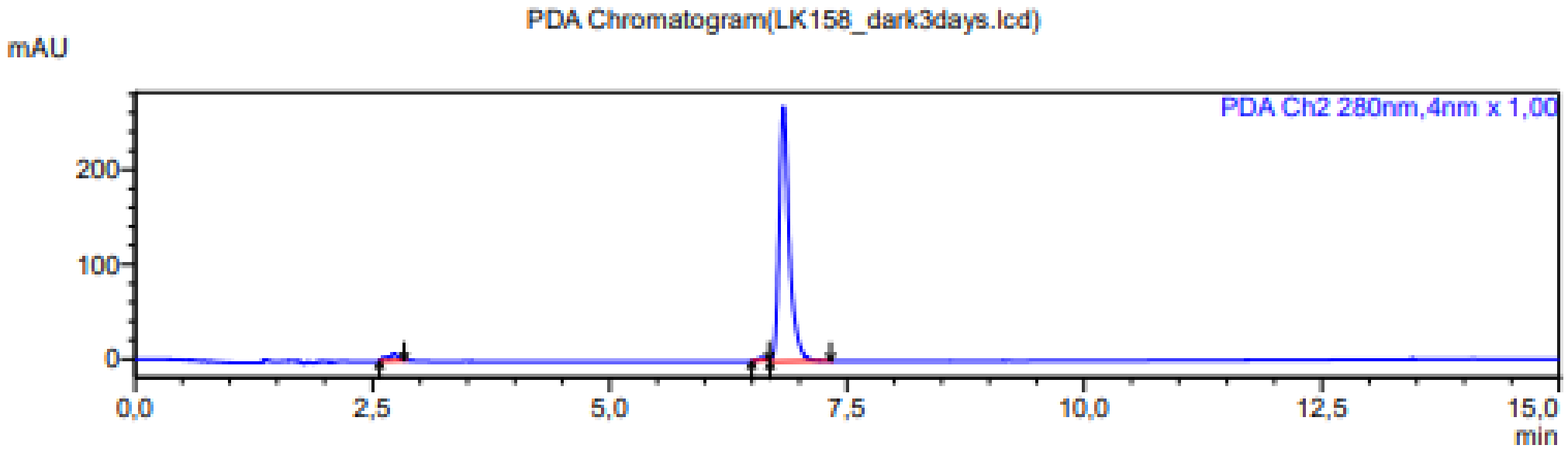

PDA Chromatogram(LK158_dark3days.lcd)

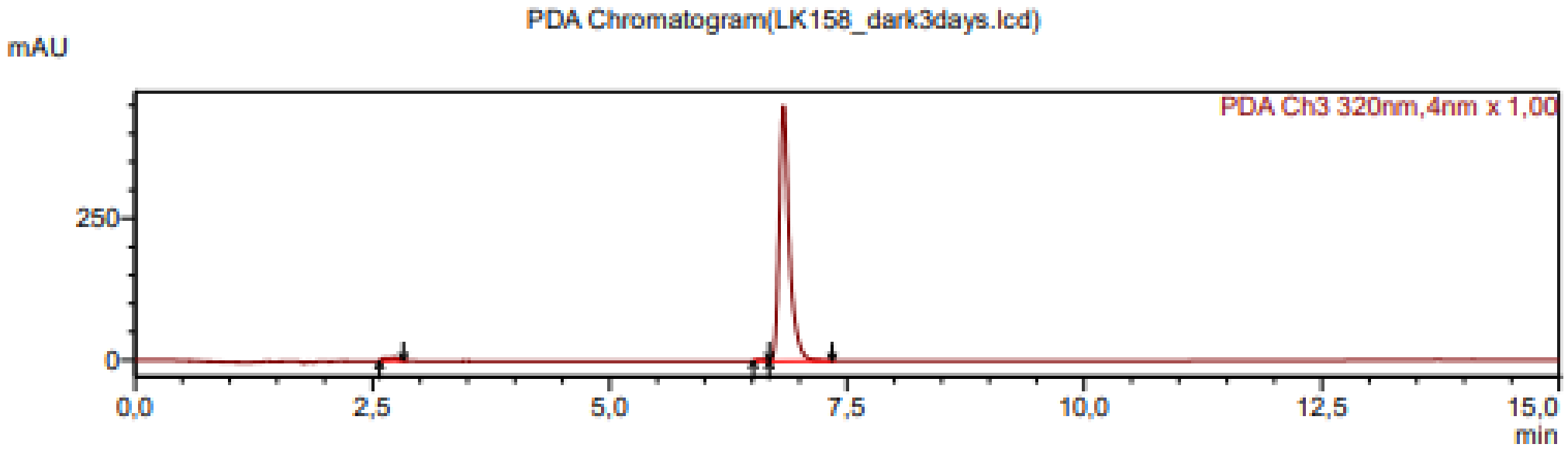

PDA Peak Table(LK158_dark3days.lcd)
PDA Ch1 254nm

Peak#	Ret. Time	Area	Area%
1	2,721	98065	2,496
2	6,601	10385	0,264
3	6,831	3820448	97,240
Total		3928898	100,000

PDA Peak Table(LK158_dark3days.lcd)
PDA Ch2 280nm

Peak#	Ret. Time	Area	Area%
1	2,721	54563	2,644
2	6,601	16215	0,786
3	6,831	1992672	96,570
Total		2063449	100,000

PDA Peak Table(LK158_dark3days.lcd)
PDA Ch3 320nm

Peak#	Ret. Time	Area	Area%
1	2,717	52627	1,553
2	6,600	9340	0,276
3	6,831	3327752	98,172
Total		3389719	100,000

MS Chromatogram(LK158_dark3days.lcd)

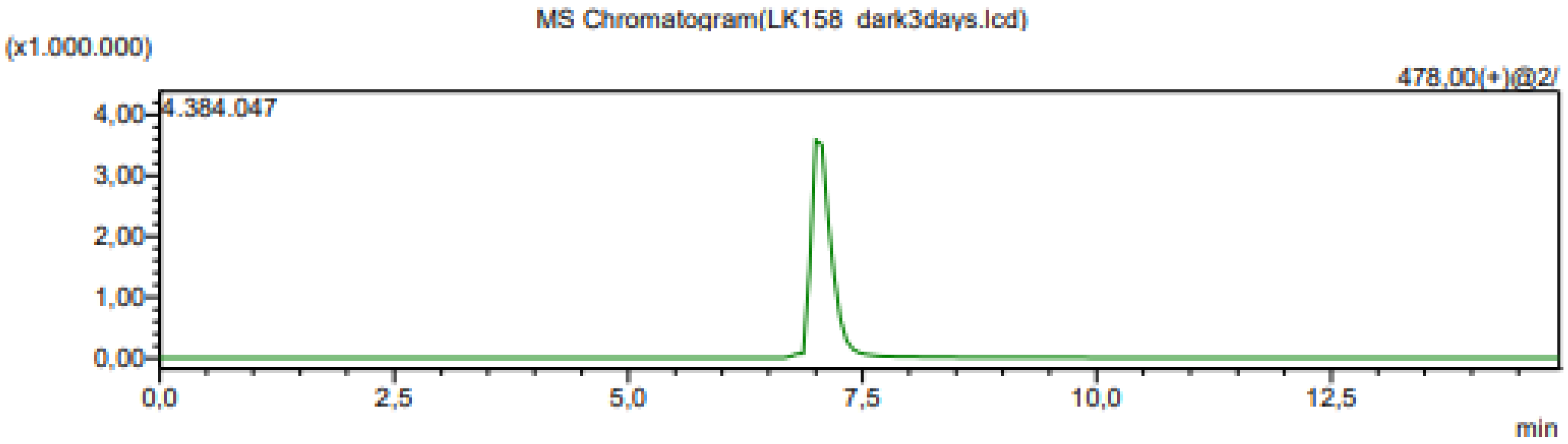

^{1}H NMR spectrum of *E*-**6** (500 MHz, $(CD_3)_2CO$)

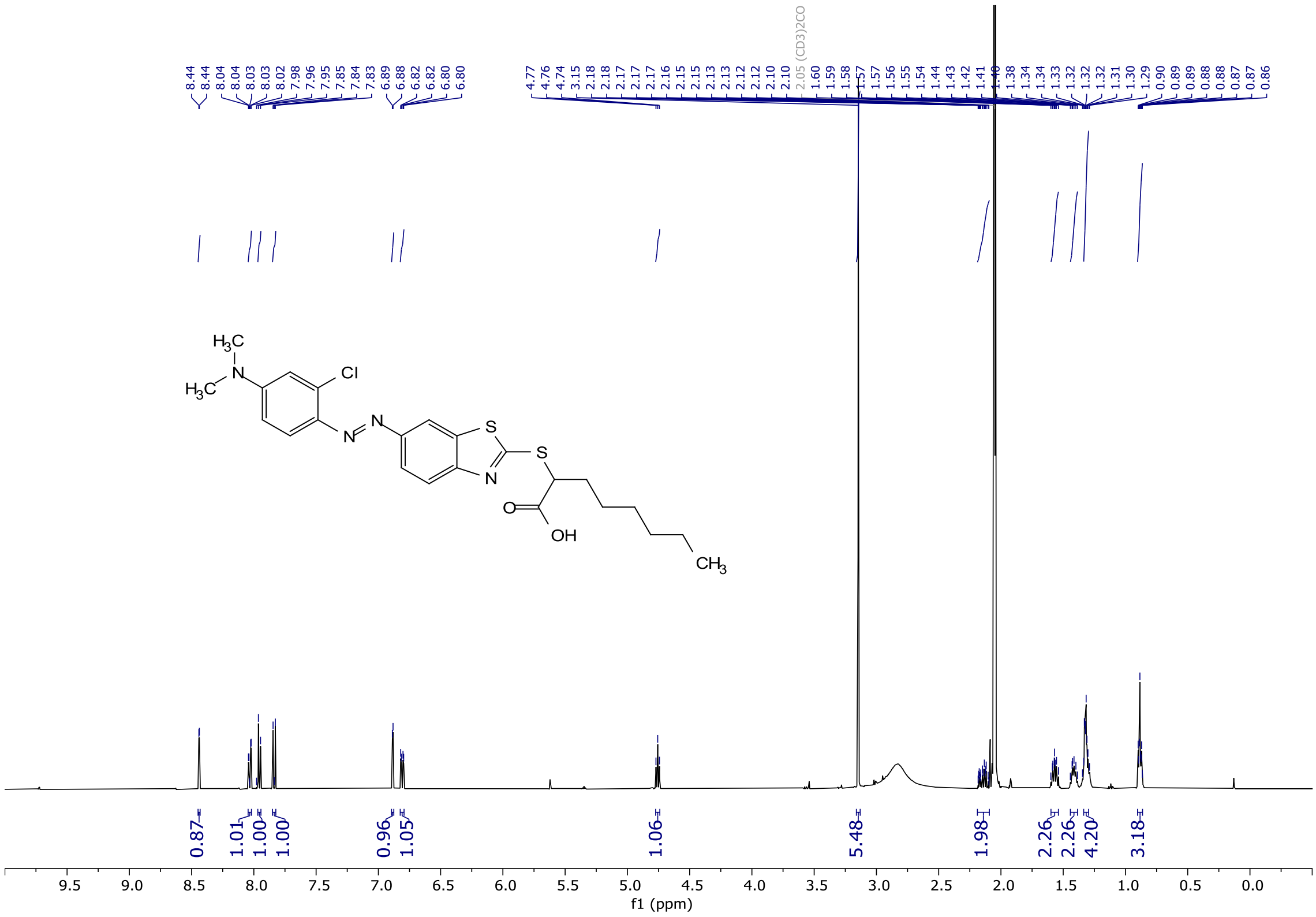

^{13}C NMR spectrum of *E*-**6** (126 MHz, $(CD_3)_2CO$)

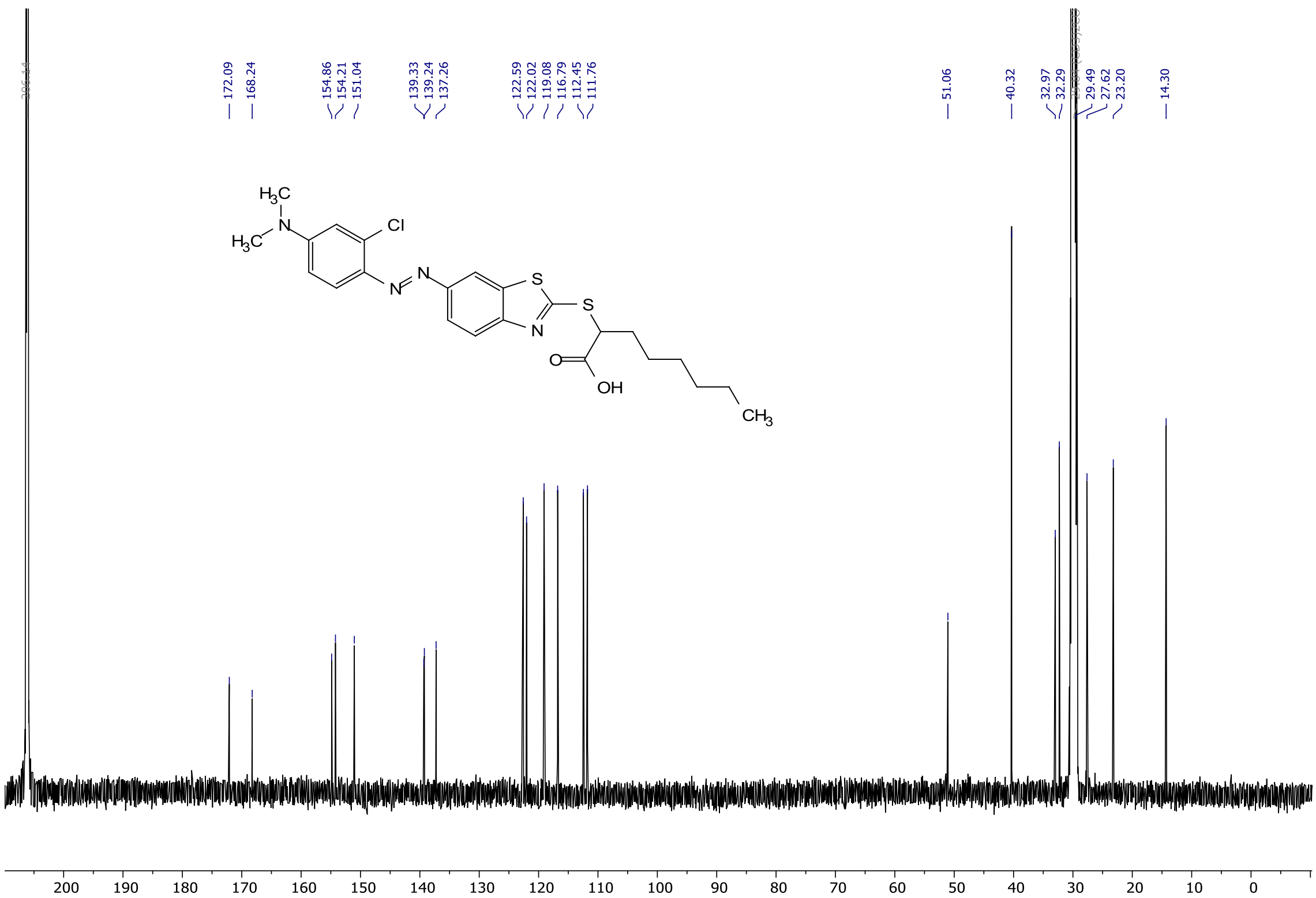

IR spectrum of *E*-**6**

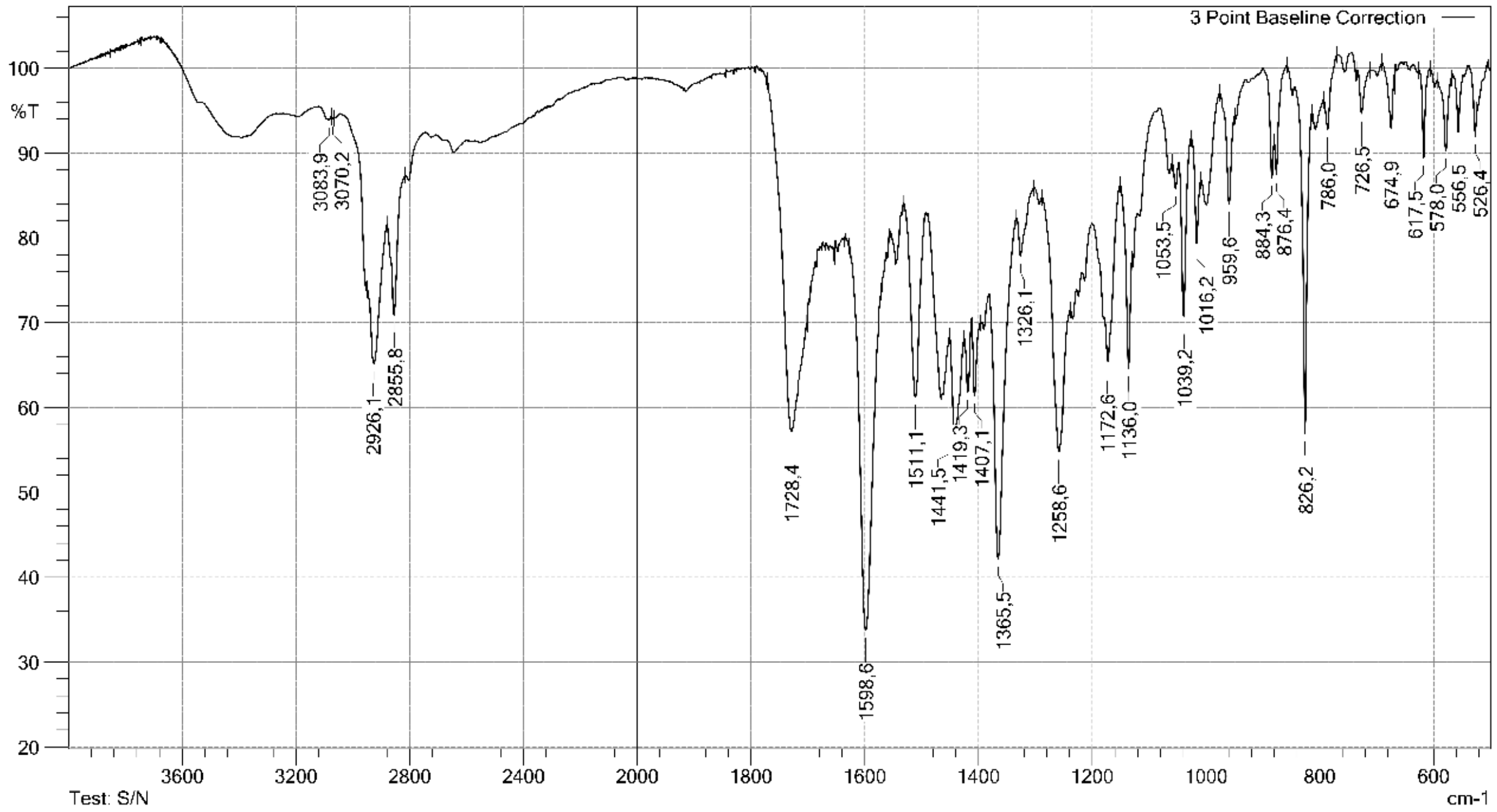

HPLC purity of *E*-**6**

==== Shimadzu LabSolutions Browser Report ====

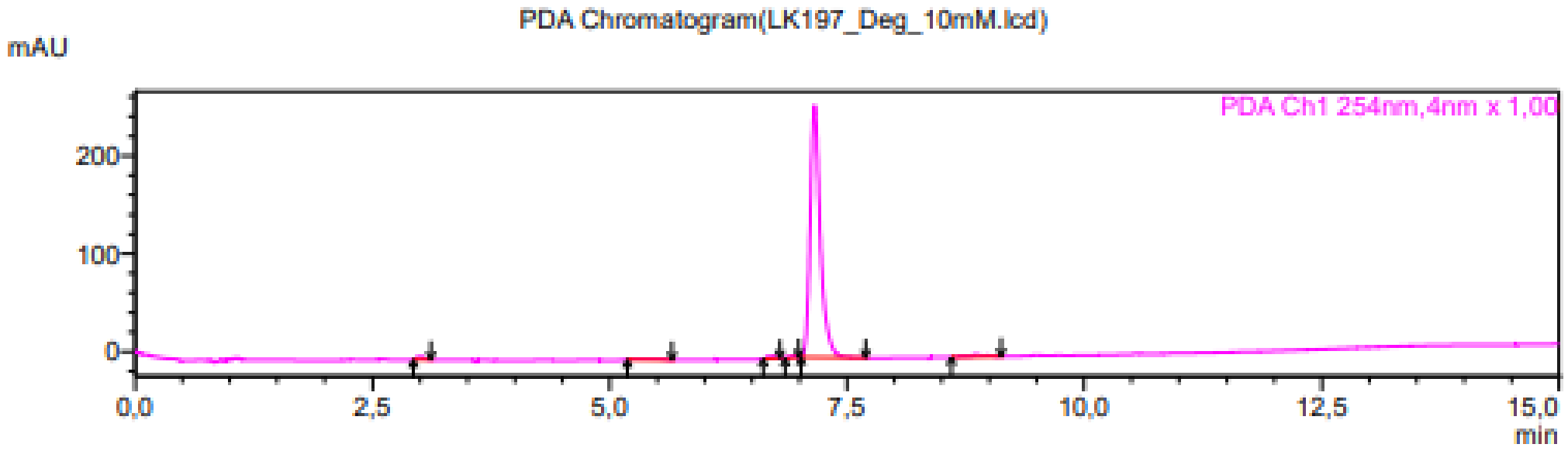

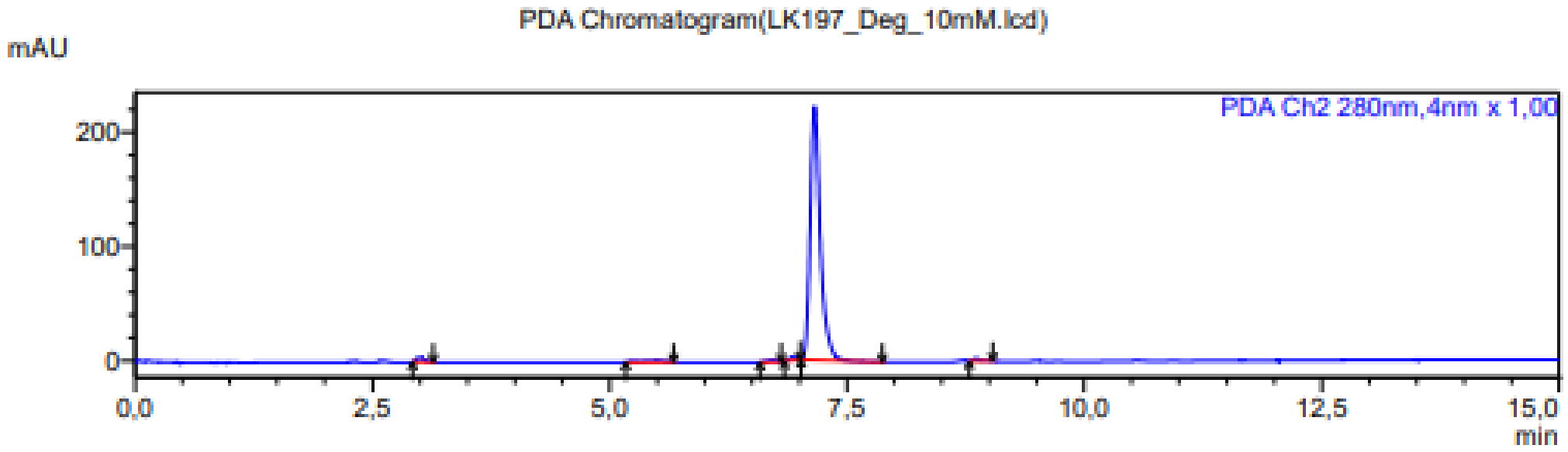

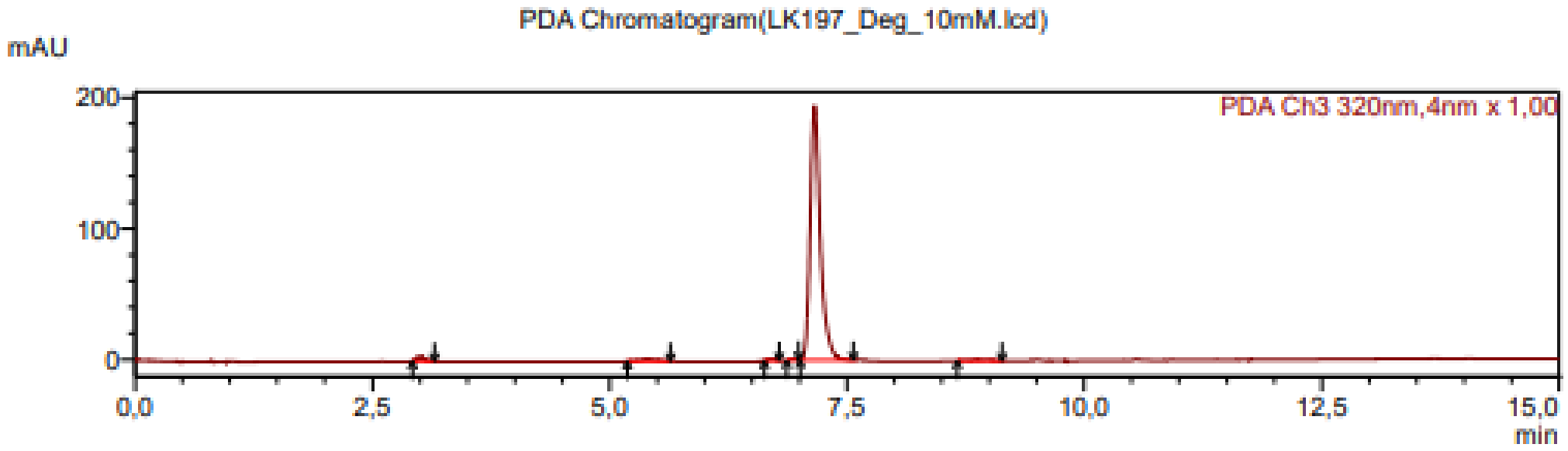

PDA Peak Table(LK197_Deg_10mM.lcd)
PDA Ch1 254nm

Peak#	Ret. Time	Area	Area%
1	2,995	15891	0,792
2	5,390	23783	1,185
3	6,709	12238	0,610
4	6,935	8316	0,414
5	7,160	1920263	95,692
6	8,864	26215	1,306
Total		2006706	100,000

PDA Peak Table(LK197_Deg_10mM.lcd)
PDA Ch2 280nm

Peak#	Ret. Time	Area	Area%
1	2,995	23504	1,354
2	5,513	26554	1,530
3	6,709	9827	0,566
4	6,937	14741	0,849
5	7,160	1649226	95,028
6	8,863	11664	0,672
Total		1735516	100,000

PDA Peak Table(LK197_Deg_10mM.lcd)
PDA Ch3 320nm

Peak#	Ret. Time	Area	Area%
1	2,995	21010	1,382
2	5,394	24364	1,603
3	6,708	6876	0,452
4	6,935	4299	0,283
5	7,160	1448082	95,285
6	8,862	15109	0,994
Total		1519739	100,000

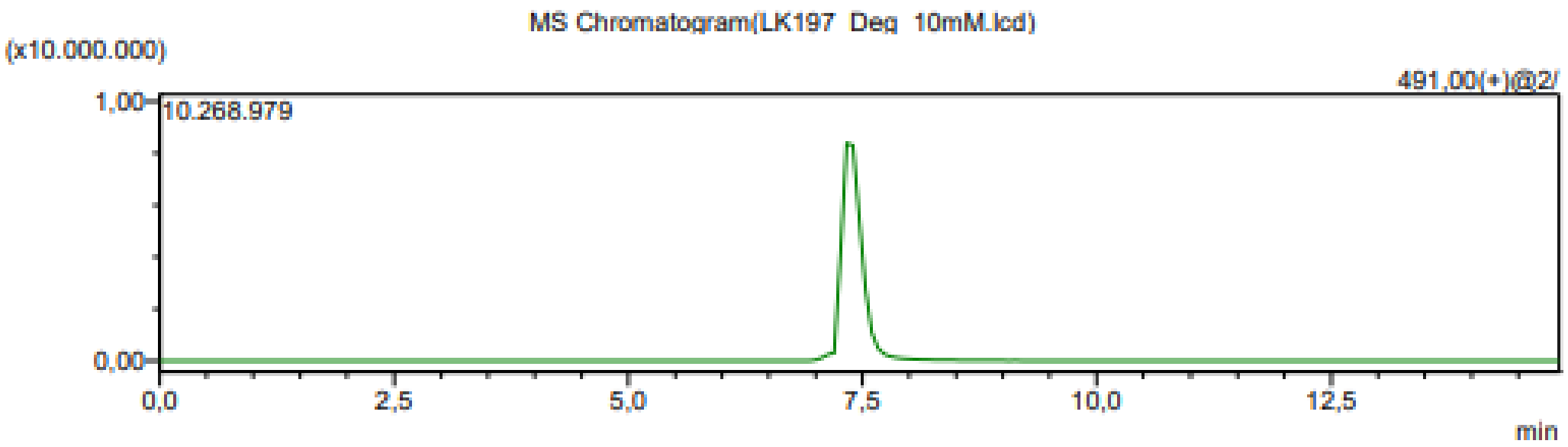

^{1}H NMR spectrum of **8** (400 MHz, $(CD_3)_2SO$)

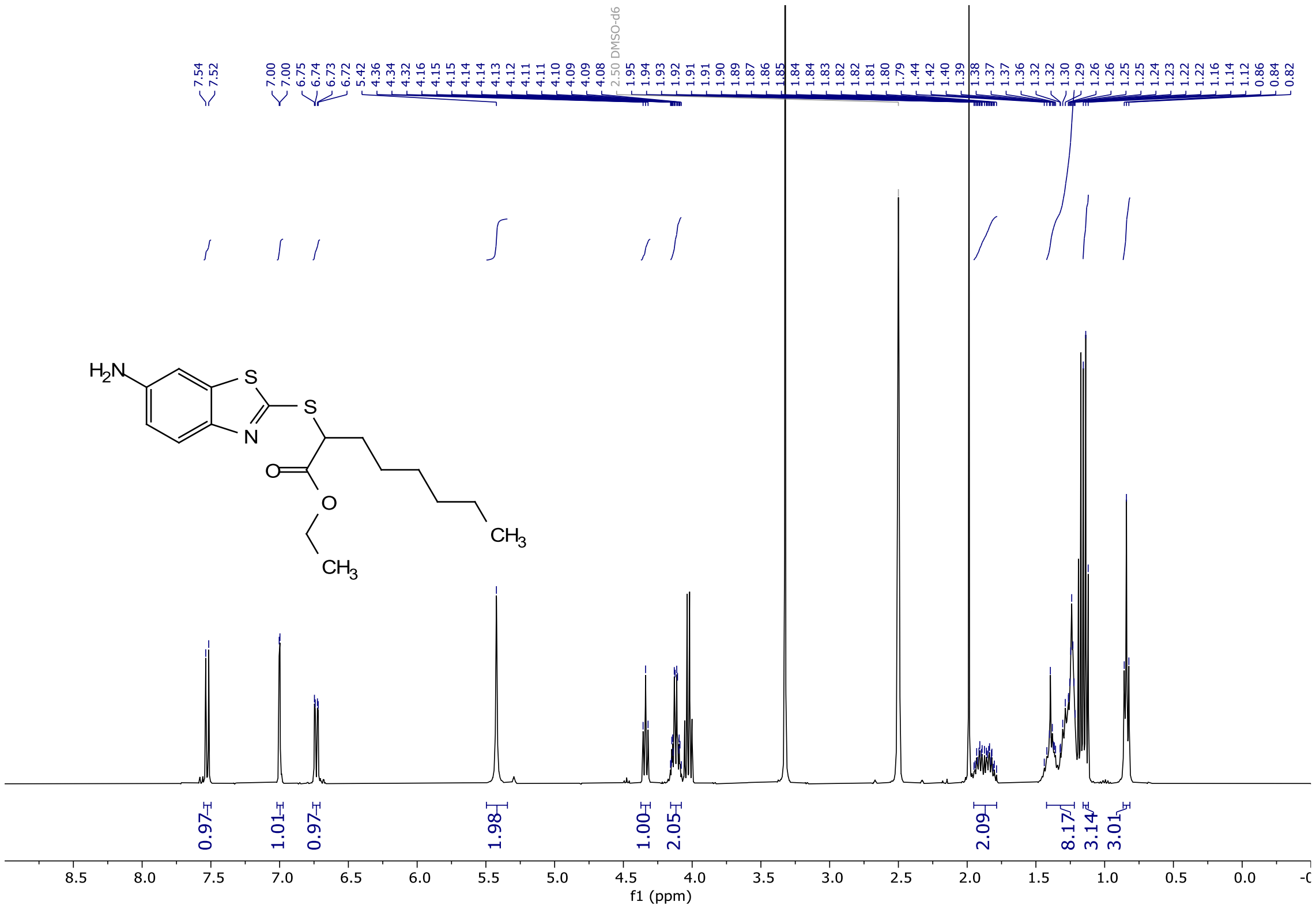

^{13}C NMR spectrum of **8** (101 MHz, $(CD_3)_2SO$)

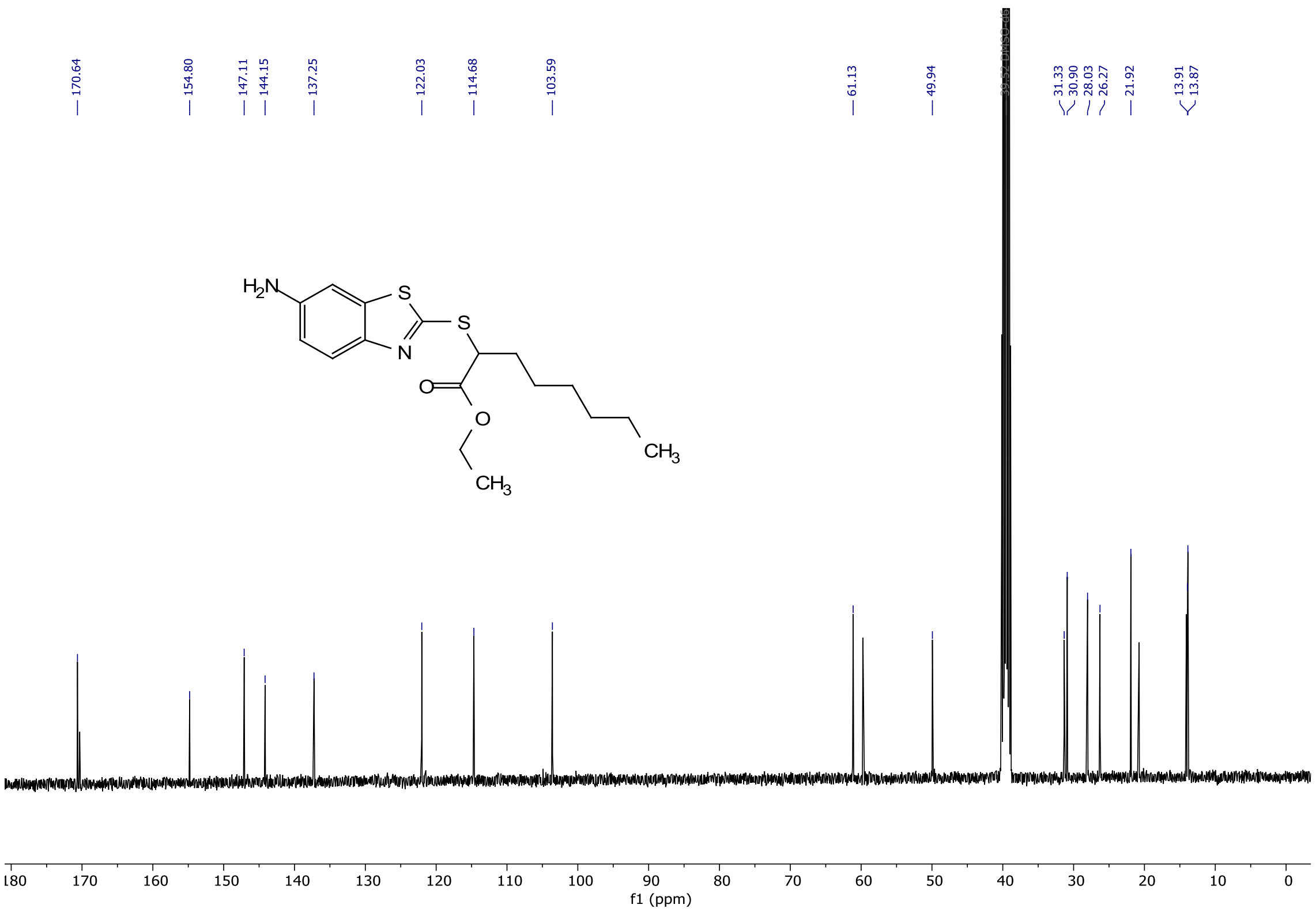

^{1}H NMR spectrum of **9** (400 MHz, $(CD_3)_2SO$)

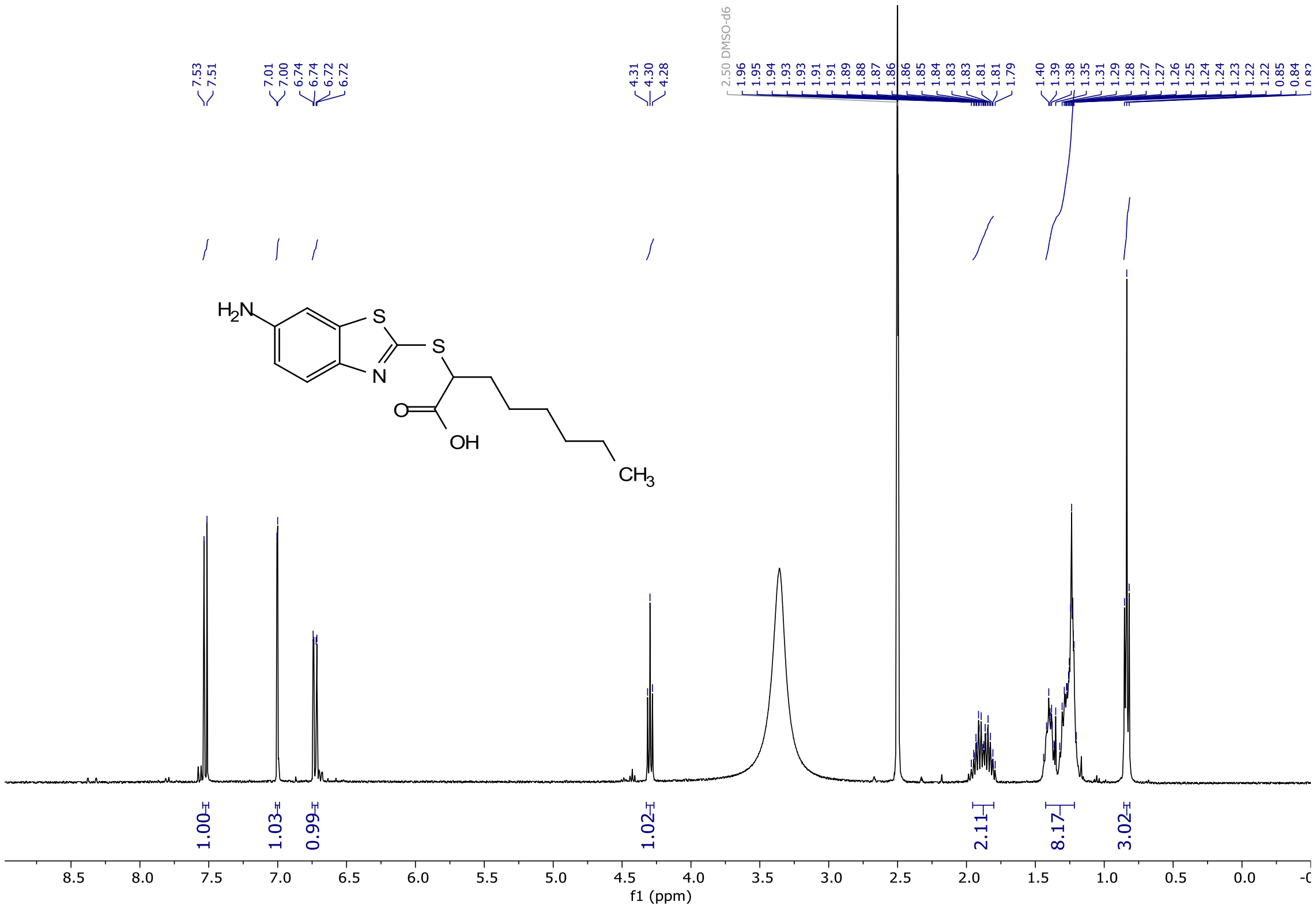

^{13}C NMR spectrum of **9** (101 MHz, $(CD_3)_2SO$)

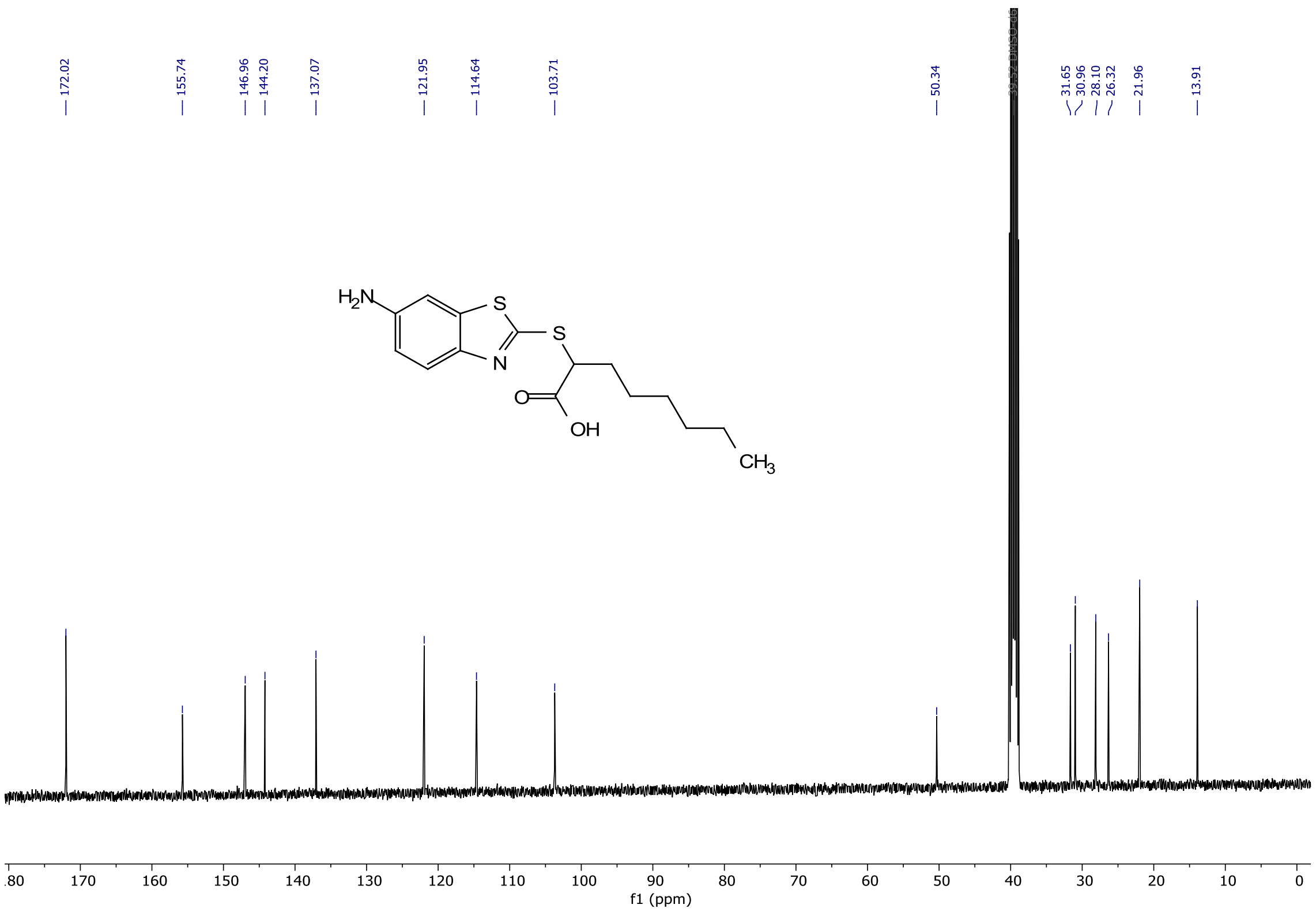

Supplementary References

[1] M. Reynders, S. Willems, J. A. Marschner, T. Wein, D. Merk, O. Thorn-Seshold, A High-Quality Photoswitchable Probe that Selectively and Potently Regulates the Transcription Factor RORγ, *Angew. Chem. Int. Ed.* **2024**, 63, e202410139, https://doi.org/10.1002/anie.202410139.

[2] B. Baumgartner, V. Glembockyte, A. Gonzalez-Hernandez, A. Valavalkar, R. Mayer, L. Fillbrook, A. Müller-Deku, J. Zhang, F. Steiner, A. Wiegand, C. Gross, M. Reynders, H. Munguba, A. Arefin, A. Ofial, J. Beves, T. Lohmüller, B. Dietzek-Ivansic, J. Broichhagen, P. Tinnefeld, J. Levitz, O. Thorn-Seshold, A general method for near-infrared photoswitching in biology, demonstrated by the >700 nm photocontrol of GPCR activity in brain slices, *ChemRxiv* **2024**, https://doi.org/10.26434/chemrxiv-2024-vm4n3.

[3] S. Arifi, J. A. Marschner, J. Pollinger, L. Isigkeit, P. Heitel, A. Kaiser, L. Obeser, G. Höfner, E. Proschak, S. Knapp, A. Chaikuad, J. Heering, D. Merk, Targeting the Alternative Vitamin E Metabolite Binding Site Enables Noncanonical PPARγ Modulation, *J. Am. Chem. Soc.* **2023**, 145, 14802–14810, https://doi.org/10.1021/jacs.3c03417.

[4] M. Borowiak, W. Nahaboo, M. Reynders, K. Nekolla, P. Jalinot, J. Hasserodt, M. Rehberg, M. Delattre, S. Zahler, A. Vollmar, D. Trauner, O. Thorn-Seshold, Photoswitchable Inhibitors of Microtubule Dynamics Optically Control Mitosis and Cell Death. *Cell* **2015**, 162, 403–411, https://doi.org/10.1016/j.cell.2015.06.049

[5] J. Vietor, C. Gege, T. Stiller, R. Busch, E. Schallmayer, H. Kohlhof, G. Höfner, J. Pabel, J. A. Marschner, D. Merk, Development of a Potent Nurr1 Agonist Tool for In Vivo Applications, *J. Med. Chem.* **2023**, 66, 6391–6402, https://doi.org/10.1021/acs.jmedchem.3c00415.

[6] Wolbank, S.; Stadler, G.; Peterbauer, A.; Gillich, A.; Karbiener, M.; Streubel, B.; Wieser, M.; Katinger, H.; Van Griensven, M.; Redl, H.; Gabriel, C.; Grillari, J.; Grillari-Voglauer, R. Telomerase Immortalized Human Amnion- and Adipose-Derived Mesenchymal Stem Cells: Maintenance of Differentiation and Immunomodulatory Characteristics. *Tissue Eng. Part A* **2009**, 15, 1843–1854, https://doi.org/10.1089/ten.tea.2008.0205.

[7] D. S. Pedersen, C. Rosenbohm, Dry Column Vacuum Chromatography, *Synthesis* **2001**, 16, 2431–2434, https://doi.org/10.1055/s-2001-18722.

[8] T. Hanke, S.Y. Cheung, W. Kilu, J. Heering, X. Ni, V. Planz, S. Schierle, G. Faudone, M. Friedrich, M. Wanior, O. Werz, M. Windbergs, E. Proschak, M. Schubert-Zsilavecz, A. Chaikuad, S. Knapp, D. Merk, A Selective Modulator of Peroxisome Proliferator-Activated Receptor γ with an Unprecedented Binding Mode, *J. Med. Chem.* **2020**, 63, 4555–4561, https://doi.org/10.1021/acs.jmedchem.9b01786.
